# A systematic review of core outcomes reported in boys and men with Klinefelter syndrome

**DOI:** 10.1007/s12020-025-04376-8

**Published:** 2025-08-16

**Authors:** Mikaela Frixou, Courtney Moffat, Xanthippi Tseretopoulou, S. Faisal Ahmed, Angela K. Lucas-Herald

**Affiliations:** Developmental Endocrinology Research Group, University of Glasgow, Royal Hospital for Children, Glasgow, UK

**Keywords:** Children, Adults, Klinefelter syndrome, 47, XXY

## Abstract

**Purpose:**

Klinefelter syndrome (XXY) has a wide range of presentations and health consequences. The aim of this systematic review was to identify potential core outcomes reported in males with XXY.

**Methods:**

Systematic searches of PubMed, Science Direct, and Cochrane were performed to source studies. The inclusion criteria were studies involving males with KS with any intervention, comparison, or outcome, with separate searches for studies reporting on children <16 years of age and for adults ≥16 years of age.

**Results:**

For children <16 years old, 56 studies met the eligibility criteria. Thirty-seven (66%) studies reported anthropometric measurements and physical characteristics. Behavioural, cognitive developmental and psychiatric outcomes were also commonly reported (27, 48%) as were biochemical results in 27 (48%) studies. Other outcomes included presence of co-morbidities (16, 29%) and fertility outcomes (10, 18%). In the studies focusing on individuals ≥16 years of age, 183 studies met the eligibility criteria. Outcomes relating to biochemistry, physical characteristics, fertility and occurrence of co-morbidities were reported in 118 (64%), 89 (49%) 65 (36%) and 62 (34%) studies respectively. Quality of life was reported least frequently in only 2 (4%) paediatric studies and 5 (3%) of adult studies.

**Conclusion:**

The present study highlights the variety of outcomes studied in boys and men with KS. These results can support the development of age-specific core outcome sets for clinical research to promote homogeneity and to aid standardised data collection.

## Introduction

Klinefelter syndrome (KS) is the most prevalent sex chromosome aneuploidy in men, affecting 1 in every 660 newborn males [1]. Boys and men with KS exhibit a diverse spectrum of clinical manifestations including hypogonadism, subfertility, cardiometabolic complications and neuropsychiatric issues. Of note, however, many men with KS are not aware they have the condition demonstrating the marked phenotypic variability. As such, the clinical outcomes reported in research into KS are very broad, leading to potential difficulties in accumulating evidence to improve scientific knowledge about the condition as well as ultimately clinical care. Notably, males with KS exhibit elevated morbidity and a 50% greater mortality risk compared to healthy age-matched men [2], thus necessitating earlier diagnosis, higher index of suspicion and lower threshold for testing for the condition in clinical practice.

Core outcome sets (COS) represent the minimum outcomes that should be included in the design of studies of specific conditions or patient groups, and aim to improve comparability of studies, improve utility of outcomes in research and clinical practice, and allow researchers and clinicians to appraise data for use in guidelines and future research [3]. COS exist for other genetic and childhood conditions such as osteogenesis imperfecta, medium-chain acyl-CoA dehydrogenase deficiency and phenylketonuria, while development of COS is in progress for many other conditions such as type 1 diabetes and growth hormone deficiency [4–7]. The lack of consistency in reported outcomes in KS research highlights the need to establish the core outcomes associated with KS to allow for increased standardisation of research in the field, with the overall aim to improve understanding of the condition, and translate this to improved clinical results for affected boys and men. As a first step towards development of core outcome sets for KS this systematic review aimed to identify outcomes reported in studies of individuals with KS. Outcomes reported consistently in >25% of studies were considered to be common and were proposed for consideration as core outcomes. This cut-off was selected based on previous systematic reviews of outcomes of other conditions such as hypospadias [8]. Outcomes found to be inconsistently reported will require further discussion and expert consensus to determine what assessment tools and outcomes will need to be used as part of the core outcome set.

## Methods

Systematic computerised literature searches were performed in PubMed, Science Direct and Cochrane between June-December 2023. These searches were based on the following PICO (Population, Intervention, Comparison, Outcome) question:

P: boy/man with diagnosis of KS

I: any

C: any

O: any

### Search strategy

Studies that met the eligibility criteria were included with no language limitations. The following key terms were searched in each database: Klinefelter syndrome OR Klinefelter’s syndrome OR 47, XXY OR Klinefelter OR Klinefelter’s. The reference lists of the latest systematic reviews and meta-analyses on the topic were also checked, and any eligible studies were included. There were no language or date restrictions imposed however the full text had to be available online. Searches were performed separately for children (<16 years) and adults (≥16 years of age). The age of 16 years was used as this is often used as the cut-off for paediatric vs. adult care in the United Kingdom.

### Study inclusion

The systematic review was performed in accordance with the Preferred Reporting Items for Systematic Reviews and Meta-analysis guidelines (PRISMA) [9]. Two members of the research team independently reviewed the titles, abstracts, and full texts of all studies identified by the search in a sequential fashion, to identify which were eligible for inclusion using the Covidence platform (Covidence systematic review software, Veritas Health Innovation, Melbourne, Australia (www.covidence.org)). Any discrepancies were resolved by discussion. The reasons for excluding abstracts and full texts were recorded. Neither of the review authors were blind to the journal titles or to the study authors or institutions. Studies were excluded if they were not about humans. Case reports were excluded if they did not have more than 2 patients. Where studies included individuals ≤16.0 years and ≥16.0 years, they were included as either paediatric or adult studies according to the mean age of the participants.

### Data extracted

For each of the included studies, the epidemiological design, the journal title, authors, year of publication, country of origin, study sample, population age, the outcome(s) assessed, the tool which was used to measure the outcome, and the frequency of the measurement were recorded.

### Quality assessment

Assessment of bias was undertaken using the relevant Critical Skills Appraisal programme checklist according to the study design [10].

## Results

### Study selection

**Fig. 1:**
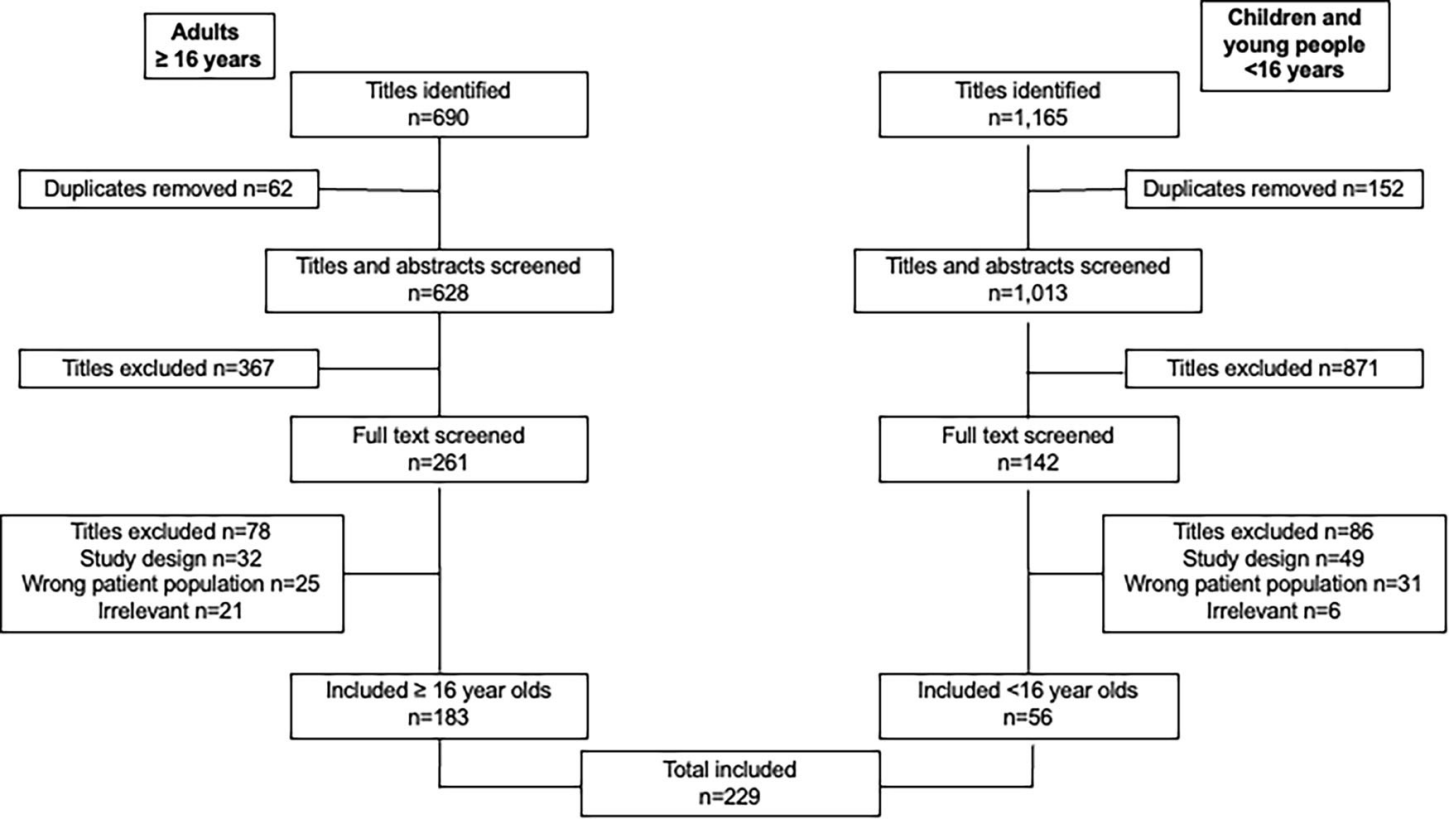
Study selection strategy flowchart in accordance with PRISMA guidelines

**Fig. 2:**
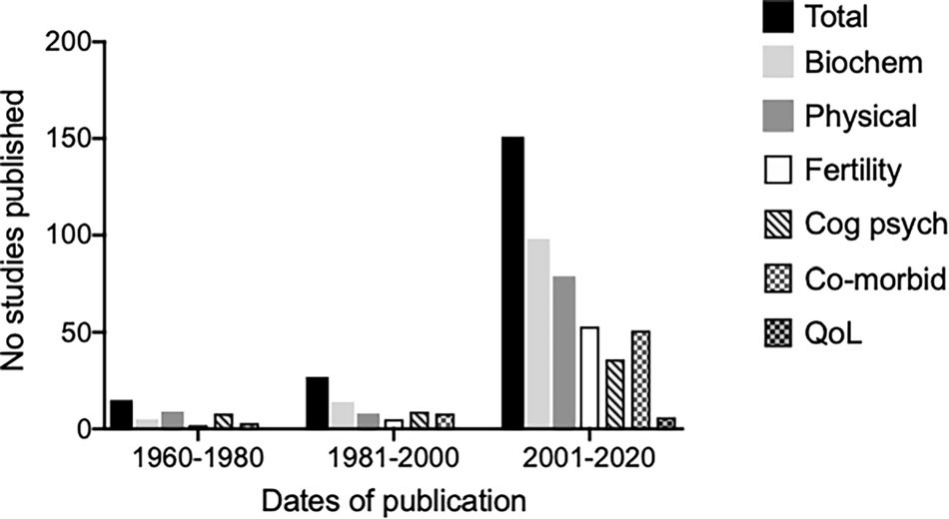
Reporting of outcomes per year in published studies of boys and men with KS since 1960. biochem biochemistry, cog psych cognitive psychiatric, co-morbid co-morbidities, QoL quality of life

**Fig. 3:**
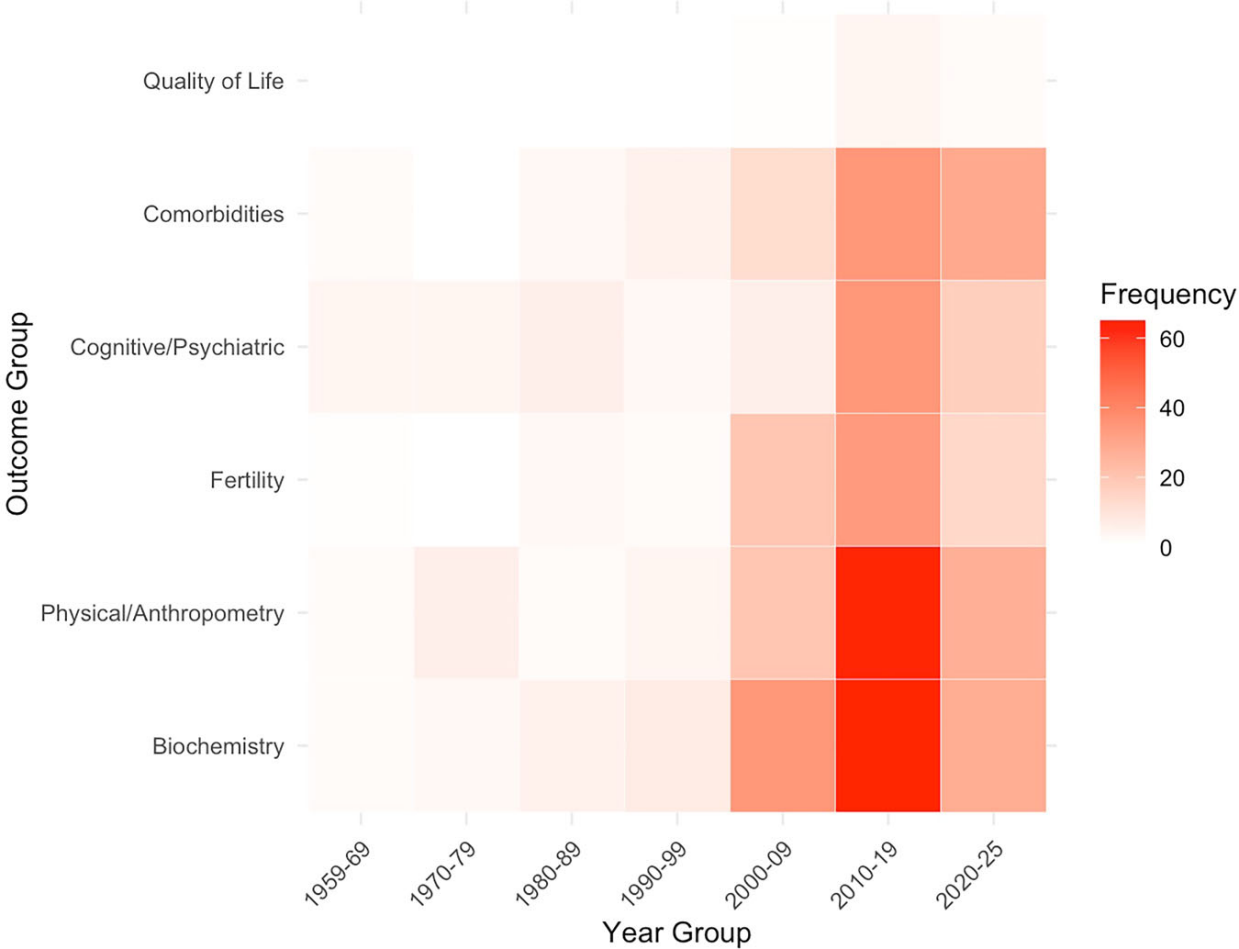
Heat map of frequency of reported outcomes per year in published studies of boys and men with KS since 1959

Using the search strategy, a total of 1855 articles were initially identified and after removal of duplicates, we screened the titles and abstracts of 1641 manuscripts. Of these, 1248 (76%) were excluded for irrelevance. A full-text evaluation was conducted on the remaining 393 articles. In total, 56 paediatric studies and 183 adult studies were eligible for data extraction and inclusion in the study (Fig. 1). A list of the references of included studies can be found in the supplementary material (Supplementary Table 1). The characteristics of the 239 eligible articles are shown in Supplementary Table 2 (paediatric studies) and Supplementary Table 3 (adult studies). Quality assessment demonstrated that the papers included were of medium-high quality and with low-medium risk of bias. Of the 30,762 participants with KS included in the studies, the overall median reported age of the participants was 33 (0, 67) years. The majority of the studies have been published in the last 20 years (Fig. 2). A heat map of reported outcomes was constructed to demonstrate frequency of reported outcomes over time (Fig. 3).

### Studies in children

Table 1 summarises the outcomes in studies in children (P1–P56, Supplementary 1). Most studies reported more than one outcome. The most common outcomes investigated related to physical characteristics or anthropometric measurements (*n* = 37, 66%); biochemical and haematological outcomes (*n* = 27, 48%) and cognitive, psychological, neurodevelopmental or behavioural outcomes (*n* = 27, 48%). Many studies reported multiple outcomes and as shown in Table 1 each of these outcomes were investigated in variable ways. For example, biochemical and haematological outcomes included various sex hormones such as testosterone (38%), oestradiol (9%) and DHEAS (4%), pituitary hormones LH (30%) and FSH (38%), inhibin B (25%), AMH (18%), and less frequently reported lipids and triglycerides, thyroid function tests, glucose, sex hormone binding globulin, haemoglobin, urinary 17-ketosteroids. Only 2 (4%) studies reported outcomes on quality of life (QOL) in young people with KS, and each study used a different assessment tool; Close et al. used the paediatric quality of life inventory, while Jordan et al. used PROMIS [11, 12]. In addition, despite the multi-system nature of KS in many individuals, only 16 studies (29%) reported co-morbidities and 10 (18%) studies reported outcomes related to fertility preservation.

**Table 1:** Summary of measurements in paediatric studies

Type of parameters n (%)	Parameter	Method of measurement	No of paediatric studies (%) *n* = 56
Phenotype/anthropometry *n* = 37 (66)	Tanner stage	Pubertal assessment	19 (34)
	Height/length	Anthropometry	14 (25)
	Penile length	Anthropometry	11 (20)
	Weight	Anthropometry	12 (21)
	Body mass index	Anthropometry	11 (20)
	Testicular volume	Pubertal assessment	13 (23)
	Gynaecomastia	Pubertal assessment	9 (16)
	Body fat	Anthropometry	7 (13)
	Bone age	X-ray	4 (7)
	Head circumference	Clinical assessment	4 (7)
	Fat-free/lean mass	Anthropometry	3 (5)
	Skeletal abnormalities	Clinical assessment	3 (5)
	Height velocity	Anthropometry	2 (4)
	Arm span	Anthropometry	3 (5)
	Waist circumference	Anthropometry	2 (4)
	Testicular length	Pubertal assessment	2 (4)
	Bone density	DXA	2 (4)
	Varicocoele	Clinical assessment	2 (4)
	Cryptorchidism	Clinical assessment	2 (4)
	Upper segment/lower segment SDS	Anthropometry	1 (2)
	Upper/lower segment ratio	Anthropometry	1 (2)
	Finger ridge counts	Clinical assessment	1 (2)
	Hand tremor	Clinical assessment	1 (2)
	Muscle tone	Clinical assessment	1 (2)
	Bone health index	DXA	1 (2)
	Muscle mass	DXA	1 (2)
	Penile width	Anthropometry	1 (2)
	Macroglossia	Clinical assessment	1 (2)
	Hypospadias	Clinical assessment	1 (2)
Biochemical/haematological *n* = 27 (48)	Testosterone	Serum	21 (38)
	FSH	Serum	21 (38)
	LH	Serum	17 (30)
	Inhibin B	Serum	14 (25)
	AMH	Serum	10 (18)
	Lipids and triglyceride	Serum	5 (9)
	Oestradiol	Serum	5 (9)
	SHBG	Serum	4 (7)
	DHEAS	Serum	2 (4)
	Glucose	Serum	2 (4)
	Haemoglobin/haematocrit	Serum	2 (4)
	Thyroid function +/− antibodies	Serum	2 (4)
	Urinary FSH	Urine	1 (2)
	Urinary 17-ketosteroids	Urine	1 (2)
	Pro-alpha C	Serum	1 (2)
Cognitive, psychological, developmental and behavioural *n* = 27 (48)	Hand preference	Observer assessment	4 (7)
	Intelligence	WISC/WAIS	13 (23)
		WRAT	3 (5)
		Other	6 (9)
	Language	PLS 4/5	2 (4)
		EOW PVT-4	2 (4)
		ROW PVT-4	2 (4)
		DAS-2	2 (4)
		Other	6 (9)
	Behaviour	CBCL	4 (7)
		BRIEF-P	3 (5)
		Conner’s	2 (4)
		Other	7 (13)
	Development	Bayley	4 (7)
		PMDS	2 (4)
		Other	3 (5)
	Psychosocial	Piers-Harris	2 (4)
		CDI	3 (5)
		Other	4 (7)
Co-morbidities *n* = 16 (29)	Neurological outcomes	Hospital data	7 (13)
	Side effects of testosterone	Hospital data	3 (5)
	Cardiovascular health	Clinical assessment	3 (5)
	Fractures	Hospital data	1 (2)
	Neonatal outcomes	Hospital data	1 (2)
	Thrombosis	Hospital data	1 (2)
Fertility *n* = 10 (18)	Testicular histology	Biopsy	6 (11)
	Sperm concentrations	Semen analysis	4 (7)
	Surgical sperm retrieval rates	TESE	1 (2)
	Testicular microlithiasis	Ultrasonography	1 (2)
Quality of life *n* = 2 (4)	Paediatric Quality of Life Inventory	Questionnaire	1 (2)
	PROMIS self-report	Questionnaire	1 (2)

Studies often reported more than one outcome

*AMH* anti-mullerian hormone, *Bayley* bayley scales of infant and toddler development, *BRIEF-P* behaviour rating inventory of executive function years preschool questionnaire, *CBCL* child behaviour checklist, *CDI* children’s depression inventory, *Conner’s* conner’s parent rating scale, *DAS-2* differential ability scale 2, *DHEAS* dehydroepiandrosterone, *DXA* dual energy X-ray absorptiometry, *EOW PVT-4* expressive one-word picture vocabulary test- 4th edition, *FSH* follicle stimulating hormone, *LH* luteinising hormone, *PDMS* peabody developmental motor scales, *Piers-Harris* piers-harris self concept scale, *PLS4/5* preschool language scale 4th/5th edition, *PROMIS* patient-reported outcomes measurement information system, *ROW PVT-4* receptive one-word picture vocabulary test- 4th edition, *SDS* standard deviation score, *SHBG* sex hormone binding globulin, *TESE* testicular sperm extraction, *WAIS* wechsler adult intelligence scale, *WISC* intelligence scale for children, *WRAT* wide range achievement test

### Studies in adults

Table 2 summarises the studies included in adults (A1-A183, Supplementary Table 1). The most common outcomes investigated were relating to biochemical and haematological outcomes (*n* = 118, 64%) including the hormones testosterone (62%), FSH (52%), LH (50%), oestradiol (28%), and prolactin (13%); physical characteristics or anthropometric measurements (*n* = 89, 49%); and fertility (*n* = 65, 36%). A variety of outcomes relating to physical co-morbidity were reported in 62 (34%) studies, with a particular focus on bone health (19 studies, 10%) and cardiovascular (18 studies, 10%) outcomes. Cognitive outcomes were reported in 47 (26%) studies, and similar to the paediatric studies, a range of tools were used (Table 2). Of these, psychiatric disorders and other mental health outcomes were reported in 34 (19%) studies. A total of 5 (3%) studies discussed aspects of QOL, and tools used included the quality of life, enjoyment and satisfaction questionnaire [13], RAND-36 health survey [14, 15], the World health organisation’s quality of life assessment- BREF [14, 16], CSQ-4 and Youth Health Care – Satisfaction, Utilization and Needs Tools [17].

**Table 2:** Summary of measurements in adult studies

Type of parameters n (%)	Parameter	Method of measurement	No of adult studies (%) *n* = 183
Biochemical/haematological *n* = 118 (64)	Testosterone	Serum	113 (62)
	FSH	Serum	96 (52)
	LH	Serum	91 (50)
	Oestradiol	Serum	52 (28)
	SHBG	Serum	35 (19)
	Prolactin	Serum	24 (13)
	Lipids and triglyceride	Serum	24 (13)
	Total cholesterol	Serum	23 (13)
	Low density lipoprotein cholesterol	Serum	15 (8)
Phenotype/anthropometry *n* = 89 (49)	Testicular volume/size	Variety of methods	54 (30)
	Height	Anthropometry	40 (22)
	Weight	Anthropometry	38 (21)
	Body Mass Index	Anthropometry	38 (21)
	Gynaecomastia	Clinical assessment	14 (8)
	Testicular morphology	Variety of methods	10 (5)
	Cryptorchidism	Clinical assessment	7 (4)
	Prostate volume/size	Variety of methods	6 (3)
	Hypospadias	Hospital data	1 (0.5)
Fertility *n* = 65 (36)	Sperm concentrations	Semen analysis	40 (22)
	Surgical sperm retrieval rates	TESE	38 (21)
	Testicular histology	Biopsy	26 (14)
	Pregnancy rate	Post SSR	23 (13)
	Fertilisation rate	Post ICSI/IVF	22 (12)
	Live birth rate	Post SSR	20 (11)
Co-morbidities *n* = 62 (34)	Cancer development	Hospital data	5 (3)
	Mortality	Population data	5 (3)
	Seizures/epilepsy	Hospital data	3 (2)
	Systemic lupus erythematosus	Hospital data	2 (1)
	Rheumatoid arthritis	Hospital data	2 (1)
	Sjogren’s syndrome	Hospital data	2 (1)
	Thyroid disease	Hospital data	2 (1)
	Thrombosis	Hospital data	2 (1)
	Renal abnormalities	Hospital data	2 (1)
	Autoimmunity	Hospital data	2 (1)
	Bone health	As below	19 (10)
	Bone mineral density	DXA	16 (9)
		PQCT	2 (1)
		Single photon absorptiometry	1 (0.5)
	Osteoporosis	DXA/X-ray	8 (4)
	Osteopenia	DXA/X-ray	5 (3)
	Vertebral fractures	DXA/X-ray	2 (1)
	Back pain	Patient report	1 (0.5)
	Cardiometabolic health	As below	18 (10)
	Diabetes	Hospital data	9 (5)
	Obesity	Hospital data	6 (3)
	Metabolic syndrome	Hospital data	2 (1)
	Dyslipidaemia	Hospital data	2 (1)
	Vascular abnormalities	Hospital data	1 (0.5)
	Cardiac abnormalities	Hospital data	1 (0.5)
	Arterial hypertension	Hospital data	1 (0.5)
	Cardiopulmonary diseases	Hospital data	1 (0.5)
	Epicardial fat thickness	Echocardiography	1 (0.5)
	Reduced artery diameters	Echocardiography	1 (0.5)
	Coronary disease	Hospital data	1 (0.5)
Cognitive, psychological, developmental and behavioural *n* = 47 (26)	Psychiatric	Patient report	34 (19)
		AQ	9 (5)
		SPQ	4 (2)
		HADS	3 (2)
		SCL-90	3 (2)
		SCL-90-R	2 (1)
		CES-D	2 (1)
		SCID-I and SCID-II	2 (1)
		Adult ADHD Self-Report	1 (0.5)
		MHQ	1 (0.5)
		Lorr and McNair	1 (0.5)
		Reading the Mind in the Eyes	1 (0.5)
		K10	1 (0.5)
	Personality trait	Revised Personality Inventory	4 (2)
		TCI-R	1 (0.5)
		MMPI	1 (0.5)
		BVAQ	1 (0.5)
	Self-esteem	RES	5 (3)
		Psychological Adaptation Scale	1 (0.5)
	Body Image	Draw A Person	2 (1)
		Body Image Scale	2 (1)
		Body Uneasiness Test	1 (0.5)
		MBSRQ-AS	1 (0.5)
		Appearance Evaluation Scale	1 (0.5)
		Appearance Orientation Scale	1 (0.5)
	Gender identity	GIDYQ-AA	1 (0.5)
		UGDS	1 (0.5)
	Coping	Coping with DSD scale	2 (1)
		Ways of Coping Checklist	2 (1)
		COPE Inventory	1 (0.5)
		PAS	1 (0.5)
	Intelligence	Wechsler Intelligence Scale	19 (10)
	Cognition	Cognitive function	14 (8)
		Learning ability	3 (2)
		Brain function to stimuli	1 (0.5)
		Communication	1 (0.5)
	Social behaviour	Range of methods	11 (6)
		Criminal conviction	1 (0.5)
	Sexualised behaviour	Interview	4 (2)
		Patient report	1 (0.5)
		ANDROtest	1 (0.5)
		Sexual Addiction Screening Test	1 (0.5)
		Criminal conviction	1 (0.5)
	Psychiatric disorders	Overall diagnosis	27 (15)
		Diagnosis depression	20 (11)
		Diagnosis anxiety	16 (9)
		Diagnosis personality disorder	13 (7)
		Diagnosis ASD	11 (6)
		Diagnosis schizophrenia	6 (3)
		Diagnosis ADHD	4 (2)
		Diagnosis neuroticism	4 (2)
		Diagnosis schizotypal symptoms	3 (2)
Sexual dysfunction *n* = 14 (8)	Erectile dysfunction	Patient report	10 (5)
		IIEF-15	6 (3)
		SIEDY	2 (1)
		IIEF-5	1 (0.5)
		AIPE	1 (0.5)
		ANDROTEST 12-item	1 (0.5)
		PEDT	1 (0.5)
			1 (0.5)
	Libido/sexual desire	Strain gauge with glass polygraph	9 (5)
	Ejaculation outcomes	Patient report	6 (3)
	Sexual satisfaction	Patient report	5 (3)
	Sexual frequency	Patient report	4 (2)
	Orgasm	Patient report	
Outcomes surgical procedures *n* = 6 (3)	Gonadectomy	Hospital data	5 (3)
	Hypospadias repair	Hospital data	3 (2)
	Mastectomy	Hospital data	3 (2)
	Orchidopexy	Hospital data	3 (2)
	Patient satisfaction post-operatively	Patient questionnaire	2 (1)
	Sex reassignment surgery	Hospital data	1 (0.5)
Outcomes surgical procedures *n* = 6 (3)	Gonadectomy	Hospital data	5 (3)
	Hypospadias repair	Hospital data	3 (2)
	Mastectomy	Hospital data	3 (2)
	Orchidopexy	Hospital data	3 (2)
	Patient satisfaction post-operatively	Patient questionnaire	2 (1)
	Sex reassignment surgery	Hospital data	1 (0.5)
Quality of life *n* = 5 (3)	Quality of life questionnaires	QoL Assessment-BREF	3 (2)
		RAND-36	1 (0.5)
		QoL, Enjoyment and Satisfaction Questionnaire	1 (0.5)
		BREF/RAND Short Form	1 (0.5)
		CSQ-4	1 (0.5)
		Youth Health Care – Satisfaction, Utilization and Needs	

Studies often reported more than one outcome

*ADHD* attention deficit/ hyperactivity disorder, *AIPE* arabic index for premature ejaculation, *AQ* autism spectrum quotient, *ASD* autism spectrum disorder, *BVAQ-2* bermond-vorst alexithymia questionnaire 2nd edition, *CES-D* center for epidemiologic studies depression scale, *COPE* coping orientation to problems experienced questionnaire, *CSQ-4* customer satisfaction questionnaire 4th edition (related to healthcare), *DSD* differences of sex development, *DXA* dual-energy X-ray absorptiometry, *FSH* follicle stimulating hormone, *GIDYQ-AA* gender identity/gender dysphoria questionnaire, *HADS* hospital anxiety and depression scale, *ICSI* intracytoplasmic sperm injection, *IIEF-5* international index of erectile dysfunction-5, *IIEF-15* international index of erectile dysfunction-15, *IVF* in vitro fertilisation, *K10* kessler psychological distress scale, *LH* luteinising hormone, *MBSRQ-AS* multidimensional body-self relations questionnaire, *MHQ* middlesex hospital questionnaire, *MMPI* multiphasic personality inventory, *PAS* psychological adaptation scale, *PEDT* premature ejaculation diagnostic tool, *PQCT* peripheral quantitative computed tomography, *QOL-BREF* quality of life brief version, *QOL* quality of life, *RAND-36* research and development 36-item health survey, *RES* rosenberg self-esteem scale, *SCID I/ SCID II SCID-I and II* structured clinical interview for diagnostic and statistical manual of mental disorders axis I and II disorders, *SCL-90* Symptom checklist-90, *SCL-90-R* symptom checklist-90-revised, *SHBG* sex hormone binding globulin, *SIEDY* structured interview on erectile dysfunction, *SPQ* schizotypal personality questionnaire, *SSR* surgical sperm retrieval, *TCI-R* temperament and character inventory-revised, *TESE* testicular sperm extraction, *UGDS* utrecht gender dysphoria scale

### Proposed core outcomes

Different potential core outcomes were identified in children <16 years compared to adult men with KS. Biochemistry results were reported commonly in both paediatric and adult studies, with testosterone and FSH being reported in >25% studies. LH was also reported in >25% of adult studies. Anthropometric and physical characteristics were commonly reported, although only Tanner stage (19, 34%) and height (14, 25%) met the threshold of 25% in paediatric studies, with considerable variability in reporting. In adults, however, testes size was reported in 54 (30%) studies. Similarly, fertility outcomes were not commonly reported in studies of children, but were reported in 65 (36%) of the adult studies. Presence of co-morbidities was also reported frequently in studies of adults with KS (62, 34%). Studies reporting cognitive, psychological, neurodevelopmental or behavioural outcomes were core outcomes for adults and children but were more common in children (27, 48%), particularly the Wechsler Intelligence Scale for Children (WISC) used as an assessment tool in 13 studies (23%). Table 3 summarises the common outcomes reported in studies to date which may form a framework for the development of a core outcome set (COS) in KS.

**Table 3:** Summary of suggested core reported outcomes in studies of children <16 and adults >16 years old with Klinefelter syndrome

Category	Children < 16 years		Adults ≥16 years	
	Individual Parameter	Number of papers (%)	Individual Parameter	Number of papers (%)
Biochemical/haematological		27 (48)		118 (64)
	Testosterone	21 (38)	Testosterone	113 (62)
	FSH	21 (38)	FSH	96 (52)
	LH	17 (30)	LH	91 (50)
	Inhibin B	14 (25)	Oestradiol	52 (28)
Phenotype/Anthropometry		37 (66)		89 (49)
	Tanner Stage Height	19 (34) 14 (25)	Testicular volume/size	54 (30)
Cognitive, psychological, developmental and behavioural		27 (48)		47 (26)
	Needs expert consensus	—	Needs expert consensus	—
Fertility		—		65 (36)
	-	—	Needs expert consensus	—
Co-morbidities		16 (29)		62 (34)
	Needs expert consensus	—	Needs expert consensus	—
Quality of Life	Needs expert consensus	—	Needs expert consensus	—

Proposed core outcomes are outcomes reported in >25% of papers

## Discussion

This systematic review summarises the outcomes described in 56 studies of children and adolescents and 183 studies of adults with KS. The most striking finding of this review was the wide variability in the reported outcomes and assessment methods. Even the commonly reported outcomes, such as anthropometry and physical characteristics were described heterogeneously, with, for example, only one single variable being reported in >25% of studies of children. Of course, the clinical presentation of KS alters across the lifetime, where different outcomes are of greater concern at different life stages, and it is estimated that only 10% of males with KS are diagnosed at childhood or adolescence [18]. Infertility and sexual dysfunction are common in adults [19]. However, management of neuro-developmental disorders relating to language and/or learning problems alongside behavioural and emotion regulation difficulties are more prominent during childhood [12]. Additionally, biochemical hallmarks of KS including testosterone, FSH, and LH levels may be normal in childhood and become abnormal following the onset of puberty [20]. Thus, different COS may be required for different age groups of people with KS as the needs of these individuals will vary across the lifespan, as demonstrated in Table 3.

Biochemical markers that were commonly reported (≥25%) in paediatric studies were testosterone, FSH, LH and Inhibin B. FSH, LH and testosterone were also reported commonly in studies of adults, along with oestradiol. Overall, measuring testosterone in boys and men with KS has a multi-faceted importance, where levels can be indicative of the need for testosterone replacement therapy, and the risk of co-morbidities, and as such it is a useful outcome to consider in boys and men with KS.

Behavioural, cognitive, neurodevelopmental, and psychiatric outcomes were frequently reported, and were included in the methodology of 31% of all included studies, highlighting these as an important part of the Klinefelter syndrome phenotype. Specifically, development, school attainment and assessment tools relating to psychological status, intelligence and social functioning were part of 27% of paediatric KS studies, while in adults 14% of studies reported cognitive outcomes, and 19% reported psychiatric and mental health-related outcomes including neurodiversity and autism spectrum disorder. Gender dysphoria was only reported in two adult studies. There is considerable variability in the tests used to assess each parameter, with >50 different assessment tools reported in included studies. Of course, it is notable that the included papers span a 64-year period, during which new evidence and tools have emerged. Additionally, the parameters tested for in some papers may be useful on an academic level to understand the disease process, but do not translate to useful clinical outcomes that are routinely tested for in clinical practice. As a result, expert opinion will be needed to reach a consensus on the most appropriate cognitive, behavioural and developmental assessment tools to standardise the monitoring of neurodevelopmental and cognitive outcomes of children with Klinefelter syndrome.

Although fertility was a commonly reported outcome in adult men with Klinefelter syndrome, only ten studies report outcomes related to fertility preservation in <16 year olds. In adults with KS, fertility outcomes were frequently studied with 36% of studies reporting these. Azoospermia was reported in 21% of studies, and semen volume, sperm concentration, sperm motility, and sperm morphology were reported in 14 (8%), 11 (6%), 10 (5%) and 3 (2%) studies respectively. Men with KS are typically infertile due to impaired spermatogenesis which often presents as azoospermia or occasionally as severe oligospermia [21]. Infertility is often the principal issue associated with KS and individuals may only present to medical services due to the need for assisted conception. Following azoospermia confirmation, KS males underwent micro-TESE in 14% of studies, TESE in 9% and TESA in 2%. Associated outcomes reported were successful sperm retrieval rate reported in 21% of studies, and 14% of studies reported testes histology findings. 12% of studies reported reproductive outcomes including clinical pregnancy rate in 13%, live birth rate in 11% and fertilisation rate in 7%. These findings highlight that future work is required to achieve a consensus in how to define infertility as an outcome, for example according to semen analysis or to the inability to father without assisted reproductive technology.

Co-morbidities were also a heterogeneously reported common outcome. KS can be accompanied with a vast array of different co-morbidities which contribute to elevated morbidity rates. Research performed by Belling et al. [22] identified 78 significant KS comorbidities following a review of hospital records. Furthermore, KS males have a 70% greater risk of being hospitalised with any diagnosis and have a lifespan truncated by 2 years compared to healthy controls [2]. That said, only 29% of paediatric studies discussed the occurrence of co-morbidities. It is essential that those in the paediatric field are aware of these conditions, so that timing of their development can be ascertained and the boys counselled about these appropriately. As such, there is a need to standardise the way in which co-morbidities are reported to raise awareness of the range of conditions associated with the XXY phenotype as well as those most prevalent, and to generate homogeneity between studies. This may have the potential to support future disease prevention, diagnosis, and treatment.

Finally, very few studies also reported QOL, despite the fact that KS males experience a vast array of manifestations which may impact many aspects of life. The studies researching this field demonstrated that QOL is impaired in KS patients compared to healthy controls [23]. As research concerning QOL is lacking, there is a need to identify the relationship between the outcomes, symptoms, and patient experiences with the QOL domains in order to support clinical trial design and in turn, improve clinical decision making in identifying how to improve QOL of KS males with variable manifestations. Notably, the aim of research and clinical care is to improve the QOL of patients. Subsequently, reporting QOL should be a priority in any research with studies reporting overall QOL if possible and should be considered as part of any COS, as per Table 3. There was variability in assessment tools of QOL have been used in included studies, and therefore expert consensus will be needed to determine which available tools may be most suitable for use in KS, or whether development of a KS-specific tool may be required.

### Strengths and limitations

This project has carried out extensive and systematic document retrieval in well-known databases and collected comprehensive relevant research as far as possible with no restrictions on date of publication or language. The sample size and research design of the studies that made up the systematic review varied substantially, as did reporting of these within the texts, and as such it was not possible to do any meta-analysis.

An additional limitation and potential source of bias refers to the fact that only the minority of the anticipated total number of males with KS are diagnosed. Therefore, the outcomes reported presently can be only applied to the established population of males with KS and those who have not been diagnosed may exhibit outcomes alternative to what has been described presently. This systematic review has taken a preliminary step in identifying the core outcomes reported in studies of boys and men with KS and contributes towards the development of a COS. However, these outcomes are only representative of diagnosed KS subjects.

Following the identification of core outcomes associated with KS in the present review, direct future work should implement the Delphi technique to rate and achieve expert consensus on the importance of these outcomes for subsequent inclusion in a COS, as demonstrated in Table 3 [24]. Ultimately, completion of the Delphi technique could lead to a definitive COS for KS which could be used to determine which outcomes should be measured in clinical trials.

#### The future of KS research

Fertility difficulties are one of the most common presenting complaints in men with Klinefelter syndrome. A large proportion of adult studies focused on fertility outcomes to determine the optimal age and intervention for fertility preservation in Klinefelter syndrome. With advancements in reproductive technology, it is estimated that sperm retrieval or pregnancy is achievable in about 50% of men with KS [25]. Although this represents an improvement in fertility rates, there remains a need for further development of reproductive technologies to improve fertility outcomes in men with Klinefelter syndrome.

As genetic testing becomes more advanced and widely accessible, it is anticipated that the number of diagnoses of KS in childhood may increase. This may allow more studies in co-morbidities in childhood and optimisation of support for improved cognitive and developmental outcomes. Additionally, as KS is associated with multiple co-morbidities and an average reduction in life expectancy by two years, studies in paediatric and young adult populations may improve quantity and quality of life by studying mechanisms and interventions in co-morbidities.

Finally, quality of life is undoubtfully an important outcome for individuals with the condition but is underreported in published studies. Development of a KS-specific quality of life questionnaire, or validation of existing QoL assessment tools for use in KS is needed to inform future studies in the condition.

To achieve more meaningful outcomes in the future, expert consensus will be needed to finalise a list of core outcome sets in children and adults with KS, to supplement findings by this systematic review.

## Conclusions

In conclusion, there is significant variability in the reported outcomes of studies in children and men with KS. The variability in measurement and assessment tools used for each outcome, highlights a lack of consensus in study design and the need for the development of COS to guide future research. Based on the included studies, initial suggestions for core outcomes for reporting in the field of KS should be related to biochemical assessments, the presence of co-morbidities, anthropometric measurements and psychosocial assessment. Development of a COS will improve the comparability and consistency of results across different studies, enhancing the quality of evidence and facilitating evidence-based decision-making for the care of boys and men with KS.

## Supplementary information

Supplementary Tables

## Data Availability

No datasets were generated or analysed during the current study.
