# Supplementary material for: A systematic review of core outcomes reported in boys and men with Klinefelter syndrome
Source: Endocrine. 2025 Aug 16;90(2):427–38. doi: 10.1007/s12020-025-04376-8 (PMC12572081; doi:10.1007/s12020-025-04376-8)
Supplement: Supplementary file 1 — Supplementary Tables [file 12020_2025_4376_MOESM1_ESM.docx]

| **Reference Number** | **Full Reference** |
| --- | --- |
| ***Paediatric studies*** | |
| P1 | Ferguson-Smith MA. The prepubertal testicular lesion in chromatin-positive Klinefelter's syndrome (primary micro-orchidism) as seen in mentally handicapped children. *Lancet*. Jan 31 1959;**1**(7066):219-22. doi:10.1016/s0140-6736(59)90049-2 |
| P2 | Johnson H, Myhre S, Ruvalcaba R, Thuline H, Kelley V. Effects of testosterone on body image and behavior in Klinefelter's syndrome: A pilot study. *Developmental Medicine & Child Neurology*. 1970;**12**(4):454-460. |
| P3 | Netley C, Rovet J. Handedness in 47,XXY Males. The Lancet1982. p. 267. |
| P4 | Netley C, Rovet J. Verbal deficits in children with 47,XXY and 47,XXX karyotypes: a descriptive and experimental study. *Brain Lang*. Sep 1982;**17**(1):58-72. doi:10.1016/0093-934x(82)90005-0 |
| P5 | Ratcliffe SG, Tierney IR. 47 XXY males and handedness. *Lancet*. Sep 25 1982;**2**(8300):716. doi:10.1016/s0140-6736(82)90736-x |
| P6 | Webber ML, Puck MH, Maresh MM, Goad WB, Robinson A. Short communication: skeletal maturation of children with sex chromosome abnormalities. *Pediatr Res*. May 1982;**16**(5):343-346. doi:10.1203/00006450-198205000-00004 |
| P7 | Bender BG, Puck MH, Salbenblatt JA, Robinson A. Hemispheric organization in 47,XXY boys. *Lancet*. Jan 15 1983;**1**(8316):132. doi:10.1016/s0140-6736(83)91779-8 |
| P8 | Netley C, Rovet J. Hemispheric lateralization in 47,XXY Klinefelter's syndrome boys. *Brain Cogn*. Jan 1984;**3**(1):10-18. doi:10.1016/0278-2626(84)90002-2 |
| P9 | Netley C, Rovet J. Relations between a dermatoglyphic measure, hemispheric specialization, and intellectual abilities in 47,XXY males. *Brain Cogn*. Apr 1987;**6**(2):153-160. doi:10.1016/0278-2626(87)90116-3 |
| P10 | Rovet J, Netley C, Keenan M, Bailey J, Stewart D. The psychoeducational profile of boys with Klinefelter syndrome. *J Learn Disabil*. Mar 1996;**29**(2):180-196. doi:10.1177/002221949602900208 |
| P11 | Lahlou N, Fennoy I, Carel JC, Roger M. Inhibin B and anti-Müllerian hormone, but not testosterone levels, are normal in infants with nonmosaic Klinefelter syndrome. *J Clin Endocrinol Metab*. Apr 2004;**89**(4):1864-1868. doi:10.1210/jc.2003-031624 |
| P12 | Wikström AM, Raivio T, Hadziselimovic F, Wikstrom S, Tuuri T, Dunkel L.  Klinefelter syndrome in adolescence: onset of puberty is associated with accelerated germ cell depletion. J Clin Endocrinol Metab. May 2004;89(5):2263-70. doi:10.1210/jc.2003-031725 |
| P13 | Wikström AM, Bay K, Hero M, Andersson AM, Dunkel L. Serum insulin-like factor 3 levels during puberty in healthy boys and boys with Klinefelter syndrome. *J Clin Endocrinol Metab*. Nov 2006;**91**(11):4705-4708. doi:10.1210/jc.2006-0669 |
| P14 | Bastida MG, Rey RA, Bergada I, et al. Establishment of testicular endocrine function impairment during childhood and puberty in boys with Klinefelter syndrome. Clin Endocrinol (Oxf). Dec 2007;67(6):863-70. doi:10.1111/j.1365-2265.2007.02977.x |
| P15 | Fine RG, Paducah DA. Preservation of spermatogenesis in adolescents with Klinefelter syndrome. The Journal of Urology. 2008 Apr;179(4S):98-9. *The Journal of Urology*. 18 May 2008 2008;**179**(4S):98-99. |
| P16 | Zeger MP, Zinn AR, Lahlou N, Ramos P, Kowal K, Samango-Sprouse C, Ross JL. Effect of ascertainment and genetic features on the phenotype of Klinefelter syndrome. *J Pediatr*. May 2008;**152**(5):716-722. doi:10.1016/j.jpeds.2007.10.019 |
| P17 | Honig SC, Sadeghi-Nejad H. Testicular Sperm Extraction (TESE) in the Adolescent Klinefelter's Patient: Standard of Care or not Ready for Prime Time? *The Journal of Urology*. 2009;**181**(4S):734. |
| P18 | Gies I, De Schepper J, Van Saen D, Anckaert E, Goossens E, Tournaye H. Failure of a combined clinical- and hormonal-based strategy to detect early spermatogenesis and retrieve spermatogonial stem cells in 47,XXY boys by single testicular biopsy. Hum Reprod. Apr 2012;27(4):998-1004. doi:10.1093/humrep/des002 |
| P19 | Ross JL, Roeltgen DP, Kushner H, Zinn AR, Reiss A, Bardsley MZ, McCauley E, Tartaglia N. Behavioral and social phenotypes in boys with 47,XYY syndrome or 47,XXY Klinefelter syndrome. *Pediatrics*. Apr 2012;**129**(4):769-778. doi:10.1542/peds.2011-0719 |
| P20 | Tincani BJ, Mascagni BR, Pinto RD, et al. Klinefelter syndrome: an unusual diagnosis in pediatric patients. J Pediatr (Rio J). Jul 2012;88(4):323-7. doi:10.2223/JPED.2208 |
| P21 | Van Saen D, Gies I, De Schepper J, Tournaye H, Goossens E. Can pubertal boys with Klinefelter syndrome benefit from spermatogonial stem cell banking? Hum Reprod. Feb 2012;27(2):323-30. doi:10.1093/humrep/der425 |
| P22 | Rives N, Milazzo JP, Perdrix A, Castanet M, Joly-Hélas G, Sibert L, Bironneau A, Way A, Macé B. The feasibility of fertility preservation in adolescents with Klinefelter syndrome. *Hum Reprod*. Jun 2013;**28**(6):1468-1479. doi:10.1093/humrep/det084 |
| P23 | Mehta A, Clearman T, Paduch DA. Safety and efficacy of testosterone replacement therapy in adolescents with Klinefelter syndrome. *J Urol*. May 2014;**191**(5 Suppl):1527-1531. doi:10.1016/j.juro.2013.09.015 |
| P24 | Ross J, Thodberg HH, Bardsley M, Gosek A. Decreased Bone Density in Boys with Klinefelter Syndrome: Results of a Placebo-Controlled Clinical Trial Using Low-Dose Androgen Treatment for 2 Years. ESPE; 2014. |
| P25 | Bardsley MD, Kowal K, Gamber RA, Lahlou N, Ross J. Androgen replacement in boys with 47,XXY Klinefelter syndrome: influence on the testicular phenotype. *Endocr Rev*. 2014;**35** |
| P26 | Brandenburg-Goddard MN, van Rijn S, Rombouts SA, Veer IM, Swaab H. A comparison of neural correlates underlying social cognition in Klinefelter syndrome and autism. *Soc Cogn Affect Neurosci*. Dec 2014;**9**(12):1926-1933. doi:10.1093/scan/nst190 |
| P27 | Lahlou ND, S., Bardsley MP, Temple MC, Kowal K, Roger M, Ross J. L’étude longitudinale de 93 garçons avec syndrome de Klinefelter met en évidence un déficit testiculaire antepubertaire. InAnnales d'Endocrinologie 2015 Sep 1 (Vol. 76, No. 4, p. 327). Elsevier Masson.  . *Annales d'Endocrinologie*. 1 September 2015 2015;**76**(4):327. |
| P28 | Close S, Fennoy I, Smaldone A, Reame N. Phenotype and Adverse Quality of Life in Boys with Klinefelter Syndrome. *J Pediatr*. Sep 2015;**167**(3):650-657. doi:10.1016/j.jpeds.2015.06.037 |
| P29 | Dotters-Katz SK, Humphrey WM, Senz KL, Lee VR, Shaffer BL, Caughey AB. The impact of prenatally diagnosed Klinefelter Syndrome on obstetric and neonatal outcomes. *Eur J Obstet Gynecol Reprod Biol*. Aug 2016;**203**:173-176. doi:10.1016/j.ejogrb.2016.05.006 |
| P30 | Davis SM, Reynolds R, Martin S, Howell S, Nokoff NJ, Zeitler PS, Tartaglia NR. A Short Course of Testosterone in Infants with 47,XXY Klinefelter syndrome Has Acute Effects on Body composition. *Horm Res Paediatr*. 1 January 2017 2017;**88**:373. |
| P31 | Davis SM, Cox-Martin MG, Bardsley MZ, Kowal K, Zeitler PS, Ross JL. Effects of Oxandrolone on Cardiometabolic Health in Boys With Klinefelter Syndrome: A Randomized Controlled Trial. *J Clin Endocrinol Metab*. Jan 01 2017;**102**(1):176-184. doi:10.1210/jc.2016-2904 |
| P32 | Ross JL, Kushner H, Kowal K, Bardsley M, Davis S, Reiss AL, Tartaglia N, Roeltgen D. Androgen Treatment Effects on Motor Function, Cognition, and Behavior in Boys with Klinefelter Syndrome. *J Pediatr*. Jun 2017;**185**:193-199.e4. doi:10.1016/j.jpeds.2017.02.036 |
| P33 | Akcan N, Poyrazoglu S, Bas F, Bundak R, Darendeliler F. Klinefelter Syndrome in Childhood: Variability in Clinical and Molecular Findings. J Clin Res Pediatr Endocrinol. Jun 1 2018;10(2):100-107. doi:10.4274/jcrpe.5121 |
| P34 | Davis SM, Lahlou N, Cox-Martin M, Kowal K, Zeitler PS, Ross JL. Oxandrolone Treatment Results in an Increased Risk of Gonadarche in Prepubertal Boys With Klinefelter Syndrome. *J Clin Endocrinol Metab*. Sep 01 2018;**103**(9):3449-3455. doi:10.1210/jc.2018-00682 |
| P35 | Davis S, Herstic A, Reynolds R, Nokoff N, Zeitler P, Tartaglia N. Testosterone Treatment Normalises Body Fat in Infants with XXY/Klinefelter  syndrome. *Journal of Investigative Medicine*. 1 January 2018 2018;**66**(1):165. |
| P36 | St John M, Ponchard C, van Reyk O, Mei C, Pigdon L, Amor DJ, Morgan AT. Speech and language in children with Klinefelter syndrome. *J Commun Disord*. 2019;**78**:84-96. doi:10.1016/j.jcomdis.2019.02.003 |
| P37 | Davis SM, Reynolds RM, Dabelea DM, Zeitler PS, Tartaglia NR. Testosterone treatment in infants with 47, XXY: effects on body composition. . *Journal of the Endocrine Society*. 2019;**3**(12):2276-2285. |
| P38 | Foland-Ross LC, Ross JL, Reiss AL. Androgen treatment effects on hippocampus structure in boys with Klinefelter syndrome. *Psychoneuroendocrinology*. Feb 2019;**100**:223-228. doi:10.1016/j.psyneuen.2018.09.039 |
| P39 | Brooks M, Samango-Sprouse C, Sadeghin T, Gropman A. The effect of Early Hormonal Treatment (EHT) on neuromotor capabilities in infants and toddlers with 47, XXY. . *Molecular Genetics and Metabolism*. 1 April 2021 2021;**132**(S63) |
| P40 | Butler G. Incidence of gynaecomastia in Klinefelter syndrome adolescents and outcome of testosterone treatment. Eur J Pediatr. Oct 2021;180(10):3201-3207. doi:10.1007/s00431-021-04083-2 |
| P41 | Samango-Sprouse C, Brooks MR, Lasutchinkow P, Sadeghin T, Powell S, Hamzik MP, Song S, Gropman AL. The effect of early hormonal treatment (EHT) on expressive and receptive language capabilities in boys with 47,XXY (Klinefelter syndrome) during infancy and early childhood. *Genet Med*. Jun 2021;**23**(6):1017-1022. doi:10.1038/s41436-021-01098-w |
| P42 | Foland-Ross LC, Gil M, Shrestha SB, Chromik LC, Hong D, Reiss AL. Cortical gray matter structure in boys with Klinefelter syndrome. *Psychiatry Res Neuroimaging*. Jul 30 2021;**313**:111299. doi:10.1016/j.pscychresns.2021.111299 |
| P43 | Hamzik M, Gropman A, Sadeghin T, Samango-Sprouse C. Novel neurocognitive profile in a minority of males with 47, XXY, Klinefelter syndrome: etiology unknown. *Molecular Genetics and Metabolism*. 1 April 2021 2021;**132**:S181-182. |
| P44 | Vogiatzi MG, Davis SM, Ross JL. Cortical Bone Mass is Low in Boys with Klinefelter Syndrome and Improves with Oxandrolone. *J Endocr Soc*. Apr 01 2021;**5**(4):bvab016. doi:10.1210/jendso/bvab016 |
| P45 | Funke M, Yang Y, Lahtinen A, Benninghoven-Frey K, Kliesch S, Neuhaus N, Stukenborg JB, Jahnukainen K. Z-scores for comparative analyses of spermatogonial numbers throughout human development. *Fertil Steril*. Sep 2021;**116**(3):713-720. doi:10.1016/j.fertnstert.2021.04.019 |
| P46 | Tanner M, Miettinen PJ, Hero M, Toppari J, Raivio T. Onset and progression of puberty in Klinefelter syndrome. Clin Endocrinol (Oxf). Mar 2022;96(3):363-370. doi:10.1111/cen.14588 |
| P47 | Samango-Sprouse C, Song S, Sampson K, Sadeghin T, Gropman A. eP230: The effects of early androgen therapy on behavior in boys with 47, XXY under 6 years old. *Genetics in Medicine*. 1 March 2022 2022;**24**(3):S143-144. |
| P48 | Brooks M, Samango-Sprouse C, Sadeghin T, Gropman A. eP089: 47, XXY: Mathematic capabilities and the impact of Hormonal Replacement Treatment (HRT). *Genetics in Medicine*. 1 March 2022 2022;**24**(3):S58-59. |
| P49 | Davis SM, Nokoff NJ, Furniss A, Pyle L, Valentine A, Fechner P, Ikomi C, Magnusen B, Nahata L, Vogiatzi MG, Dempsey A. Population-based Assessment of Cardiometabolic-related Diagnoses in Youth With Klinefelter Syndrome: A PEDSnet Study. *J Clin Endocrinol Metab*. 2022;**107**(5):e1850-e1859. doi:10.1210/clinem/dgac056 |
| P50 | Davis S, Brown M, Thompson T, Janusz J, Wilson R, Hall A, Reynolds R, Zeitler P, Tartaglia N. Testosterone Effects on Short-Term Outcomes (TESTO) Trial in Infants with XXY. *Horm Res Paediatr*. 2022;**95**(Suppl 1):115-115. |
| P51 | Samango-Sprouse C, Taylor A, Counts D, Sadeghin T, Lang L, Gropman A. Samango-Sprouse C, Taylor A, Counts D, Sadeghin T, Lang L, Gropman A. P288: A comprehensive investigation of the anthropomorphic measurements in males with 47, XXY and the impact of early hormonal treatment (EHT). Genetics in Medicine Open. 2023 Jan 1;1(1). *Genetics in Medicine Open*. 1 January 2023 2023;**1**(1) |
| P52 | Davis S, Brown M, Howell S, Janusz J, Lahloo N, Pyle L, Reynolds R, Wilson R, Zeitler P, Tartaglia N. P153: Testosterone effects on short-term physical, hormonal, and neurodevelopmental outcomes in infants with 47, XXY/Klinefelter syndrome: The TESTO Study. . *Genetics in Medicine Open*. 1 January 2023 2023;**1**(1) |
| P53 | Taylor A, Gropman A, Sadeghin T, Samango-Sprouse C. P324: A comprehensive investigation in the anthropomorphic measurements of males with 47, XXY (Klinefelter syndrome) from 6-18 years. *Genetics in Medicine Open*. 1 January 2023 2023;**1**(1) |
| P54 | Jordan TL, Foland-Ross LC, Wun VL, Ross JL, Reiss AL. Cognition, Academic Achievement, Adaptive Behavior, and Quality of Life in Child and Adolescent Boys with Klinefelter Syndrome. *J Dev Behav Pediatr*. Sep 01 2023;**44**(7):e476-e485. doi:10.1097/DBP.0000000000001201 |
| P55 | Pozza C, Sesti F, Tenuta M, et al. Testicular Dysfunction in 47,XXY Boys: When It All Begins. A Semilongitudinal Study. J Clin Endocrinol Metab. Sep 18 2023;108(10):2486-2499. doi:10.1210/clinem/dgad205 |
| P56 | Spiekermann J, Sinningen K, Hanusch B, Kleber M, Schündeln MM, Kiewert C, Siggelkow H, Höppner J, Grasemann C. Cardiorespiratory fitness in adolescents and young adults with Klinefelter syndrome - a pilot study. *Front Endocrinol (Lausanne)*. 2023;**14**:1106118. doi:10.3389/fendo.2023.1106118 |
| ***Adult studies*** | |
| A1 | Mosier HD, Scott LW, Dingman HF. Sexually deviant behavior in Klinefelter's syndrome. *The Journal of Pediatrics*. 1960;**57**(3):479-483. |
| A2 | Rohde RA. Chromatin-positive Klinefelter's syndrome: Clinical and cytogenetic studies. *Journal of Chronic Diseases*. 1963;**16**(11):1139-1149. |
| A3 | Money J. Two cytogenetic syndromes: Psychologic comparisons 1. Intelligence and specific-factor quotients. *Journal of Psychiatric Research*. 1964;**2**(3):223-231. |
| A4 | Vallotton M, Forbes A. Autoimmunity in gonadal dysgenesis and Klinefelter's syndrome. *The Lancet*. 1967;**289**(7491):648-651. |
| A5 | Myhre S, Ruvalcaba R, Johnson H, Thuline HC, Kelley V. The effects of testosterone treatment in Klinefelter's syndrome. *The Journal of pediatrics*. 1970;**76**(2):267-276. |
| A6 | Stewart-Bentley M, Horton R. Leydig cell function in Klinefelter's syndrome. *Metabolism*. 1973;**22**(7):875-884. |
| A7 | Money J, Annecillo C, Orman BV, Borgaonkar DS. Cytogenetics, hormones and behavior disability: comparison of XYY and XXY syndromes. *Clinical Genetics*. 1974;**6**(5):370-382. |
| A8 | Kosowicz J, Rzymski K. Radiological features of the skull in Klinefelter's syndrome and male hypogonadism. *Clinical radiology*. 1975;**26**:371-378. |
| A9 | Wang C, Baker H, De Kretser D, Hudson B. Hormonal studies in Klinefelter's syndrome. *Clinical endocrinology*. 1975;**4**(4):399-411. |
| A10 | Philip J, Lundsteen C, Owen D, Hirschhorn K. The frequency of chromosome aberrations in tall men with special reference to 47, XYY and 47, XXY. *American Journal of Human Genetics*. 1976;**28**(4):404. |
| A11 | Boisen E. Testicular size and shape of 47, XYY and 47, XXY men in a double-blind, double-matched population survey. *American Journal of Human Genetics*. 1979;**31**(6):697. |
| A12 | Umapathy E, Manimekalai S, Govindarajulu P. Lipid pattern in split ejaculate and Klinefelter’s syndrome. *Fertility and Sterility*. 1980;**33**(3):294-296. |
| A13 | Cheikh IE, Hamilton BP, Hsu TH, Wiswell JG. Studies using releasing factors in Klinefelter's Syndrome: The responses of LH and FSH to luteinizing hormone-releasing hormone and of prolactin and TSH to thyrotropin-releasing hormone before and after testosterone. *Psychoneuroendocrinology*. 1981;**6**(1):37-44. |
| A14 | Nistal M, Paniagua R, Abaurrea MA, Santamaría L. Hyperplasia and the immature appearance of Sertoli cells in primary testicular disorders. *Human Pathology*. 1982;**13**(1):3-12. |
| A15 | Wu F, Bancroft J, Davidson D, Nicol K. The behavioural effects of testosterone undecanoate in adult men with Klinefelter's syndrome: a controlled study. *Clinical Endocrinology*. 1982;**16**(5):489-497. |
| A16 | Paniagua R, Nistal M, Bravo MP. Leydig cell types in primary testcular disorders. *Human pathology*. 1984;**15**(2):181-190. |
| A17 | Goffi S, Isaia GC, Molinatti G, Massara F. Effect of naloxone on gonadotropin secretion before and after testosterone in Klinefelter's syndrome. *Psychoneuroendocrinology*. 1985;**10**(3):337-344. |
| A18 | Bizzarro A, Valentini G, MARTINO GD, DaPonte A, DE BELLIS A, Iacono G. Influence of testosterone therapy on clinical and immunological features of autoimmune diseases associated with Klinefelter’s syndrome. *J Clin Endocrinol Metab*. 1987;**64**(1):32-36. |
| A19 | Horowitz M, Wishart J, O'Loughlin P, Morris H, Needt A, Nordin B. Osteoporosis and Klinefelter's syndrome. *Clinical endocrinology*. 1992;**36**(1):113-118. |
| A20 | Laine T, Alvesalo L, Lammi S. A study in 47, XYY men of the expression of sex-chromosome anomalies in dental occlusion. *Archives of oral biology*. 1992;**37**(11):923-928. |
| A21 | Babić M, Šćepan I, Mićić M. Comparative cephalometric analysis in patients with X-chromosome aneuploidy. *Archives of oral biology*. 1993;**38**(2):179-183. |
| A22 | Choi HR, Lim SK, Lee MS. Site-specific effect of testosterone on bone mineral density in male hypogonadism. *Journal of Korean medical science*. 1995;**10**(6):431-435. |
| A23 | Luisetto G, Mastrogiacomo I, Bonanni G, Pozzan G, Botteon S, Tizian L, Galuppo P. Bone mass and mineral metabolism in Klinefelter's syndrome. *Osteoporosis international*. 1995;**5**:455-461. |
| A24 | Luboshitzky R, Wagner O, Lavi S, Herer P, Lavie P. Abnormal melatonin secretion in hypogonadal men: the effect of testosterone treatment. *Clinical endocrinology*. 1997;**47**(4):463-469. |
| A25 | Yoshida A, Miura K, Nagao K, Hara H, Ishii N, Shirai M. Sexual function and clinical features of patients with Klinefelter's syndrome with the chief complaint of male infertility. *international journal of andrology*. 1997;**20**(2):80-85. |
| A26 | Leifke E, Körner H-C, Link TM, Behre HM, Peters PE, Nieschlag E. Effects of testosterone replacement therapy on cortical and trabecular bone mineral density, vertebral body area and paraspinal muscle area in hypogonadal men. *European Journal of Endocrinology*. 1998;**138**(1):51-58. |
| A27 | Tatum IV WO, Passaro EA, Elia M, Guerrini R, Gieron M, Genton P. Seizures in Klinefelter’s syndrome. *Pediatric neurology*. 1998;**19**(4):275-278. |
| A28 | Tateno T, Sasagawa I, Ichiyanagi O, Ashida J, Nakada T, Saito H, Hiroi M. Microdeletion of the DAZ (deleted in azoospermia) gene or the YRRM (Y chromosome ribonucleic acid recognition motif) gene does not occur in patients with Klinefelter’s syndrome with and without spermatogenesis. *Fertility and sterility*. 1999;**71**(4):746-749. |
| A29 | Bremner WJ, Zhang G-Y, Gu Y-Q, Ge Z-Y. Quantitative and qualitative changes in serum luteinizing hormone after injectable testosterone undecanoate treatment in hypogonadal men. *Asian Journal of Andrology*. 2000;**2**:65-71. |
| A30 | Levron J, Aviram-Goldring A, Madgar I, Raviv G, Barkai G, Dor J. Sperm chromosome analysis and outcome of IVF in patients with non-mosaic Klinefelter’s syndrome. *Fertility and sterility*. 2000;**74**(5):925-929. |
| A31 | Patwardhan A, Eliez S, Bender B, Linden M, Reiss A. Brain morphology in Klinefelter syndrome: extra X chromosome and testosterone supplementation. *Neurology*. 2000;**54**(12):2218-2223. |
| A32 | De Rosa M, Paesano L, Nuzzo V, Zarrilli S, Del Puente A, Oriente P, Lupoli G. Bone mineral density and bone markers in hypogonadotropic and hypergonadotropic hypogonadal men after prolonged testosterone treatment. *Journal of Endocrinological Investigation*. 2001;**24**:246-252. |
| A33 | Levron J, Aviram-Goldring A, Madgar I, Raviv G, Barkai G, Dor J. Studies on sperm chromosomes in patients with severe male factor infertility undergoing assisted reproductive technology treatment. *Molecular and Cellular Endocrinology*. 2001;**183**:S23-S28. |
| A34 | Van den Bergh J, Hermus A, Spruyt A, Sweep C, Corstens F, Smals A. Bone mineral density and quantitative ultrasound parameters in patients with Klinefelter’s syndrome after long-term testosterone substitution. *Osteoporosis International*. 2001;**12**:55-62. |
| A35 | Westlander G, Ekerhovd E, Granberg S, Hanson L, Hanson C, Bergh C. Testicular ultrasonography and extended chromosome analysis in men with nonmosaic Klinefelter syndrome: a prospective study of possible predictive factors for successful sperm recovery. *Fertility and Sterility*. 2001;**75**(6):1102-1105. |
| A36 | Keung Y-K, Buss D, Chauvenet A, Pettenati M. Hematologic malignancies and Klinefelter syndrome: a chance association? *Cancer genetics and cytogenetics*. 2002;**139**(1):9-13. |
| A37 | Madgar I, Dor J, Weissenberg R, Raviv G, Menashe Y, Levron J. Prognostic value of the clinical and laboratory evaluation in patients with nonmosaic Klinefelter syndrome who are receiving assisted reproductive therapy. *Fertility and sterility*. 2002;**77**(6):1167-1169. |
| A38 | Oktenli C, Yesilova Z, Kocar IH, Musabak U, Ozata M, Inal A, Gül D, Sanisoglu Y. Study of autoimmunity in Klinefelter's syndrome and idiopathic hypogonadotropic hypogonadism. *Journal of clinical immunology*. 2002;**22**:137-143. |
| A39 | Raman JD, Schlegel PN. Aromatase inhibitors for male infertility. *The Journal of urology*. 2002;**167**(2):624-629. |
| A40 | Christiansen P, Andersson A-M, Skakkebaek N. Longitudinal studies of inhibin B levels in boys and young adults with Klinefelter syndrome. *J Clin Endocrinol Metab*. 2003;**88**(2):888-891. |
| A41 | Kamischke A, Baumgardt A, Horst J, Nieschlag E. Clinical and diagnostic features of patients with suspected Klinefelter syndrome. *Journal of andrology*. 2003;**24**(1):41-48. |
| A42 | Stepan JJ, Burckhardt P, Hána V. The effects of three-month intravenous ibandronate on bone mineral density and bone remodeling in Klinefelter's syndrome: the influence of vitamin D deficiency and hormonal status. *Bone*. 2003;**33**(4):589-596. |
| A43 | Bojesen A, Juul S, Birkebæk N, Gravholt CH. Increased mortality in Klinefelter syndrome. *J Clin Endocrinol Metab*. 2004;**89**(8):3830-3834. |
| A44 | Chiang C-M, Lin C-J, Lee L-M, Chen S-M. Outcome of Intracytoplasmic Injection of Sperm Obtained by Testicular Sperm Extraction from 14 Azoospermic Men Suffering from 47, XXY Non-mosaic Klinefelter's Syndrome. *Taiwanese Journal of Obstetrics and Gynecology*. 2004;**43**(2):88-96. |
| A45 | Okada H, Shirakawa T, Ishikawa T, Goda K, Fujisawa M, Kamidono S. Serum testosterone levels in patients with nonmosaic Klinefelter syndrome after testicular sperm extraction for intracytoplasmic sperm injection. *Fertility and sterility*. 2004;**82**(1):237-238. |
| A46 | Seo JT, Park YS, Lee JS. Successful testicular sperm extraction in Korean Klinefelter syndrome. *Urology*. 2004;**64**(6):1208-1211. |
| A47 | Wielgos M, Bablok L, Fracki S, Czaplicki M, Marianowski L. The naloxone test in Klinefelter syndrome. *Neuroendocrinology Letters*. 2004;**25**(6) |
| A48 | Yesilova Z, Ozata M, Oktenli C, Sanisoglu SY, Erbil MK, Dagalp K. Effect of supraphysiologic doses of testosterone on fasting plasma total homocysteine concentrations in men with Klinefelter's syndrome. *Fertility and sterility*. 2004;**81**(5):1278-1282. |
| A49 | Okada H, Goda K, Yamamoto Y, Sofikitis N, Miyagawa I, Mio Y, Koshida M, Horie S. Age as a limiting factor for successful sperm retrieval in patients with nonmosaic Klinefelter’s syndrome. *Fertility and sterility*. 2005;**84**(6):1662-1664. |
| A50 | Schiff JD, Palermo GD, Veeck LL, Goldstein M, Rosenwaks Z, Schlegel PN. Success of testicular sperm injection and intracytoplasmic sperm injection in men with Klinefelter syndrome. *J Clin Endocrinol Metab*. 2005;**90**(11):6263-6267. |
| A51 | Swerdlow AJ, Higgins CD, Schoemaker MJ, Wright AF, Jacobs PA, Group UKCC. Mortality in patients with Klinefelter syndrome in Britain: a cohort study. *J Clin Endocrinol Metab*. 2005;**90**(12):6516-6522. |
| A52 | Swerdlow AJ, Schoemaker MJ, Higgins CD, Wright AF, Jacobs PA. Cancer incidence and mortality in men with Klinefelter syndrome: a cohort study. *Journal of the National Cancer Institute*. 2005;**97**(16):1204-1210. |
| A53 | Yesilova Z, Oktenli C, Sanisoglu SY, Musabak U, Cakir E, Ozata M, Dagalp K. Evaluation of insulin sensitivity in patients with Klinefelter’s syndrome: a hyperinsulinemic euglycemic clamp study. *Endocrine*. 2005;**27**:11-15. |
| A54 | Bakircioglu ME, Erden HF, Kaplancan T, Ciray N, Bener F, Bahceci M. Aging may adversely affect testicular sperm recovery in patients with Klinefelter syndrome. *Urology*. 2006;**68**(5):1082-1086. |
| A55 | Bojesen A, Juul S, Birkebæk NH, Gravholt CH. Morbidity in Klinefelter syndrome: a Danish register study based on hospital discharge diagnoses. *J Clin Endocrinol Metab*. 2006;**91**(4):1254-1260. |
| A56 | Bojesen A, Kristensen K, Birkebaek NH, Fedder J, Mosekilde L, Bennett P, Laurberg P, Frystyk J, Flyvbjerg A, Christiansen JS. The metabolic syndrome is frequent in Klinefelter’s syndrome and is associated with abdominal obesity and hypogonadism. *Diabetes care*. 2006;**29**(7):1591-1598. |
| A57 | Van Rijn S, Swaab H, Aleman A, Kahn RS. X Chromosomal effects on social cognitive processing and emotion regulation: A study with Klinefelter men (47, XXY). *Schizophrenia research*. 2006;**84**(2-3):194-203. |
| A58 | Koga M, Tsujimura A, Takeyama M, Kiuchi H, Takao T, Miyagawa Y, Takada S, Matsumiya K, Fujioka H, Okamoto Y. Clinical comparison of successful and failed microdissection testicular sperm extraction in patients with nonmosaic Klinefelter syndrome. *Urology*. 2007;**70**(2):341-345. |
| A59 | Lähdesmäki R, Alvesalo L. Root lengths in the permanent teeth of Klinefelter (47, XXY) men. *Archives of oral biology*. 2007;**52**(9):822-827. |
| A60 | Seo JT, Lee JS, Oh TH, Joo KJ. The clinical significance of bone mineral density and testosterone levels in Korean men with non‐mosaic Klinefelter’s syndrome. *BJU international*. 2007;**99**(1):141-146. |
| A61 | Ishikawa T, Yamaguchi K, Kondo Y, Takenaka A, Fujisawa M. Metabolic Syndrome in Men with Klinefelter's Syndrome. *Urology*. 2008/06/01/ 2008;**71**(6):1109-1113. doi:https://doi.org/10.1016/j.urology.2008.01.051 |
| A62 | Takada S, Tsujimura A, Ueda T, Matsuoka Y, Takao T, Miyagawa Y, Koga M, Takeyama M, Okamoto Y, Matsumiya K. Androgen decline in patients with nonobstructive azoospemia after microdissection testicular sperm extraction. *Urology*. 2008;**72**(1):114-118. |
| A63 | van Rijn S, Swaab H, Aleman A, Kahn RS. Social behavior and autism traits in a sex chromosomal disorder: Klinefelter (47XXY) syndrome. *Journal of autism and developmental disorders*. 2008;**38**:1634-1641. |
| A64 | van Rijn S, Aleman A, Swaab H, Vink M, Sommer I, Kahn RS. Effects of an extra X chromosome on language lateralization: an fMRI study with Klinefelter men (47, XXY). *Schizophrenia research*. 2008;**101**(1-3):17-25. |
| A65 | de Ronde W, de Haan A, Drent ML. Quality of life is reduced in patients with Klinefelter syndrome on androgen replacement therapy. *European journal of endocrinology*. 2009;**160**(3):465-468. |
| A66 | Ferhi K, Avakian R, Griveau JF, Guille F. Age as only predictive factor for successful sperm recovery in patients with Klinefelter’s syndrome. *Andrologia*. 2009;**41**(2):84-87. |
| A67 | Ramasamy R, Ricci JA, Palermo GD, Gosden LV, Rosenwaks Z, Schlegel PN. Successful fertility treatment for Klinefelter's syndrome. *The Journal of urology*. 2009;**182**(3):1108-1113. |
| A68 | van Rijn S, Aleman A, De Sonneville L, Swaab H. Cognitive mechanisms underlying disorganization of thought in a genetic syndrome (47, XXY). *Schizophrenia research*. 2009;**112**(1-3):91-98. |
| A69 | Yarali H, Polat M, Bozdag G, Gunel M, Alpas I, Esinler I, Dogan U, Tiras B. TESE–ICSI in patients with non-mosaic Klinefelter syndrome: a comparative study. *Reproductive biomedicine online*. 2009;**18**(6):756-760. |
| A70 | Bruining H, Van Rijn S, Swaab H, Giltay J, Kates W, Kas MJ, Van Engeland H, De Sonneville L. The parent-of-origin of the extra X chromosome may differentially affect psychopathology in Klinefelter syndrome. *Biological psychiatry*. 2010;**68**(12):1156-1162. |
| A71 | Corona G, Monami M, Boddi V, Cameron-Smith M, Fisher AD, de Vita G, Melani C, Balzi D, Sforza A, Forti G, Mannucci E, Maggi M. Low testosterone is associated with an increased risk of MACE lethality in subjects with erectile dysfunction. *Journal of Sexual Medicine*. Apr 2010;**7**(4 Pt 1):1557-64. doi:10.1111/j.1743-6109.2009.01690.x |
| A72 | Aksglæde L, Skakkebæk NE, Almstrup K, Juul A. Clinical and biological parameters in 166 boys, adolescents and adults with nonmosaic Klinefelter syndrome: a Copenhagen experience. *Acta paediatrica*. 2011;**100**(6):793-806. |
| A73 | Bakircioglu ME, Ulug U, Erden HF, Tosun S, Bayram A, Ciray N, Bahceci M. Klinefelter syndrome: does it confer a bad prognosis in treatment of nonobstructive azoospermia? *Fertility and sterility*. 2011;**95**(5):1696-1699. |
| A74 | Bojesen A, Stochholm K, Juul S, Gravholt CH. Socioeconomic Trajectories Affect Mortality in Klinefelter Syndrome. *J Clin Endocrinol Metab*. 2011;**96**(7):2098-2104. doi:10.1210/jc.2011-0367 |
| A75 | Herlihy AS, McLachlan RI, Gillam L, Cock ML, Collins V, Halliday JL. The psychosocial impact of Klinefelter syndrome and factors influencing quality of life. *Genetics in Medicine*. 2011;**13**(7):632-642. |
| A76 | Kompus K, Westerhausen R, Nilsson L-G, Hugdahl K, Jongstra S, Berglund A, Arver S, Savic I. Deficits in inhibitory executive functions in Klinefelter (47, XXY) syndrome. *Psychiatry Research*. 2011;**189**(1):135-140. |
| A77 | Melhem N, Middleton F, McFadden K, Klei L, Faraone SV, Vinogradov S, Tiobech J, Yano V, Kuartei S, Roeder K. Copy number variants for schizophrenia and related psychotic disorders in Oceanic Palau: risk and transmission in extended pedigrees. *Biological psychiatry*. 2011;**70**(12):1115-1121. |
| A78 | Trabado S, Maione L, Salenave S, Baron S, Galland F, Bry-Gauillard H, Guiochon-Mantel A, Chanson P, Pitteloud N, Sinisi AA. Estradiol levels in men with congenital hypogonadotropic hypogonadism and the effects of different modalities of hormonal treatment. *Fertility and sterility*. 2011;**95**(7):2324-2329. e3. |
| A79 | Turriff A, Levy HP, Biesecker B. Prevalence and psychosocial correlates of depressive symptoms among adolescents and adults with Klinefelter syndrome. *Genetics in Medicine*. 2011;**13**(11):966-972. |
| A80 | van Rijn S, Swaab H. Vulnerability for psychopathology in Klinefelter syndrome: age‐specific and cognitive‐specific risk profiles. *Acta Paediatrica*. 2011;**100**(6):908-916. |
| A81 | Bak CW, Byun JS, Lee JH, Park JH, Lee KA, Shim SH. Clinical and social characteristics of Korean men with Klinefelter syndrome. *International journal of urology*. 2012;**19**(5):443-449. |
| A82 | Dillon SP, Kurien BT, Li S, Bruner GR, Kaufman KM, Harley JB, Gaffney PM, Wallace DJ, Weisman MH, Scofield RH. Sex chromosome aneuploidies among men with systemic lupus erythematosus. *Journal of autoimmunity*. 2012;**38**(2-3):J129-J134. |
| A83 | Foresta C, Caretta N, Palego P, Ferlin A, Zuccarello D, Lenzi A, Selice R. Reduced artery diameters in Klinefelter syndrome. *International Journal of Andrology*. 2012;**35**(5):720-725. |
| A84 | Jiang-Feng M, Hong-Li X, Xue-Yan W, Min N, Shuang-Yu L, Hong-Ding X, Liang-Ming L. Prevalence and risk factors of diabetes in patients with Klinefelter syndrome: a longitudinal observational study. *Fertility and sterility*. 2012;**98**(5):1331-1335. |
| A85 | Kota SK, Meher LK, Jammula S, Kota SK, Modi KD. Clinical profile of coexisting conditions in type 1 diabetes mellitus patients. *Diabetes & Metabolic Syndrome: Clinical Research & Reviews*. 2012;**6**(2):70-76. |
| A86 | Pacenza N, Pasqualini T, Gottlieb S, et al. Clinical Presentation of Klinefelter's Syndrome: Differences According to Age. Int J Endocrinol. 2012;2012:324835. doi:10.1155/2012/324835 |
| A87 | van Rijn S, Bierman M, Bruining H, Swaab H. Vulnerability for autism traits in boys and men with an extra X chromosome (47, XXY): the mediating role of cognitive flexibility. *Journal of psychiatric research*. 2012;**46**(10):1300-1306. |
| A88 | van Saen D, Tournaye H, Goossens E. Presence of spermatogonia in 47, XXY men with no spermatozoa recovered after testicular sperm extraction. *Fertility and sterility*. 2012;**97**(2):319-323. |
| A89 | Ando M, Yamaguchi K, Chiba K, Miyake H, Fujisawa M. Outcome of microdissection testicular sperm extraction in azoospermic patients with Klinefelter syndrome and other sex-chromosomal anomalies. *Systems Biology in Reproductive Medicine*. 2013;**59**(4):210-213. |
| A90 | Inci M, Akgul O, Baydilli N, Ekmekcioglu O, Özgöçmen S. Increased femoral cartilage thickness in patients with Klinefelter syndrome. *American Journal of Men's Health*. 2013;**7**(1):54-57. |
| A91 | Jo DG, Lee HS, Joo YM, Seo JT. Effect of testosterone replacement therapy on bone mineral density in patients with Klinefelter syndrome. *Yonsei medical journal*. 2013;**54**(6):1331-1335. |
| A92 | Mehta A, Bolyakov A, Roosma J, Schlegel PN, Paduch DA. Successful testicular sperm retrieval in adolescents with Klinefelter syndrome treated with at least 1 year of topical testosterone and aromatase inhibitor. *Fertility and sterility*. 2013;**100**(4):970-974. |
| A93 | Pasquali D, Arcopinto M, Renzullo A, Rotondi M, Accardo G, Salzano A, Esposito D, Saldamarco L, Isidori AM, Marra AM. Cardiovascular abnormalities in Klinefelter syndrome. *International journal of cardiology*. 2013;**168**(2):754-759. |
| A94 | Selice R, Caretta N, Di Mambro A, Torino M, Palego P, Ferlin A, Foresta C. Prostate volume and growth during testosterone replacement therapy is related to visceral obesity in Klinefelter syndrome. *European journal of endocrinology*. 2013;**169**(6):743-749. |
| A95 | Brinton LA, Cook MB, McCormack V, Johnson KC, Olsson H, Casagrande JT, Cooke R, Falk RT, Gapstur SM, Gaudet MM. Anthropometric and hormonal risk factors for male breast cancer: male breast cancer pooling project results. *Journal of the National Cancer Institute*. 2014;**106**(3):djt465. |
| A96 | Bryson CF, Ramasamy R, Sheehan M, Palermo GD, Rosenwaks Z, Schlegel PN. Severe testicular atrophy does not affect the success of microdissection testicular sperm extraction. *The Journal of urology*. 2014;**191**(1):175-178. |
| A97 | Cederlöf M, Gotby AO, Larsson H, Serlachius E, Boman M, Långström N, Landén M, Lichtenstein P. Klinefelter syndrome and risk of psychosis, autism and ADHD. *Journal of Psychiatric Research*. 2014;**48**(1):128-130. |
| A98 | Overvad S, Bay K, Bojesen A, Gravholt C. Low INSL3 in Klinefelter syndrome is related to osteocalcin, testosterone treatment and body composition, as well as measures of the hypothalamic–pituitary–gonadal axis. *Andrology*. 2014;**2**(3):421-427. |
| A99 | Sabbaghian M, Modarresi T, Hosseinifar H, Hosseini J, Farrahi F, Dadkhah F, Chehrazi M, Khalili G, Gilani MAS. Comparison of sperm retrieval and intracytoplasmic sperm injection outcome in patients with and without Klinefelter syndrome. *Urology*. 2014;**83**(1):107-110. |
| A100 | Samplaski MK, Lo KC, Grober ED, Millar A, Dimitromanolakis A, Jarvi KA. Phenotypic differences in mosaic Klinefelter patients as compared with non-mosaic Klinefelter patients. *Fertility and sterility*. 2014;**101**(4):950-955. |
| A101 | Shanbhogue VV, Hansen S, Jørgensen NR, Brixen K, Gravholt CH. Bone geometry, volumetric density, microarchitecture, and estimated bone strength assessed by HR‐pQCT in Klinefelter syndrome. *Journal of Bone and Mineral Research*. 2014;**29**(11):2474-2482. |
| A102 | Skakkebæk A, Bojesen A, Kristensen M, Cohen A, Hougaard D, Hertz JM, Fedder J, Laurberg P, Wallentin M, Østergaard J. Neuropsychology and brain morphology in Klinefelter syndrome–the impact of genetics. *Andrology*. 2014;**2**(4):632-640. |
| A103 | Skakkebæk A, Gravholt CH, Rasmussen PM, Bojesen A, Jensen JS, Fedder J, Laurberg P, Hertz JM, Østergaard JR, Pedersen AD. Neuroanatomical correlates of Klinefelter syndrome studied in relation to the neuropsychological profile. *NeuroImage: Clinical*. 2014;**4**:1-9. |
| A104 | Chang S, Skakkebæk A, Trolle C, Bojesen A, Hertz JM, Cohen A, Hougaard DM, Wallentin M, Pedersen AD, Østergaard JR. Anthropometry in Klinefelter syndrome-multifactorial influences due to CAG length, testosterone treatment and possibly intrauterine hypogonadism. *J Clin Endocrinol Metab*. 2015;**100**(3):E508-E517. |
| A105 | Fedder J, Gravholt CH, Kristensen SG, Marcussen N, Engvad B, Milton AM, Andersen CY. Testicular sperm sampling by subcapsular orchiectomy in Klinefelter patients: a new simplified treatment approach. *Urology*. 2015;**86**(4):744-750. |
| A106 | Ferlin A, Selice R, Di Mambro A, Ghezzi M, Di Nisio A, Caretta N, Foresta C. Role of vitamin D levels and vitamin D supplementation on bone mineral density in Klinefelter syndrome. *Osteoporosis International*. 2015;**26**:2193-2202. |
| A107 | Fisher AD, Castellini G, Casale H, Fanni E, Bandini E, Campone B, Ferruccio N, Maseroli E, Boddi V, Dèttore D. Hypersexuality, paraphilic behaviors, and gender dysphoria in individuals with Klinefelter’s syndrome. *The Journal of Sexual Medicine*. 2015;**12**(12):2413-2424. |
| A108 | Giagulli VA, Carbone MD, Ramunni MI, Licchelli B, De Pergola G, Sabbà C, Guastamacchia E, Triggiani V. Adding liraglutide to lifestyle changes, metformin and testosterone therapy boosts erectile function in diabetic obese men with overt hypogonadism. *Andrology*. 2015/11/01 2015;**3**(6):1094-1103. doi:https://doi.org/10.1111/andr.12099 |
| A109 | Gudeman SR, Townsend B, Fischer K, Walters RC, Crain D. Etiology of azoospermia in a military population. *The Journal of urology*. 2015;**193**(4):1318-1321. |
| A110 | Jaeger G, Röjvik A, Berglund B. Participation in society for people with a rare diagnosis. *Disability and Health Journal*. 2015;**8**(1):44-50. |
| A111 | Jørgensen IN, Skakkebaek A, Andersen NH, Pedersen LN, Hougaard DM, Bojesen A, Trolle C, Gravholt CH. Short QTc interval in males with klinefelter syndrome—Influence of CAG repeat length, body composition, and testosterone replacement therapy. *Pacing and Clinical Electrophysiology*. 2015;**38**(4):472-482. |
| A112 | Plotton I, d'Estaing SG, Cuzin B, Brosse A, Benchaib M, Lornage J, Ecochard R, Dijoud F, Lejeune H, group F. Preliminary results of a prospective study of testicular sperm extraction in young versus adult patients with nonmosaic 47, XXY Klinefelter syndrome. *J Clin Endocrinol Metab*. 2015;**100**(3):961-967. |
| A113 | Rohayem J, Fricke R, Czeloth K, Mallidis C, Wistuba J, Krallmann C, Zitzmann M, Kliesch S. Age and markers of Leydig cell function, but not of Sertoli cell function predict the success of sperm retrieval in adolescents and adults with Klinefelter's syndrome. *Andrology*. 2015;**3**(5):868-875. |
| A114 | Turriff A, Levy HP, Biesecker B. Factors associated with adaptation to Klinefelter syndrome: the experience of adolescents and adults. *Patient education and counseling*. 2015;**98**(1):90-95. |
| A115 | Belli S, Santi D, Leoni E, Dall'Olio E, Fanelli F, Mezzullo M, Pelusi C, Roli L, Tagliavini S, Trenti T. Human chorionic gonadotropin stimulation gives evidence of differences in testicular steroidogenesis in Klinefelter syndrome, as assessed by liquid chromatography–tandem mass spectrometry. *European Journal of Endocrinology*. 2016;**174**(6):801-811. |
| A116 | Harris VM, Sharma R, Cavett J, Kurien BT, Liu K, Koelsch KA, Rasmussen A, Radfar L, Lewis D, Stone DU. Klinefelter's syndrome (47, XXY) is in excess among men with Sjögren's syndrome. *Clinical Immunology*. 2016;**168**:25-29. |
| A117 | Majzoub A, Arafa M, Al Said S, Agarwal A, Seif A, Al Naimi A, El Bardisi H. Outcome of testicular sperm extraction in nonmosaic Klinefelter syndrome patients: what is the best approach? *Andrologia*. 2016;**48**(2):171-176. |
| A118 | Nahata L, Richard NY, Paltiel HJ, Chow JS, Logvinenko T, Rosoklija I, Cohen LE. Sperm retrieval in adolescents and young adults with Klinefelter syndrome: a prospective, pilot study. *The Journal of pediatrics*. 2016;**170**:260-265. e2. |
| A119 | Rohayem J, Nieschlag E, Zitzmann M, Kliesch S. Testicular function during puberty and young adulthood in patients with Klinefelter's syndrome with and without spermatozoa in seminal fluid. *Andrology*. 2016;**4**(6):1178-1186. |
| A120 | Wallentin M, Skakkebæk A, Bojesen A, Fedder J, Laurberg P, Østergaard JR, Hertz JM, Pedersen AD, Gravholt CH. Klinefelter syndrome has increased brain responses to auditory stimuli and motor output, but not to visual stimuli or Stroop adaptation. *NeuroImage: Clinical*. 2016;**11**:239-251. |
| A121 | Belling K, Russo F, Jensen AB, Dalgaard MD, Westergaard D, Rajpert-De Meyts E, Skakkebæk NE, Juul A, Brunak S. Klinefelter syndrome comorbidities linked to increased X chromosome gene dosage and altered protein interactome activity. *Human Molecular Genetics*. 2017;**26**(7):1219-1229. |
| A122 | Binsaleh S, Alhajeri D, Madbouly K. Microdissection testicular sperm extraction in men with nonobstructive azoospermia: experience of King Saud University Medical City, Riyadh, Saudi Arabia. *Urology Annals*. 2017;**9**(2):136. |
| A123 | Chehrazi M, Rahimiforoushani A, Sabbaghian M, Nourijelyani K, Gilani MAS, Hoseini M, Vesali S, Yaseri M, Alizadeh A, Mohammad K. Sperm retrieval in patients with Klinefelter syndrome: a skewed regression model analysis. *International Journal of Fertility & Sterility*. 2017;**11**(2):117. |
| A124 | Corona G, Pizzocaro A, Lanfranco F, Garolla A, Pelliccione F, Vignozzi L, Ferlin A, Foresta C, Jannini EA, Maggi M. Sperm recovery and ICSI outcomes in Klinefelter syndrome: a systematic review and meta-analysis. *Human Reproduction Update*. 2017;**23**(3):265-275. |
| A125 | El Bardisi H, Majzoub A, Al Said S, Alnawasra H, Dabbous Z, Arafa M. Sexual dysfunction in Klinefelter's syndrome patients. *Andrologia*. 2017;**49**(6):e12670. |
| A126 | Iwatsuki S, Sasaki S, Taguchi K, Hamakawa T, Mizuno K, Okada A, Kubota Y, Umemoto Y, Hayashi Y, Yasui T. Effect of obesity on sperm retrieval outcome and reproductive hormone levels in Japanese azoospermic men with and without Klinefelter syndrome. *Andrology*. 2017;**5**(1):82-86. |
| A127 | Ku M-H, Huang I-S, Lin AT, Chen K-K, Huang WJ. The predictive value of parameters of clinical presentations for sperm yield in patients with nonobstructive azoospermia receiving microdissection testicular sperm extraction. *Urological science*. 2017;**28**(4):243-247. |
| A128 | Lee HS, Park CW, Lee JS, Seo JT. Hypogonadism makes dyslipidemia in Klinefelter's syndrome. *Journal of Korean Medical Science*. 2017;**32**(11):1848-1851. |
| A129 | Liberato D, Granato S, Grimaldi D, Rossi F, Tahani N, Gianfrilli D, Anzuini A, Lenzi A, Cavaggioni G, Radicioni A. Fluid intelligence, traits of personality and personality disorders in a cohort of adult KS patients with the classic 47, XXY karyotype. *Journal of Endocrinological Investigation*. 2017;**40**:1191-1199. |
| A130 | Olesen IA, Andersson A-M, Aksglaede L, Skakkebaek NE, Rajpert–de Meyts E, Joergensen N, Juul A. Clinical, genetic, biochemical, and testicular biopsy findings among 1,213 men evaluated for infertility. *Fertility and sterility*. 2017;**107**(1):74-82. e7. |
| A131 | Rocher L, Criton A, Gennisson J-L, Izard V, Ferlicot S, Tanter M, Benoit G, Bellin MF, Correas J-M. Testicular shear wave elastography in normal and infertile men: a prospective study on 601 patients. *Ultrasound in medicine & biology*. 2017;**43**(4):782-789. |
| A132 | Skakkebæk A, Moore PJ, Pedersen AD, Bojesen A, Kristensen MK, Fedder J, Laurberg P, Hertz JM, Østergaard JR, Wallentin M. The role of genes, intelligence, personality, and social engagement in cognitive performance in Klinefelter syndrome. *Brain and Behavior*. 2017;**7**(3):e00645. |
| A133 | Ali M, Soliman M, Metwally A, Ghobeish A. Cytogenetic analysis and endocrine profile in patients with nonobstructive azoospermia or severe oligozoospermia. *African Journal of Urology*. 2018;**24**(3):212-215. |
| A134 | Eken A, Gulec F. Microdissection testicular sperm extraction (micro-TESE): predictive value of preoperative hormonal levels and pathology in non-obstructive azoospermia. *The Kaohsiung journal of medical sciences*. 2018;**34**(2):103-108. |
| A135 | Eliveld J, van Wely M, Meissner A, Repping S, van der Veen F, van Pelt AM. The risk of TESE-induced hypogonadism: a systematic review and meta-analysis. *Human reproduction update*. 2018;**24**(4):442-454. |
| A136 | Ferlin A, Selice R, Angelini S, Di Grazia M, Caretta N, Cavalieri F, Di Mambro A, Foresta C. Endocrine and psychological aspects of sexual dysfunction in Klinefelter patients. *Andrology*. 2018;**6**(3):414-419. |
| A137 | Garolla A, Selice R, Menegazzo M, Valente U, Zattoni F, Iafrate M, Prayer‐Galetti T, Gardiman MP, Ferlin A, Di Nisio A. Novel insights on testicular volume and testosterone replacement therapy in Klinefelter patients undergoing testicular sperm extraction. A retrospective clinical study. *Clinical Endocrinology*. 2018;**88**(5):711-718. |
| A138 | Kreukels BP, Köhler B, Nordenström A, Roehle R, Thyen U, Bouvattier C, de Vries AL, Cohen-Kettenis PT, group d-L. Gender dysphoria and gender change in disorders of sex development/intersex conditions: results from the dsd-LIFE study. *The Journal of Sexual Medicine*. 2018;**15**(5):777-785. |
| A139 | Nordenström A, Röhle R, Thyen U, Bouvattier C, Slowikowska‐Hilczer J, Reisch N, Claahsen van der Grinten H, Brac de la Perriere A, Cohen‐Kettenis PT, Köhler B. Hormone therapy and patient satisfaction with treatment, in a large cohort of diverse disorders of sex development. *Clinical endocrinology*. 2018;**88**(3):397-408. |
| A140 | Rapp M, Mueller-Godeffroy E, Lee P, Roehle R, Kreukels BP, Köhler B, Nordenström A, Bouvattier C, Thyen U. Multicentre cross-sectional clinical evaluation study about quality of life in adults with disorders/differences of sex development (DSD) compared to country specific reference populations (dsd-LIFE). *Health and Quality of Life Outcomes*. 2018;**16**(1):1-13. |
| A141 | Skakkebæk A, Moore PJ, Chang S, Fedder J, Gravholt CH. Quality of life in men with Klinefelter syndrome: the impact of genotype, health, socioeconomics, and sexual function. *Genetics in Medicine*. 2018;**20**(2):214-222. |
| A142 | Skakkebæk A, Moore PJ, Pedersen AD, Bojesen A, Kristensen MK, Fedder J, Hertz JM, Østergaard JR, Wallentin M, Gravholt CH. Anxiety and depression in Klinefelter syndrome: the impact of personality and social engagement. *PLoS One*. 2018;**13**(11):e0206932. |
| A143 | Thyen U, Ittermann T, Flessa S, Muehlan H, Birnbaum W, Rapp M, Marshall L, Szarras-Capnik M, Bouvattier C, Kreukels BP. Quality of health care in adolescents and adults with disorders/differences of sex development (DSD) in six European countries (dsd-LIFE). *BMC Health Services Research*. 2018;**18**:1-12. |
| A144 | van de Grift TC, Cohen-Kettenis PT, de Vries AL, Kreukels BP. Body image and self-esteem in disorders of sex development: A European multicenter study. *Health Psychology*. 2018;**37**(4):334. |
| A145 | van Rijn S, de Sonneville L, Swaab H. The nature of social cognitive deficits in children and adults with Klinefelter syndrome (47, XXY). *Genes, Brain and Behavior*. 2018;**17**(6):e12465. |
| A146 | van Saen D, Vloeberghs V, Gies I, Mateizel I, Sermon K, De Schepper J, Tournaye H, Goossens E. When does germ cell loss and fibrosis occur in patients with Klinefelter syndrome? *Human Reproduction*. 2018;**33**(6):1009-1022. |
| A147 | Balercia G, Bonomi M, Giagulli V, Lanfranco F, Rochira V, Giambersio A, Accardo G, Esposito D, Allasia S, Cangiano B. Thyroid function in Klinefelter syndrome: a multicentre study from KING group. *Journal of endocrinological investigation*. 2019;**42**:1199-1204. |
| A148 | Chang S, Biltoft D, Skakkebæk A, Fedder J, Bojesen A, Bor MV, Gravholt CH, Münster A-MB. Testosterone treatment and association with thrombin generation and coagulation inhibition in Klinefelter syndrome: A cross-sectional study. *Thrombosis Research*. 2019/10/01/ 2019;**182**:175-181. doi:https://doi.org/10.1016/j.thromres.2019.08.011 |
| A149 | De Vries AL, Roehle R, Marshall L, Frisén L, Van De Grift TC, Kreukels BP, Bouvattier C, Köhler B, Thyen U, Nordenström A. Mental health of a large group of adults with disorders of sex development in six European countries. *Psychosomatic medicine*. 2019;**81**(7):629. |
| A150 | Granato S, Barbaro G, Di Giorgio MR, Rossi FM, Marzano C, Impronta F, Spaziani M, Anzuini A, Lenzi A, Radicioni AF. Epicardial fat: the role of testosterone and lipid metabolism in a cohort of patients with Klinefelter syndrome. *Metabolism*. 2019/06/01/ 2019;**95**:21-26. doi:https://doi.org/10.1016/j.metabol.2019.03.002 |
| A151 | Høst C, Bojesen A, Erlandsen M, Groth KA, Kristensen K, Jurik AG, Birkebæk NH, Gravholt CH. A placebo-controlled randomized study with testosterone in Klinefelter syndrome: beneficial effects on body composition. *Endocrine Connections*. 2019;**8**(9):1250-1261. |
| A152 | Mortensen LJ, Lorenzen M, Jørgensen N, Andersson A-M, Nielsen JE, Petersen LI, Lanske B, Juul A, Hansen JB, Jensen MB. Possible link between FSH and RANKL release from adipocytes in men with impaired gonadal function including Klinefelter syndrome. *Bone*. 2019;**123**:103-114. |
| A153 | Santi D, De Vincentis S, Scaltriti S, Rochira V. Relative hyperestrogenism in Klinefelter Syndrome: results from a meta-analysis. *Endocrine*. 2019;**64**:209-219. |
| A154 | Deebel NA, Galdon G, Zarandi NP, Stogner-Underwood K, Howards S, Lovato J, Kogan S, Atala A, Lue Y, Sadri-Ardekani H. Age-related presence of spermatogonia in patients with Klinefelter syndrome: a systematic review and meta-analysis. *Human Reproduction Update*. 2020;**26**(1):58-72. |
| A155 | Guo F, Fang A, Fan Y, Fu X, Lan Y, Liu M, Cao S, An G. Role of treatment with human chorionic gonadotropin and clinical parameters on testicular sperm recovery with microdissection testicular sperm extraction and intracytoplasmic sperm injection outcomes in 184 Klinefelter syndrome patients. *Fertility and Sterility*. 2020;**114**(5):997-1005. |
| A156 | Hussein TM, Abd Elmoaty Elneily D, Mohamed Abdelfattah Elsayed F, El-Attar LM. Genetic risk factors for venous thromboembolism among infertile men with Klinefelter syndrome. *Journal of Clinical & Translational Endocrinology*. 2020/06/01/ 2020;**20**:100228. doi:https://doi.org/10.1016/j.jcte.2020.100228 |
| A157 | Madian A, Eid MM, Shahin AA, Mazen I, El-Bassyouni HT, Eid OM. Detection of low-grade mosaicism and its correlation with hormonal profile, testicular volume, and semen quality in a cohort of Egyptian Klinefelter and Klinefelter-like patients. *Reproductive Biology*. 2020;**20**(2):259-263. |
| A158 | Pizzocaro A, Vena W, Condorelli R, Radicioni A, Rastrelli G, Pasquali D, Selice R, Ferlin A, Foresta C, Jannini E. Testosterone treatment in male patients with Klinefelter syndrome: a systematic review and meta-analysis. *Journal of Endocrinological Investigation*. 2020;**43**:1675-1687. |
| A159 | Pozzi E, Boeri L, Capogrosso P, Palmisano F, Preto M, Sibona M, Franceschelli A, Ruiz‐Castañé E, Sarquella‐Geli J, Bassas‐Arnau L. Rates of hypogonadism forms in Klinefelter patients undergoing testicular sperm extraction: A multicenter cross‐sectional study. *Andrology*. 2020;**8**(6):1705-1711. |
| A160 | Slowikowska-Hilczer J, Szarras-Czapnik M, Duranteau L, Rapp M, Walczak-Jedrzejowska R, Marchlewska K, Oszukowska E, Nordenstrom A. Risk of gonadal neoplasia in patients with disorders/differences of sex development. *Cancer epidemiology*. 2020;**69**:101800. |
| A161 | van Saen D, Vloeberghs V, Gies I, De Schepper J, Tournaye H, Goossens E. Characterization of the stem cell niche components within the seminiferous tubules in testicular biopsies of Klinefelter patients. *Fertility and sterility*. 2020;**113**(6):1183-1195. e3. |
| A162 | Vena W, Pizzocaro A, Indirli R, Amer M, Maffezzoni F, Delbarba A, Leonardi L, Balzarini L, Ulivieri FM, Ferlin A. Prevalence and determinants of radiological vertebral fractures in patients with Klinefelter syndrome. *Andrology*. 2020;**8**(6):1699-1704. |
| A163 | Barbonetti A, D’Andrea S, Vena W, Pizzocaro A, Rastrelli G, Pallotti F, Condorelli R, Calogero AE, Pasquali D, Ferlin A. Erectile dysfunction and decreased libido in Klinefelter syndrome: a prevalence meta-analysis and meta-regression study. *The journal of sexual medicine*. 2021;**18**(6):1053-1064. |
| A164 | Fabrazzo M, Accardo G, Abbondandolo I, Goglia G, Esposito D, Sampogna G, Catapano F, Giugliano D, Pasquali D. Quality of life in Klinefelter patients on testosterone replacement therapy compared to healthy controls: an observational study on the impact of psychological distress, personality traits, and coping strategies. *Journal of Endocrinological Investigation*. 2021;**44**:1053-1063. |
| A165 | Indirli R, Ferrante E, Scalambrino E, Profka E, Clerici M, Lettera T, Serban AL, Vena W, Pizzocaro A, Bonomi M. Procoagulant imbalance in Klinefelter syndrome assessed by thrombin generation assay and whole-blood thromboelastometry. *J Clin Endocrinol Metab*. 2021;**106**(4):1660-1672. |
| A166 | Özman O, Tosun S, Bayazıt N, Cengiz S, Bakırcıoğlu ME. Efficacy of the second micro–testicular sperm extraction after failed first micro–testicular sperm extraction in men with nonobstructive azoospermia. *Fertility and Sterility*. 2021;**115**(4):915-921. |
| A167 | Rapp M, Duranteau L, van de Grift TC, Schober J, Hirschberg AL, Krege S, Nordenstrom A, Roehle R, Thyen U, Bouvattier C. Self-and proxy-reported outcomes after surgery in people with disorders/differences of sex development (DSD) in Europe (dsd-LIFE). *Journal of pediatric urology*. 2021;**17**(3):353-365. |
| A168 | Chu KY, Ory J, Punjani N, Nassau DE, Israeli J, Kashanian JA, Ramasamy R. Utility of evaluating semen samples from adolescents with Klinefelter Syndrome for cryopreservation: A multi-institution evaluation. *Journal of pediatric urology*. 2022;**18**(3):288. e1-288. e5. |
| A169 | de Brouwer IJ, Suijkerbuijk M, van de Grift TC, Kreukels BP. First Adolescent Romantic and Sexual Experiences in Individuals with Differences of Sex Development/Intersex Conditions. *Journal of Adolescent Health*. 2022;**71**(6):688-695. |
| A170 | Deebel NA, Soltanghoraee H, Bradshaw AW, Abdelaal O, Reynolds K, Howards S, Kogan S, Sadeghi MR, Atala A, Stogner-Underwood K. Morphometric and immunohistochemical analysis as a method to identify undifferentiated spermatogonial cells in adult subjects with Klinefelter syndrome: a cohort study. *Fertility and Sterility*. 2022;**118**(5):864-873. |
| A171 | Giovanelli L, Quinton R, Cangiano B, Colombo S, Persani L, Bonomi M, Chiodini I. FSH and bone: Comparison between males with central versus primary hypogonadism. *Front Endocrinol (Lausanne)*. 2022;**13**:939897. |
| A172 | Kızılay F, Altay B, Aşçı R, Çayan S, Ekmekçioğlu O, Yaman Ö, Kadıoğlu A. Retrospective analysis of factors affecting sperm retrieval with microscopic testicular sperm extraction in infertile men with Klinefelter syndrome: A multicentre study. *Andrologia*. 2022;**54**(5):e14379. |
| A173 | Majzoub A, Arafa M, Clemens H, Imperial J, Leisegang K, Khalafalla K, Agarwal A, Henkel R, Elbardisi H. A systemic review and meta‐analysis exploring the predictors of sperm retrieval in patients with non‐obstructive azoospermia and chromosomal abnormalities. *Andrologia*. 2022;**54**(3):e14303. |
| A174 | Özkan B, Coşkuner ER, Güdelci T. Predictive factors and intracytoplasmic sperm injection results for sperm retrieval by microdissection testicular sperm extraction (micro-TESE) in patients with Klinefelter syndrome. *Urology*. 2022;**161**:59-64. |
| A175 | Pasquali D, Chiodini P, Simeon V, Ferlin A, Vignozzi L, Corona G, Lanfranco F, Rochira V, Calogero A, Bonomi M. First baseline data of the Klinefelter ItaliaN Group (KING) cohort: clinical features of adult with Klinefelter syndrome in Italy. *Journal of Endocrinological Investigation*. 2022;**45**(9):1769-1776. |
| A176 | Renault L, Labrune E, Giscard d’Estaing S, Cuzin B, Lapoirie M, Benchaib M, Lornage J, Soignon G, de Souza A, Dijoud F. Delaying testicular sperm extraction in 47, XXY Klinefelter patients does not impair the sperm retrieval rate, and AMH levels are higher when TESE is positive. *Human Reproduction*. 2022;**37**(11):2518-2531. |
| A177 | van de Grift TC, Rapp M, Holmdahl G, Duranteau L, Nordenskjold A, group dL, Kohler B, Neumann U, Cohen‐Kettenis P, Kreukels B. Masculinizing surgery in disorders/differences of sex development: clinician‐and participant‐evaluated appearance and function. *BJU international*. 2022;**129**(3):394-405. |
| A178 | Zhao Y, Gardner EJ, Tuke MA, Zhang H, Pietzner M, Koprulu M, Jia RY, Ruth KS, Wood AR, Beaumont RN, Tyrrell J, Jones SE, Lango Allen H, Day FR, Langenberg C, Frayling TM, Weedon MN, Perry JRB, Ong KK, Murray A. Detection and characterization of male sex chromosome abnormalities in the UK Biobank study. *Genetics in Medicine*. 2022/09/01/ 2022;**24**(9):1909-1919. doi:https://doi.org/10.1016/j.gim.2022.05.011 |
| A179 | Demirci I, Haymana C, Candemir B, Yuksel B, Eser M, Meric C, Akin S, Gulcelik NE, Sonmez A. Triglyceride-glucose index levels in patients with Klinefelter syndrome and its relationship with endothelial dysfunction and insulin resistance: a cross-sectional observational study. *Archives of Endocrinology and Metabolism*. 2023;**67**:378-384. |
| A180 | Eliveld J, van der Bles I, van Wely M, Meißner A, Soufan AT, Heijboer AC, Repping S, van der Veen F, van Pelt AM. The risk of hypogonadism after testicular sperm extraction in men with various types of azoospermia: a prospective cohort study. *Reproductive BioMedicine Online*. 2023;**46**(6):973-981. |
| A181 | Fjermestad K, Bergh N, Fleten S, Huster R, Gravholt C, Solbakk A-K. Mentalization and cognitive skills in men with Klinefelter syndrome versus non-clinical controls. *Psychiatry Research Communications*. 2023;**3**(2):100116. |
| A182 | Franik S, Fleischer K, Kortmann B, Stikkelbroeck NM, D’hauwers K, Bouvattier C, Slowikowska-Hilczer J, Grunenwald S, van de Grift T, Cartault A. Quality of life in men with Klinefelter syndrome: a multicentre study. *Endocrine Connections*. 2023;**12**(10) |
| A183 | Sánchez XC, Montalbano S, Vaez M, Krebs MD, Byberg-Grauholm J, Mortensen PB, Børglum AD, Hougaard DM, Nordentoft M, Geschwind DH. Associations of psychiatric disorders with sex chromosome aneuploidies in the Danish iPSYCH2015 dataset: a case-cohort study. *The Lancet Psychiatry*. 2023;**10**(2):129-138. |

**Supplementary Table 1: References of included studies.** This table provides a list of the full references of the included studies- P denotes a paediatric study and A studies in adults.

| **Ref No** | **Author** | **Year** | **Country of origin** | **Study design** | **Participant no** | **Mean age ± SD (years) or range** | **Bio** | **PA** | **Fe** | **CP** | **CM** | **QoL** | **Quality** |
| --- | --- | --- | --- | --- | --- | --- | --- | --- | --- | --- | --- | --- | --- |
| P1 | Ferguson-Smith 1959 | 1959 | UK | Cohort | 8 | 7-12 | 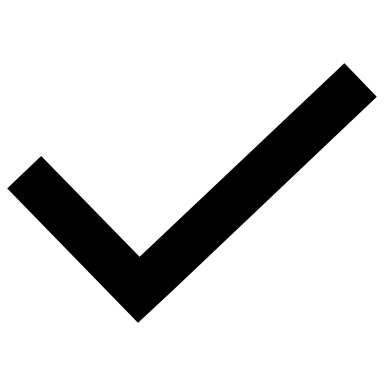 | 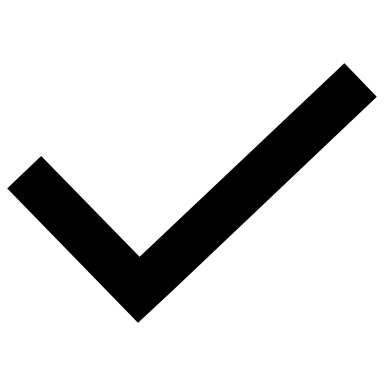 | 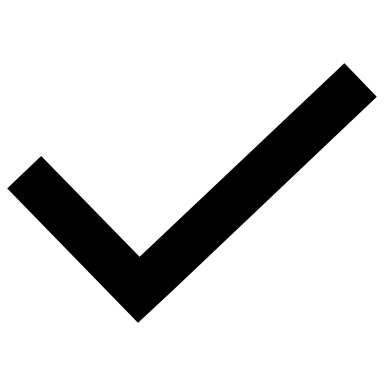 | 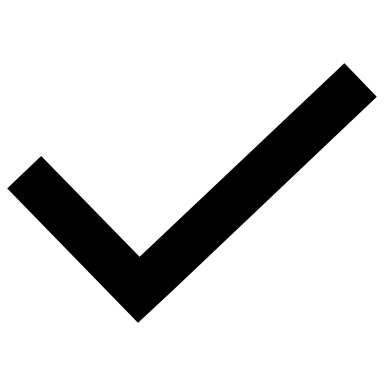 |  |  | Medium |
| P2 | Johnson 1970 | 1970 | United States | Case series | 5 | 9-24 |  | 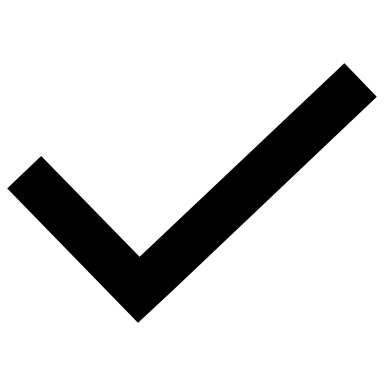 |  | 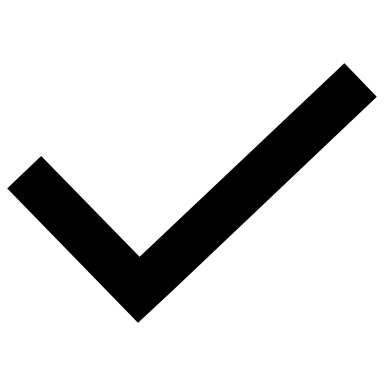 |  |  | Medium |
| P3 | Netley 1982 | 1982 | Canada | Cohort | 33 | 10.9 |  |  |  | 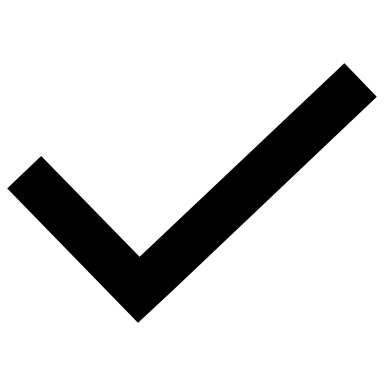 |  |  | Medium |
| P4 | Netley 1982b | 1982 | Canada | Cohort | 33 | 8.5 |  |  |  | 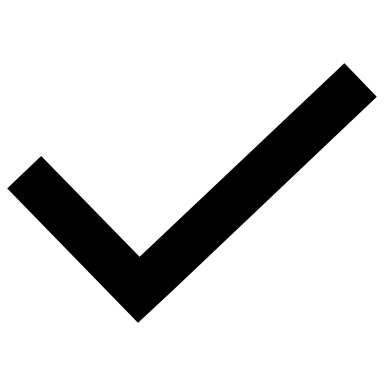 |  |  | High |
| P5 | Ratcliffe 1982 | 1982 | UK | Cohort | 32 | 5-16 | 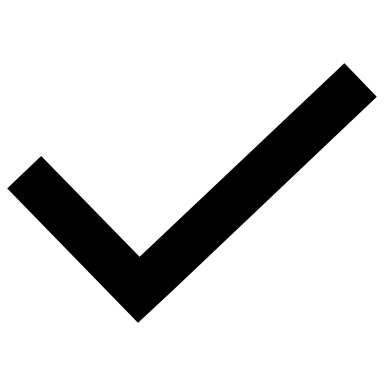 |  |  | 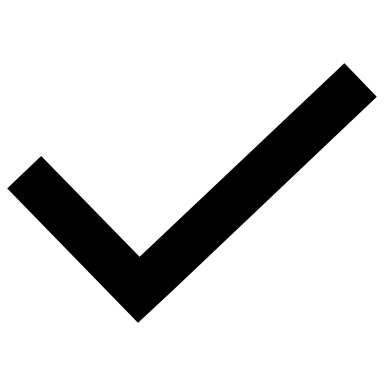 |  |  | High |
| P6 | Webber 1982 | 1982 | United States | Cohort | 11 | 9.2 |  | 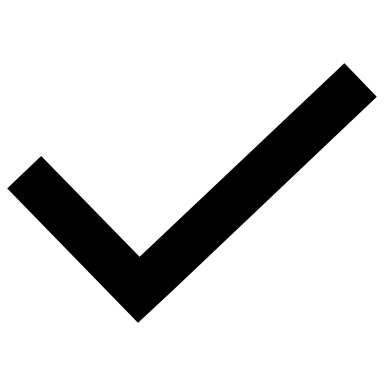 |  |  |  |  | High |
| P7 | Bender 1983 | 1983 | United states | Cross sectional | 14 | 12.0 |  |  |  | 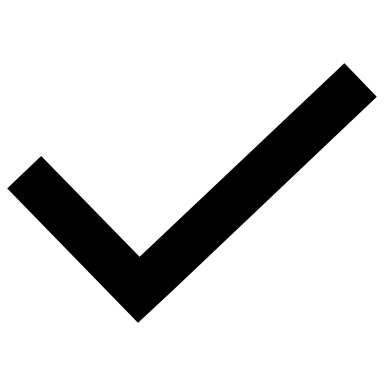 |  |  | Medium |
| P8 | Netley 1984 | 1984 | Canada | Cohort | 32 | 10.4 |  |  |  | 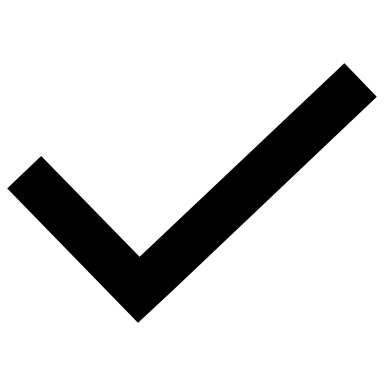 |  |  | Medium |
| P9 | Netley 1987 | 1987 | Canada | Cohort | 24 | 10.8 | 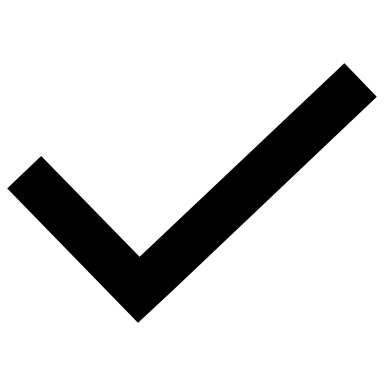 | 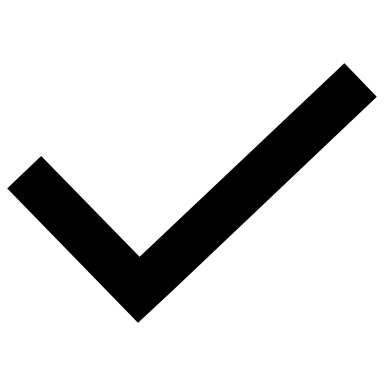 |  |  | 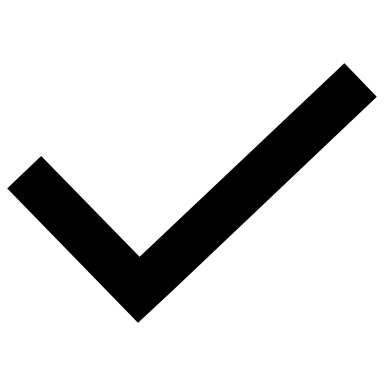 |  | Medium |
| P10 | Rovet 1996 | 1996 | Canada | Cohort | 29 | 1.6 |  |  |  | 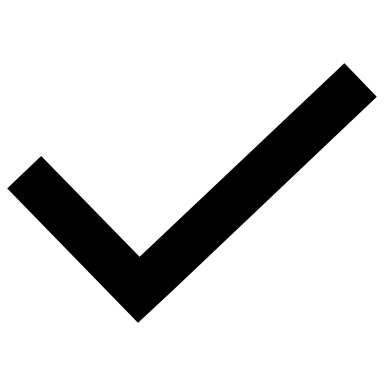 |  |  | High |
| P11 | Lahlou 2004 | 2004 | United States | Cohort | 24 | 0.1-18 | 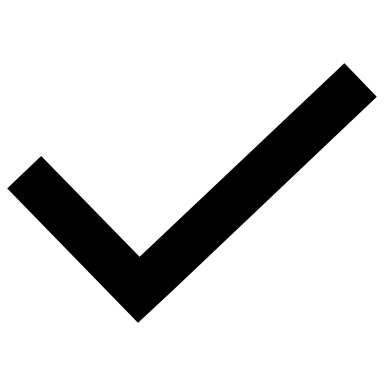 |  |  |  |  |  | High |
| P12 | Wikström 2004 | 2004 | Finland | Cohort | 14 | 10.1-14.0 | 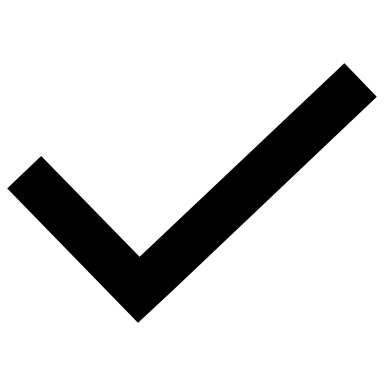 | 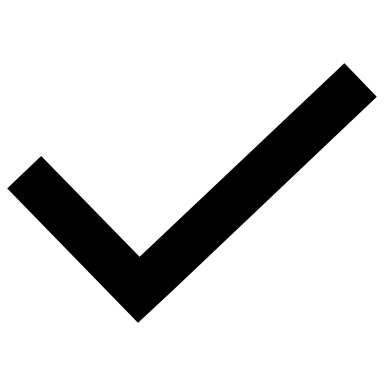 | 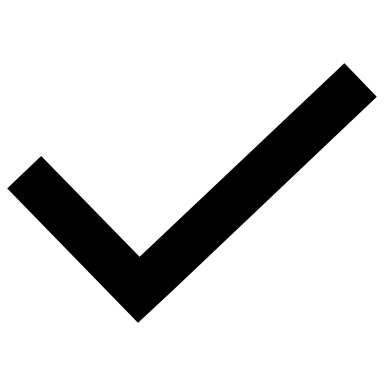 |  |  |  | High |
| P13 | Wikström 2006 | 2006 | Finland | Cohort | 14 | 10.0-13.9 | 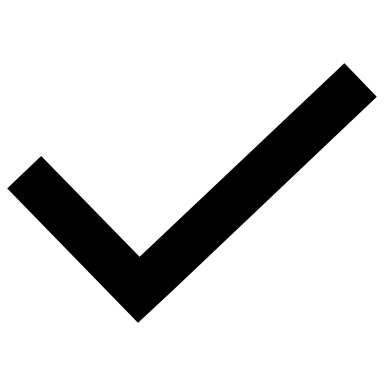 | 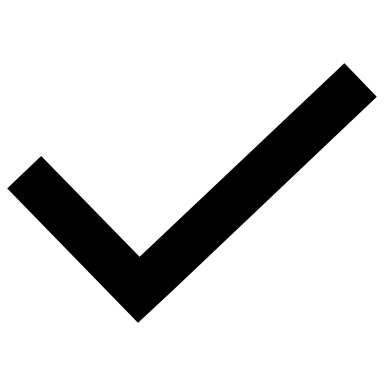 |  |  |  |  | High |
| P14 | Bastida 2007 | 2007 | Argentina | Cohort | 29 | 0.8-22.0 | 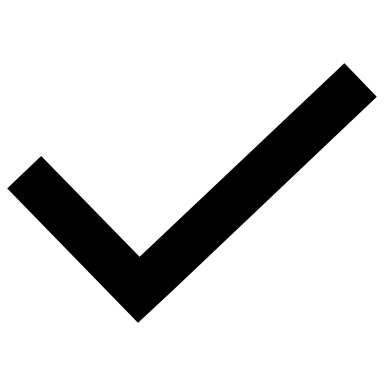 | 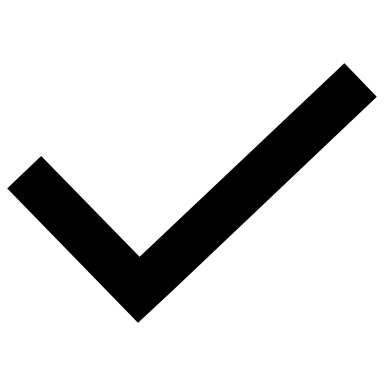 |  |  |  |  | High |
| P15 | Fine 2008 | 2008 | United States | Cohort | 30 | 12-18 | 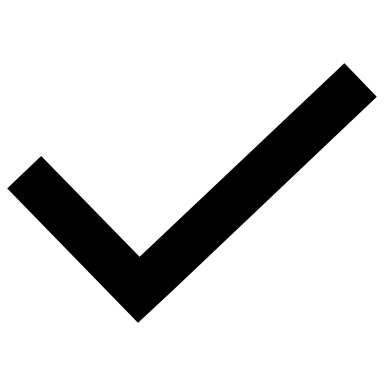 | 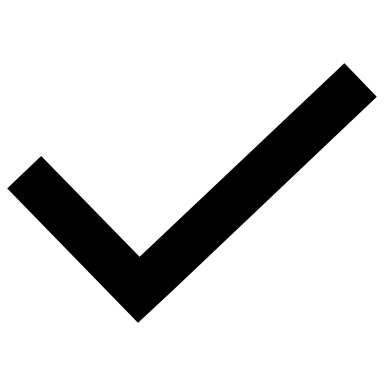 | 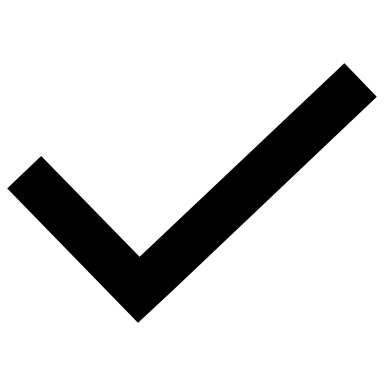 |  |  |  | Medium |
| P16 | Zeger 2008 | 2008 | United States | Cross sectional | 55 | 2-14.6 | 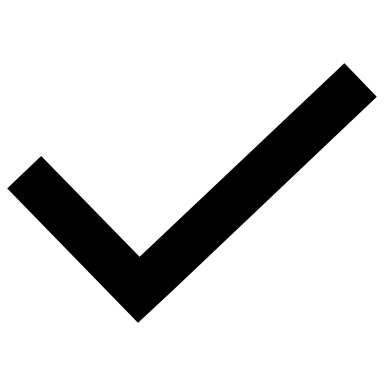 | 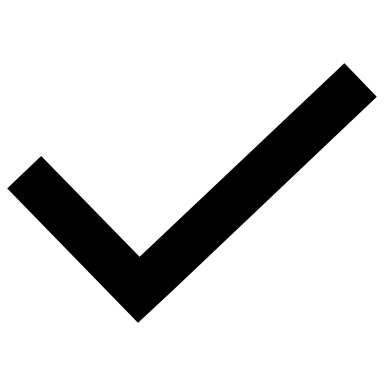 |  | 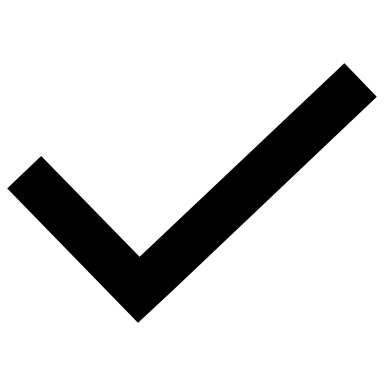 |  |  | High |
| P17 | Honig 2009 | 2009 | United States | Cohort | 5 | 17 | 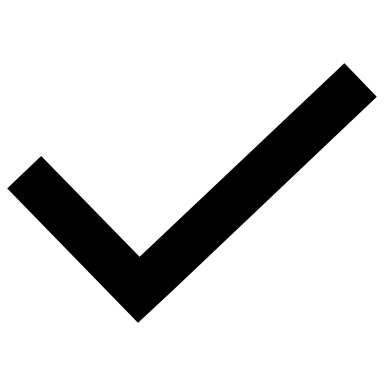 | 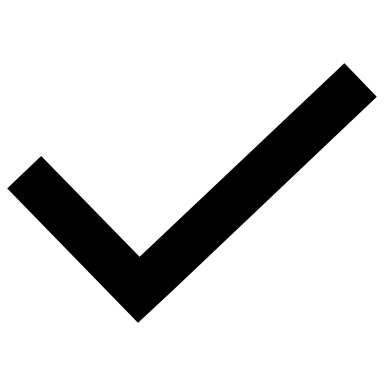 | 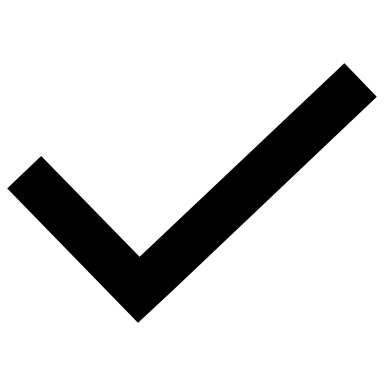 |  |  |  | Medium |
| P18 | Gies 2012 | 2012 | Belgium | Cohort | 7 | 13.6-15.6 | 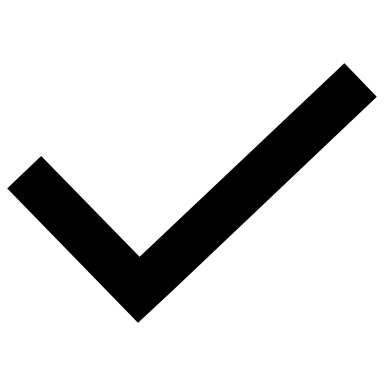 | 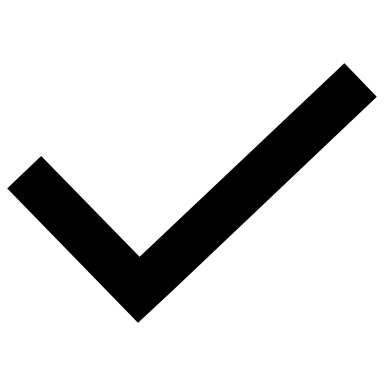 | 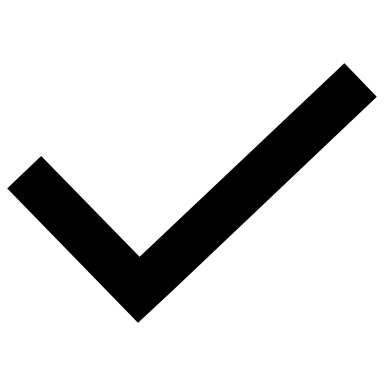 |  |  |  | Medium |
| P19 | Ross 2012 | 2012 | United States | Cohort | 82 | 9.2 |  | 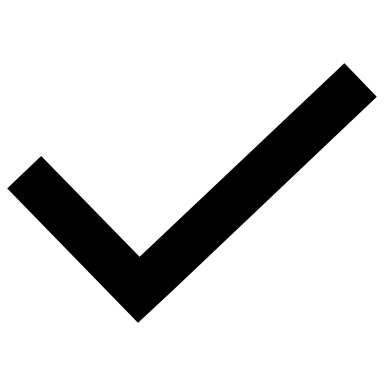 |  | 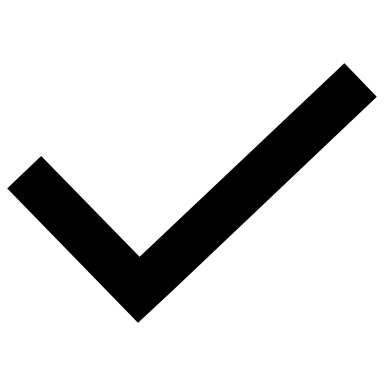 |  |  | High |
| P20 | Tincanci 2012 | 2012 | Spain | Cohort | 33 | 31.3 ± 12.9 | 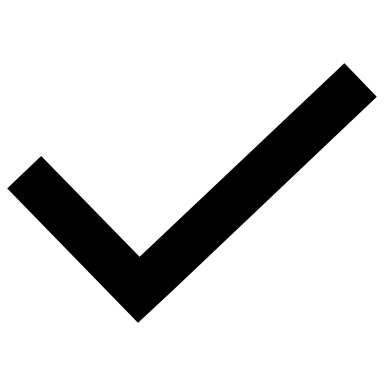 | 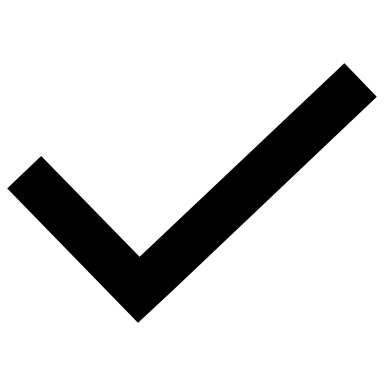 | 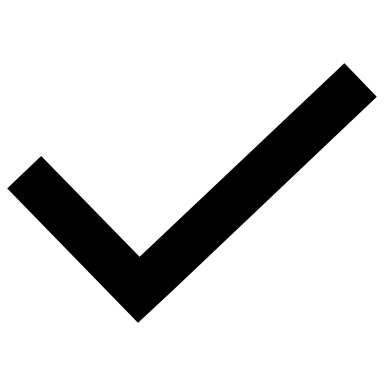 |  |  |  | Medium |
| P21 | Van Saen 2012 | 2012 | Belgium | Cohort | 7 | 13.3-15.9 | 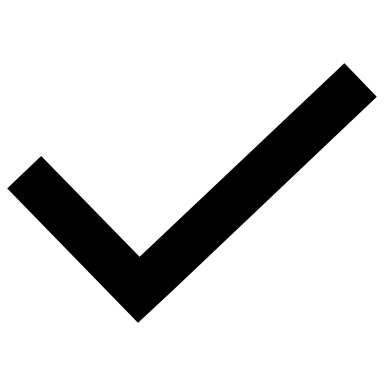 |  | 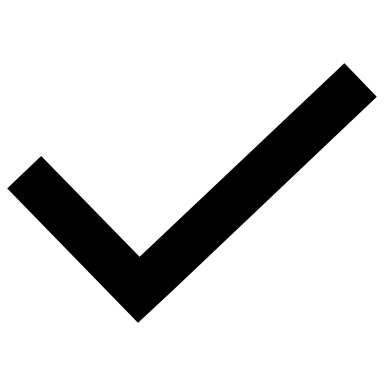 |  |  |  | High |
| P22 | Rives 2013 | 2013 | France | Cohort | 7 | 15-17 | 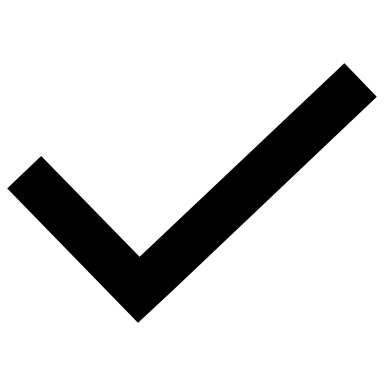 | 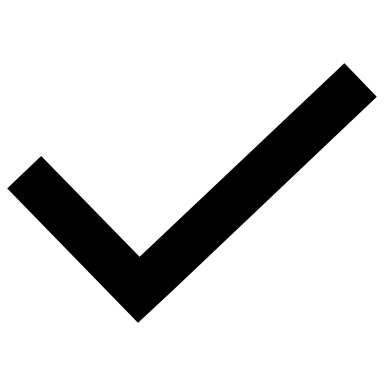 | 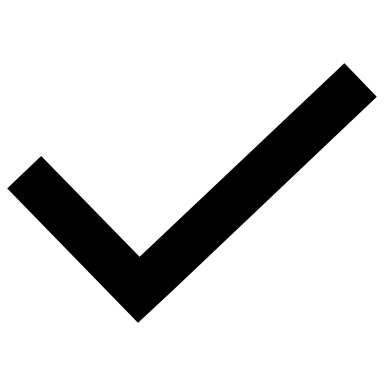 |  |  |  | High |
| P23 | Mehta 2014 | 2014 | United States | Cohort | 151 | 10-21 | 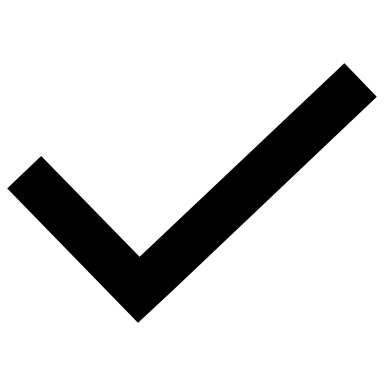 | 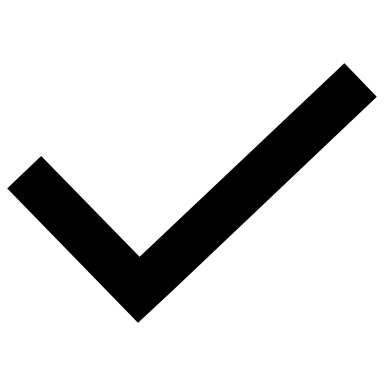 |  | 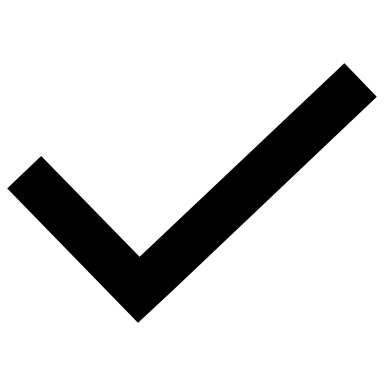 | 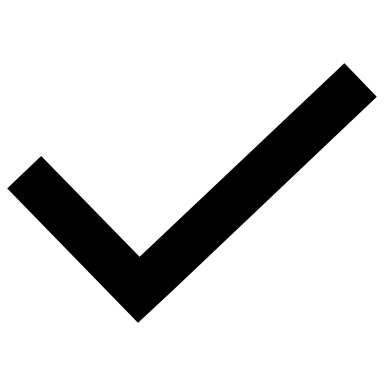 |  | High |
| P24 | Ross 2014 | 2014 | United States | RCT | 93 | 4.1 |  | 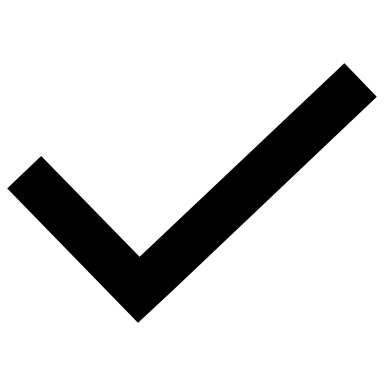 |  |  | 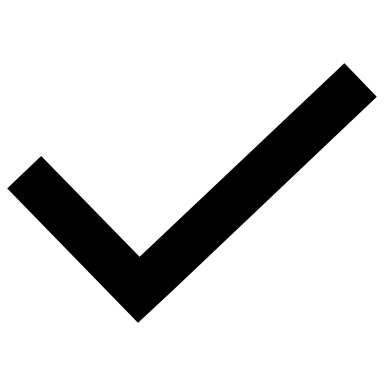 |  | High |
| P25 | Bardsley 2014 | 2014 | United States | RCT | 93 | 4-12 | 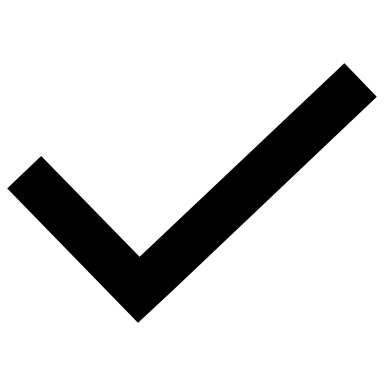 | 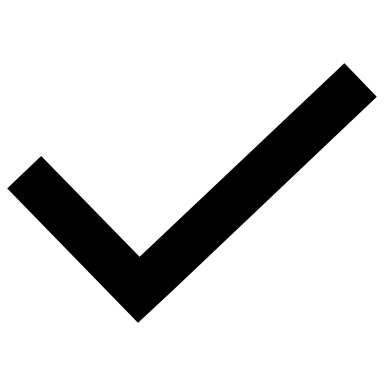 |  |  |  |  | High |
| P26 | Brandenburg-Goddard 2014 | 2014 | Netherlands | Cohort | 19 | 14.02 |  |  |  | 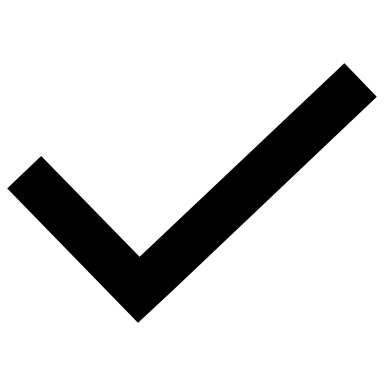 | 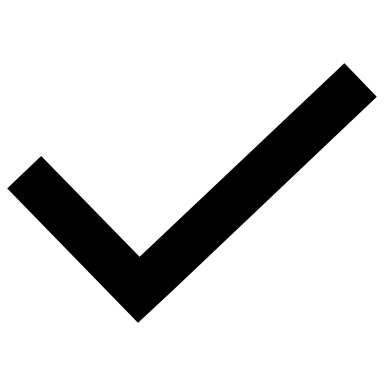 |  | High |
| P27 | Lahlou 2015 | 2015 | France | Cohort | 93 | 4-12 | 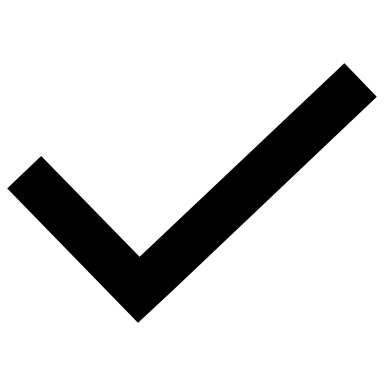 | 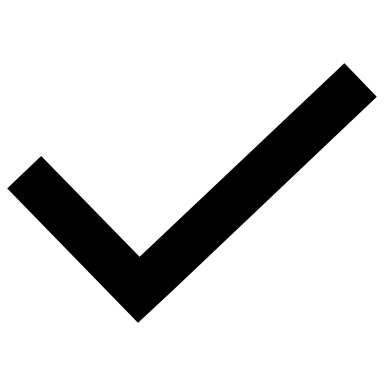 |  |  |  |  | Medium |
| P28 | Close 2015 | 2015 | United States | Cohort | 43 | 12.5 +/- 3.1 | 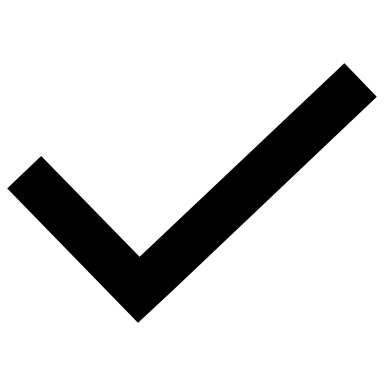 | 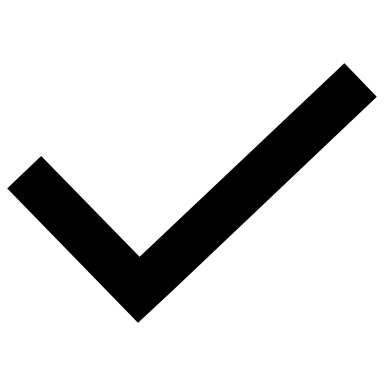 |  | 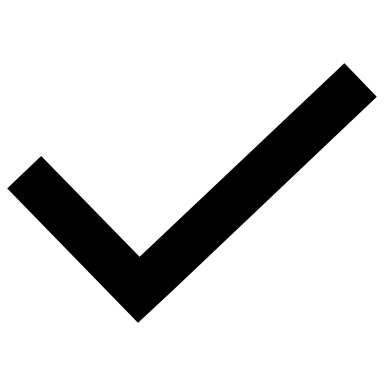 | 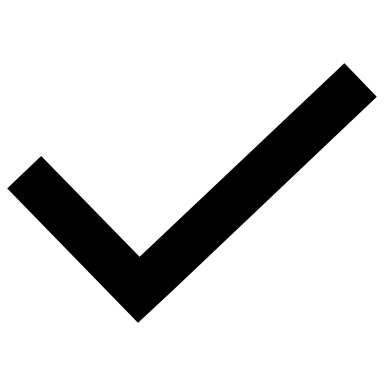 | 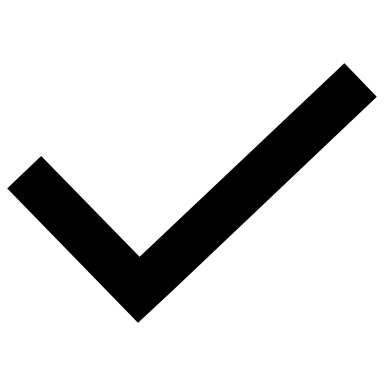 | High |
| P29 | Dotters-katz 2016 | 2016 | United States | Cohort | 52 | Perinatal |  |  |  |  | 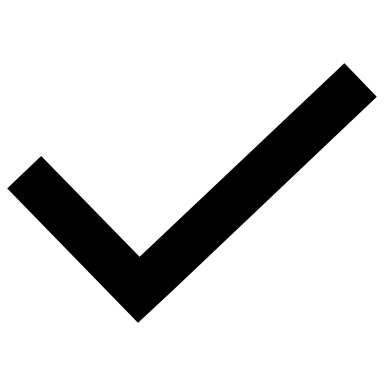 |  | High |
| P30 | Davis 2017 | 2017 | United States | RCT | 16 | 0.1-0.4 |  | 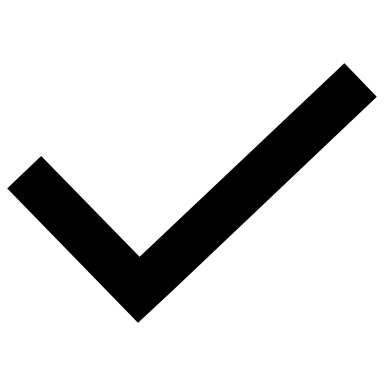 |  | 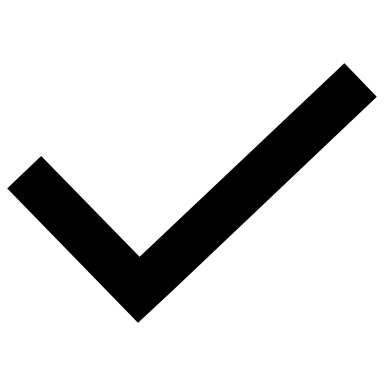 | 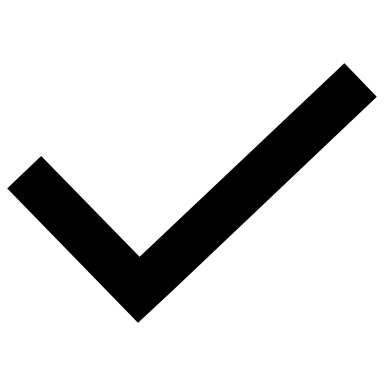 |  | Medium |
| P31 | Davis 2017b | 2017 | United States | RCT | 93 | 4-12 | 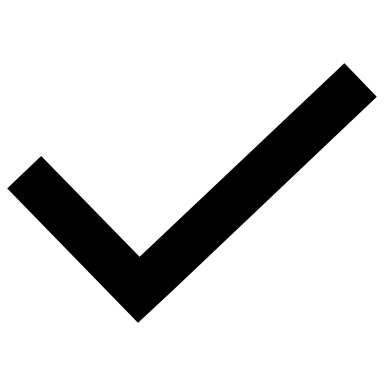 | 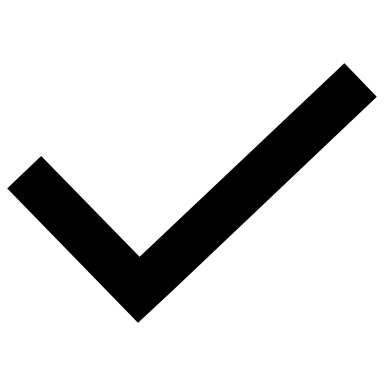 |  |  |  |  | High |
| P32 | Ross 2017 | 2017 | United States | RCT | 84 | 4-12 |  |  |  | 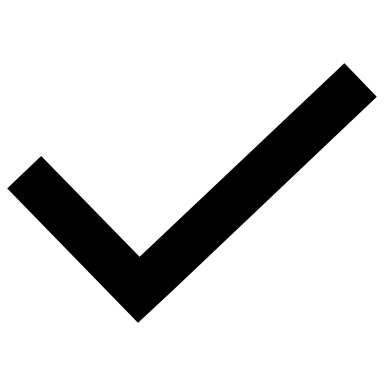 |  |  | High |
| P33 | Akcan 2018 | 2018 | Turkey, Cyprus | Cohort | 23 | 3.0 (0.04-16.3) | 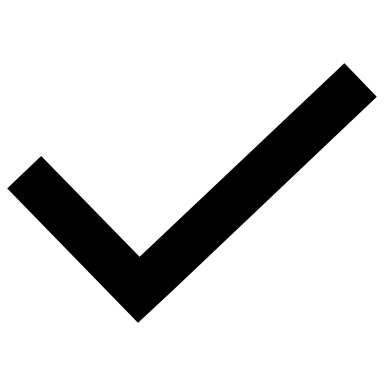 | 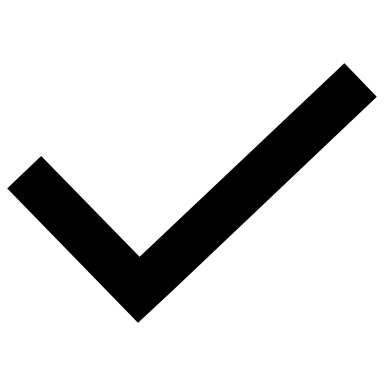 |  | 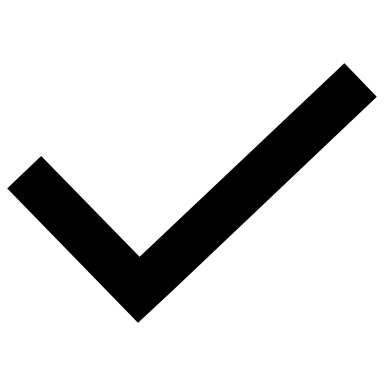 |  |  | Medium |
| P34 | Davis 2018 | 2018 | United States | RCT | 18 | 0.1-0.4 |  | 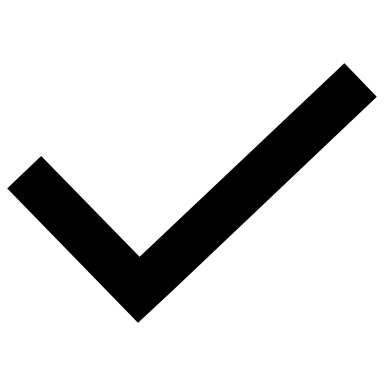 |  |  | 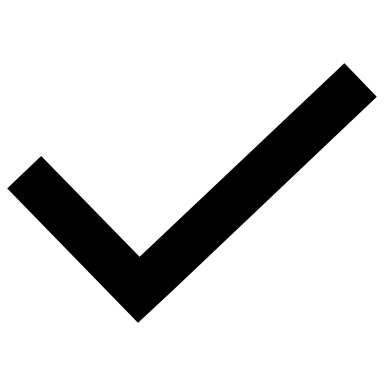 |  | Medium |
| P35 | Davis 2018b | 2018 | United States | RCT | 80 | 8.0 | 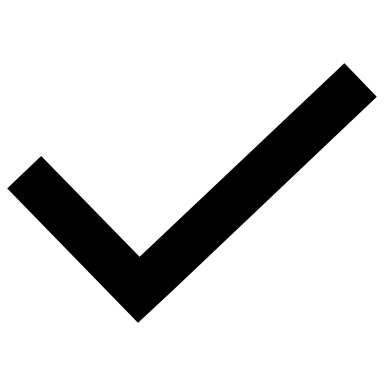 | 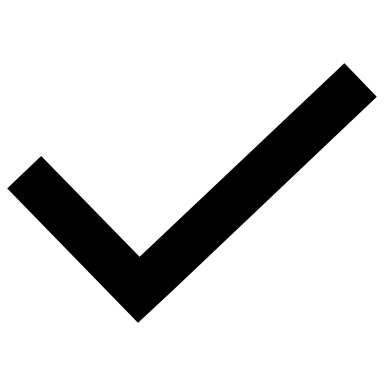 |  |  |  |  | Medium |
| P36 | StJohn 2019 | 2019 | Australia | Cohort | 41 | 1-17 |  | 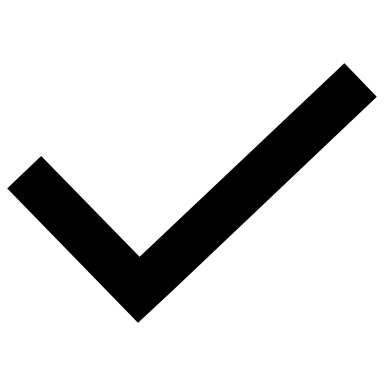 |  | 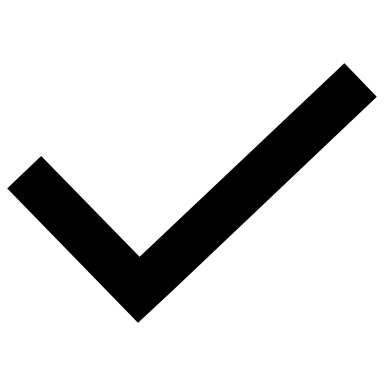 | 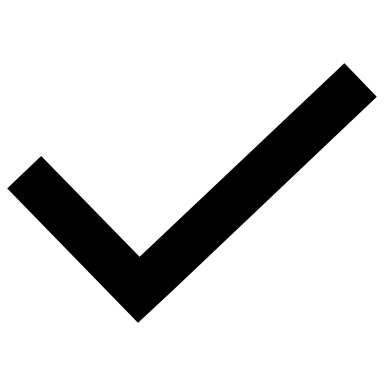 |  | High |
| P37 | Davis 2019 | 2019 | United States | RCT | 20 | 0.1-0.4 |  | 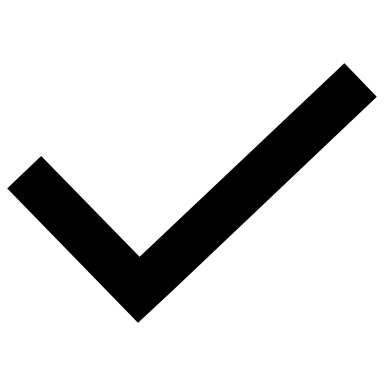 |  |  |  |  | High |
| P38 | Foland-Ross 2019 | 2019 | United States | RCT | 23 | 10.9 |  |  |  | 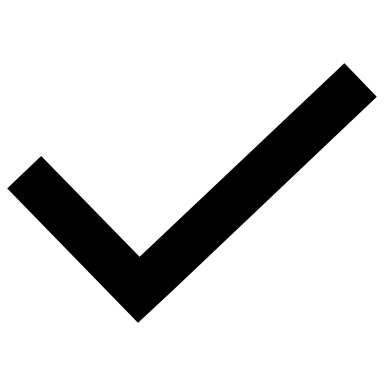 | 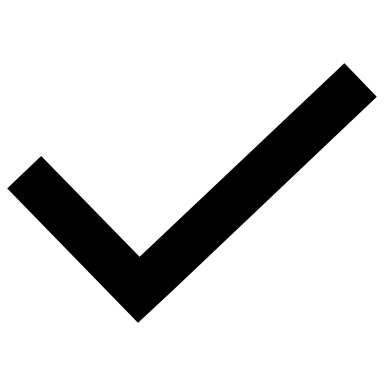 |  | High |
| P39 | Brooks 2021 | 2021 | United States | RCT | 175 | 0.1-4 |  |  |  | 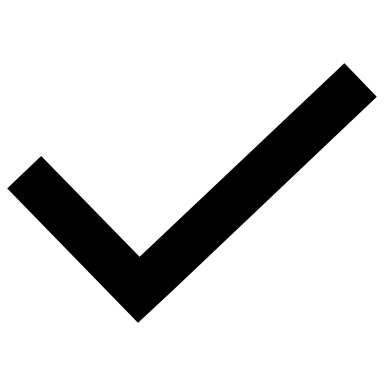 |  |  | Medium |
| P40 | Butler 2021 | 2021 | United Kingdom | Cohort | 59 | 12.3 +/-1.8 |  | 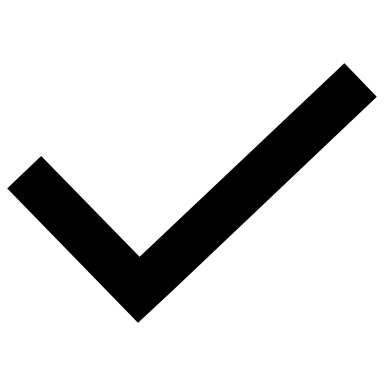 |  |  |  |  | High |
| P41 | Samango-Sprouse 2021 | 2021 | United States | RCT | 175 | 0.1-4 |  |  |  | 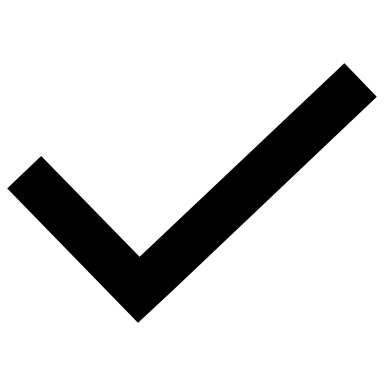 |  |  | Medium |
| P42 | Foland-Ross 2021 | 2021 | United States | Cohort | 19 | 11.5 |  | 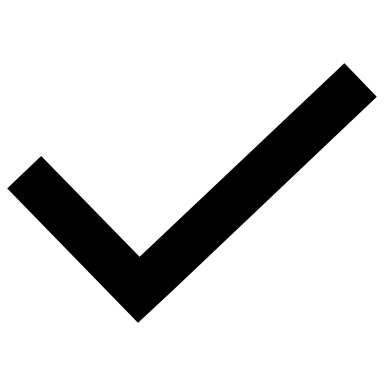 |  | 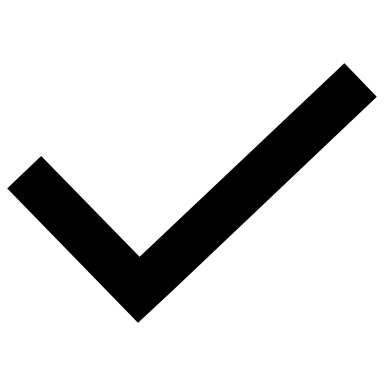 | 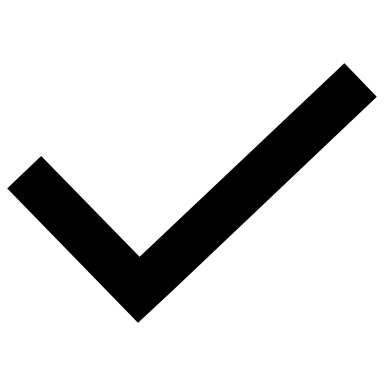 |  | High |
| P43 | Hamzik 2021 | 2021 | United States | Cohort | 56 | 7-11 |  |  |  | 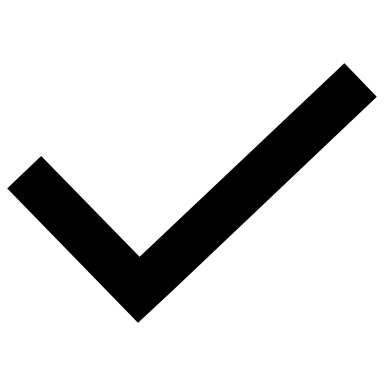 |  |  | Medium |
| P44 | Vogiatzi 2021 | 2021 | United States | RCT | 89 | 6.9 | 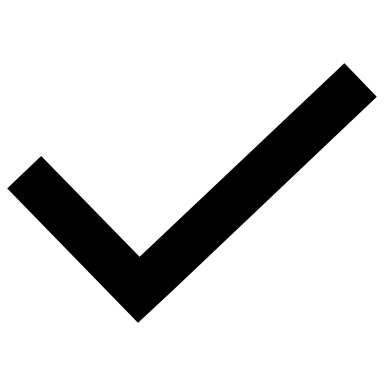 | 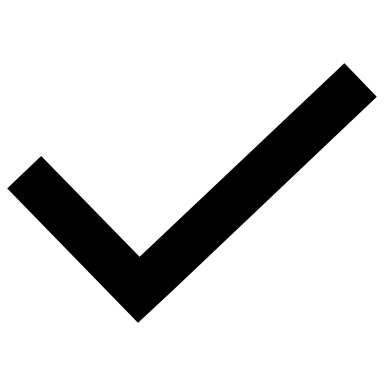 |  |  | 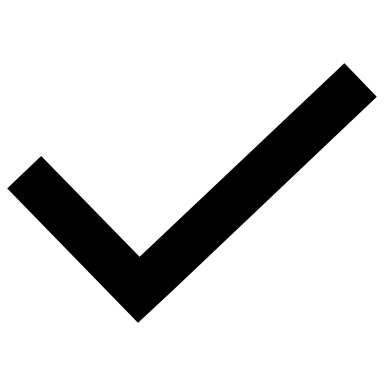 |  | High |
| P45 | Funke 2021 | 2021 | Germany, Sweden, Finland | Multicentre | 10 | 14.2 |  |  | 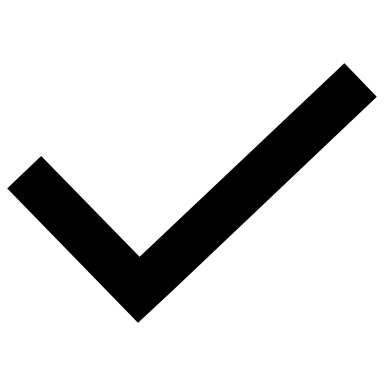 |  |  |  | High |
| P46 | Tanner 2021 | 2021 | Finland | Cohort | 72 | - | 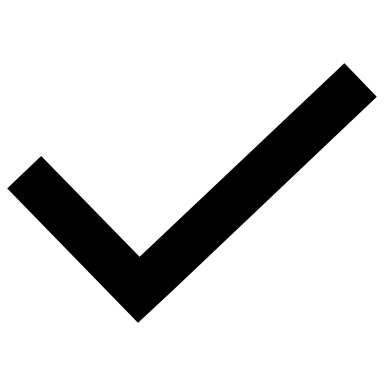 | 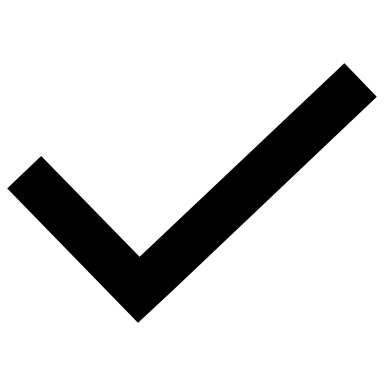 |  |  |  |  |  |
| P47 | Samango-Sprouse 2022 | 2022 | United States | RCT | 223 | 0.1-6 |  |  |  | 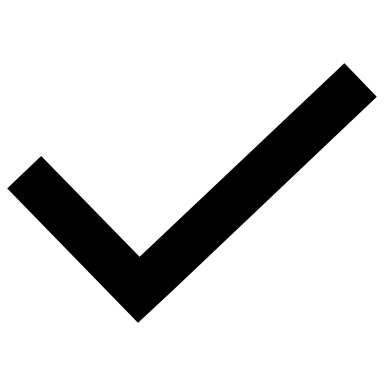 |  |  | Medium |
| P48 | Brooks 2022 | 2022 | United States | Cross sectional | 111 | 9.4 |  |  |  | 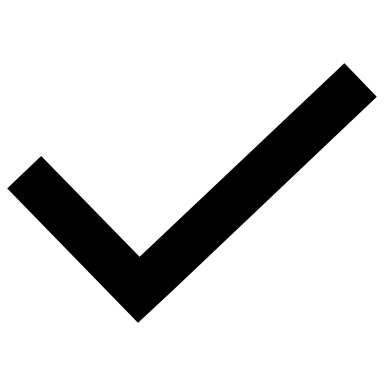 |  |  | Medium |
| P49 | Davis 2022 | 2022 | United States | RCT | 71 | 0.2 |  |  |  | 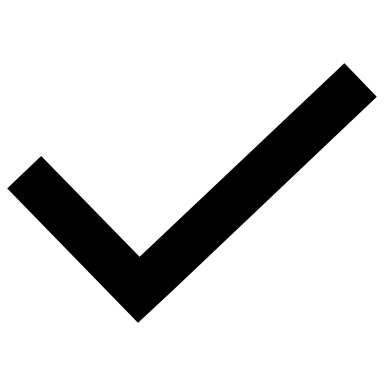 |  |  | High |
| P50 | Davis 2022b | 2022 | United States | Cross sectional | 1080 | 13.5 |  |  |  |  |  |  | High |
| P51 | Samango-Sprouse 2023 | 2023 | United States | RCT | 169 | 0-3 |  |  |  |  |  |  | Medium |
| P52 | Davis 2023 | 2023 | United States | RCT | 71 | 0.3 |  |  |  |  |  |  | High |
| P53 | Taylor 2023 | 2023 | United States | RCT | 169 | 5-18 |  |  |  |  |  |  | High |
| P54 | Jordan 2023 | 2023 | United States | Cohort | 47 KS, 55 TD | 11.95 |  |  |  |  |  |  | High |
| P55 | Pozza 2023 | 2023 | Italy | Cohort | 155 | 0.58-55 |  |  |  |  |  |  | High |
| P56 | Spiekermann 2023 | 2023 | Germany | Cohort | 19 | 15.9 |  |  |  |  |  |  | High |

Supplementary Table 2. Table summarising the 47 included studies in boys ≤16 years with KS. Data shown includes the author, year, country of origin, type of study, participant number, mean age ± standard deviation (SD) (years) or median* or range if mean/median were unavailable. Outcome data included biochemistry (Bio), phenotype/anthropometry (PA), fertility (Fe), cognitive, psychological, developmental and behavioural (CP), co-morbidities (CM) and quality of life (QoL). The quality of each study was assessed as high, medium, or low based on the relevant Critical Appraisal Skills Programme checklists .

| \|  \| **Author** \| **Year** \| **Country of origin** \| **Study design** \| **Participant no** \| **Mean age ± SD/range (years)** \| **Bio** \| **PA** \| **Fe** \| **CP** \| **CM** \| **QoL** \| **Quality** \| \| --- \| --- \| --- \| --- \| --- \| --- \| --- \| --- \| --- \| --- \| --- \| --- \| --- \| --- \| \| A1 \| Mosier et al \| 1960 \| USA \| Cohort \| 10 \| N/A \|  \|  \|  \| ✔️ \|  \|  \| Medium \| \| A2 \| Rohde \| 1963 \| USA \| Cohort \| 8 \| 49 \| ✔️ \| ✔️ \|  \| ✔️ \| ✔️ \|  \| High \| \| A3 \| Money \| 1964 \| USA \| Cohort \| 23 \| 14-76 \|  \|  \|  \| ✔️ \|  \|  \| Medium \| \| A4 \| Vallotton and Forbes \| 1967 \| USA \| Cohort \| 25 \| 14-78 \|  \|  \|  \|  \| ✔️ \|  \| Medium \| \| A5 \| Myhre et al \| 1970 \| USA \| Cohort \| 5 \| 9-24 \| ✔️ \| ✔️ \|  \| ✔️ \|  \|  \| Medium \| \| A6 \| Stewart-Bentley and Horton \| 1973 \| USA \| Cohort \| 8 \| 17-45 \| ✔️ \|  \|  \|  \|  \|  \| Medium \| \| A7 \| Money et al \| 1974 \| USA \| Cohort \| 12 \| 25 \|  \|  \|  \| ✔️ \|  \|  \| Medium \| \| A8 \| Kosowicz and Rzymski \| 1975 \| Poland \| Cohort \| 21 \| 12-31 \|  \| ✔️ \|  \|  \|  \|  \| Medium \| \| A9 \| Wang et al \| 1975 \| Australia \| Cohort \| 19 \| 28.9 \| ✔️ \| ✔️ \|  \| ✔️ \|  \|  \| Medium \| \| A10 \| Philip et al \| 1976 \| Denmark \| Cohort \| 16 \| N/A \|  \| ✔️ \|  \|  \|  \|  \| Medium \| \| A11 \| Boisen \| 1979 \| Denmark \| Cohort \| 14 \| 26-31 \|  \| ✔️ \|  \|  \|  \|  \| High \| \| A12 \| Umapathy et al \| 1980 \| India \| Cohort \| 17 \| 20-35 \|  \|  \| ✔️ \|  \|  \|  \| Medium \| \| A13 \| Cheikh et al \| 1981 \| USA \| Cohort \| 4 \| 35 \| ✔️ \|  \|  \|  \| ✔️ \|  \| Medium \| \| A14 \| Nistal et al \| 1982 \| Spain \| Cohort \| 14 \| 16-35 \|  \|  \| ✔️ \|  \|  \|  \| Medium \| \| A15 \| Wu et al \| 1982 \| United Kingdom \| Cohort \| 4 \| 35.25 \| ✔️ \|  \|  \| ✔️ \|  \|  \| Medium \| \| A16 \| Paniagua et al \| 1984 \| Spain \| Cohort \| 19 \| 16-35 \|  \|  \| ✔️ \|  \|  \|  \| Medium \| \| A17 \| Goffi et al \| 1985 \| Italy \| Cohort \| 8 \| 18-31 \| ✔️ \|  \|  \|  \|  \|  \| Medium \| \| A18 \| Bizzarro et al \| 1987 \| Italy \| Cohort \| 5 \| 18-30 \|  \|  \|  \|  \| ✔️ \|  \| Medium \| \| A19 \| Horowitz et al \| 1992 \| Australia \| Cohort \| 22 \| 37 \| ✔️ \| ✔️ \|  \|  \| ✔️ \|  \| High \| \| A20 \| Laine et al \| 1992 \| Finland \| Cohort \| 29 \| 26 ± 10.7 \|  \| ✔️ \|  \|  \|  \|  \| Medium \| \| A21 \| Babić et al \| 1993 \| Yugoslavia \| Cohort \| 28 \| 22-31 \|  \| ✔️ \|  \|  \|  \|  \| Medium \| \| A22 \| Choi et al \| 1995 \| Korea \| Cohort \| 20 \| 26.5 ± 1.12 \| ✔️ \| ✔️ \|  \|  \| ✔️ \|  \| Medium \| \| A23 \| Luisetto et al \| 1995 \| Italy \| Cohort \| 32 \| 25.4 ± 5.1 \| ✔️ \|  \|  \|  \| ✔️ \|  \| Medium \| \| A24 \| Luboshitzky et al \| 1997 \| Israel \| Cohort \| 6 \| 17.5 \| ✔️ \|  \|  \|  \|  \|  \| Medium \| \| A25 \| Yoshida et al \| 1997 \| Japan \| Cohort \| 40 \| 32.2 ± 4.0 \| ✔️ \|  \| ✔️ \| ✔️ \|  \|  \| Medium \| \| A26 \| Leifke et al \| 1998 \| Germany \| Cohort \| 12 \| 35.3 ± 5.2 \| ✔️ \|  \|  \|  \| ✔️ \|  \| High \| \| A27 \| Tatum IV et al \| 1998 \| USA \| Multicentre \| 12 \| 12-48 \|  \|  \|  \| ✔️ \| ✔️ \|  \| Medium \| \| A28 \| Tateno et al \| 1999 \| Japan \| Cohort \| 21 \| 23-35 \| ✔️ \|  \| ✔️ \|  \|  \|  \| High \| \| A29 \| Bremner et al \| 2000 \| China \| Cohort \| 8 \| 16-26 \| ✔️ \| ✔️ \|  \|  \|  \|  \| Medium \| \| A30 \| Levron et al \| 2000 \| Israel \| Cohort \| 20 \| N/A \| ✔️ \|  \| ✔️ \|  \|  \|  \| Medium \| \| A31 \| Patwardhan et al \| 2000 \| USA \| Cohort \| 10 \| 27.32 ± 2.99 \|  \| ✔️ \|  \| ✔️ \|  \|  \| Medium \| \| A32 \| De Rosa et al \| 2001 \| Italy \| Cohort \| 8 \| 29.6 ± 2.6 \| ✔️ \|  \|  \|  \| ✔️ \|  \| Medium \| \| A33 \| Levron et al \| 2001 \| Israel \| Cohort \| 5 \| N/A \| ✔️ \|  \| ✔️ \|  \|  \|  \| Medium \| \| A34 \| van den Bergh et al \| 2001 \| Netherlands \| Cohort \| 52 \| 39.1 ± 12.4 \| ✔️ \|  \|  \|  \| ✔️ \|  \| High \| \| A35 \| Westlander et al \| 2001 \| Sweden \| Cohort \| 19 \| 33.7 ± 3.8 \| ✔️ \| ✔️ \| ✔️ \|  \|  \|  \| High \| \| A36 \| Keung et al \| 2002 \| USA \| Cohort \| 31 \| 0-81 \|  \|  \|  \|  \| ✔️ \|  \| Medium \| \| A37 \| Madgar et al \| 2002 \| Israel \| Cohort \| 20 \| 32.2 ± 5.0 \| ✔️ \| ✔️ \| ✔️ \|  \|  \|  \| Medium \| \| A38 \| Oktenli et al \| 2002 \| Turkey \| Cohort \| 37 \| 21.54 ± 1.10 \| ✔️ \|  \|  \|  \| ✔️ \|  \| High \| \| A39 \| Raman and Schlegel \| 2002 \| USA \| Cohort \| 17 \| N/A \| ✔️ \|  \| ✔️ \|  \|  \|  \| High \| \| A40 \| Christiansen et al \| 2003 \| Denmark \| Cohort \| 15 \| 20.6* \| ✔️ \|  \|  \|  \|  \|  \| Medium \| \| A41 \| Kamischke et al \| 2003 \| Germany \| Cohort \| 85 \| 29.1 ± 1.1 \| ✔️ \| ✔️ \| ✔️ \|  \|  \|  \| High \| \| A42 \| Stepan et al \| 2003 \| Czech Republic \| Cohort \| 14 \| 55.2 ± 5.4 \| ✔️ \|  \|  \|  \| ✔️ \|  \| High \| \| A43 \| Bojesen et al \| 2004 \| Denmark \| Cohort \| 781 \| N/A \|  \|  \|  \|  \| ✔️ \|  \| Medium \| \| A44 \| Chiang et al \| 2004 \| Taiwan \| Cohort \| 14 \| 33 ± 4 \| ✔️ \|  \| ✔️ \|  \|  \|  \| Medium \| \| A45 \| Okada et al \| 2004 \| Japan \| Cohort \| 24 \| N/A \| ✔️ \| ✔️ \|  \|  \|  \|  \| Medium \| \| A46 \| Seo et al \| 2004 \| Korea \| Cohort \| 42 \| 32 ± 3.0 \| ✔️ \| ✔️ \| ✔️ \|  \|  \|  \| Medium \| \| A47 \| Wielgos et al \| 2004 \| Poland \| Cohort \| 14 \| 25.92 ± 4.44 \| ✔️ \|  \|  \|  \|  \|  \| Medium \| \| A48 \| Yesilova et al \| 2004 \| Turkey \| Cohort \| 32 \| 21.66 ± 1.15 \| ✔️ \|  \|  \|  \|  \|  \| Medium \| \| A49 \| Okada et al \| 2005 \| Japan \| Multicentre \| 51 \| 25-43 \| ✔️ \|  \| ✔️ \|  \|  \|  \| High \| \| A50 \| Schiff et al \| 2005 \| USA \| Cohort \| 42 \| 32.8 \| ✔️ \|  \| ✔️ \|  \|  \|  \| High \| \| A51 \| Swerdlow et al \| 2005 \| United Kingdom \| Cohort \| 3518 \| N/A \|  \|  \|  \|  \| ✔️ \|  \| High \| \| A52 \| Swerdlow et al \| 2005 \| United Kingdom \| Cohort \| 3518 \| N/A \|  \|  \|  \|  \| ✔️ \|  \| High \| \| A53 \| Yesilova et al \| 2005 \| Turkey \| Cohort \| 13 \| 22.08 ± 1.38 \| ✔️ \|  \|  \|  \|  \|  \| High \| \| A54 \| Bakircioglu et al \| 2006 \| Turkey \| Cohort \| 74 \| 25-50 \| ✔️ \| ✔️ \| ✔️ \|  \|  \|  \| High \| \| A55 \| Bojesen et al \| 2006 \| Denmark \| Cohort \| 832 \| N/A \|  \|  \|  \|  \| ✔️ \|  \| High \| \| A56 \| Bojesen et al \| 2006 \| Denmark \| Cohort \| 71 \| 19-66.2 \| ✔️ \| ✔️ \|  \|  \| ✔️ \|  \| High \| \| A57 \| van Rijn et al \| 2006 \| Netherlands \| Cohort \| 32 \| 38.8 ± 8.3 \|  \|  \|  \| ✔️ \|  \|  \| Medium \| \| A58 \| Koga \| 2007 \| Japan \| Cohort \| 26 \| 36 ± 4.7 \| ✔️ \| ✔️ \| ✔️ \|  \|  \|  \| High \| \| A59 \| Lähdesmäki and Alvesalo \| 2007 \| Finland \| Cohort \| 39 \| 30.7 \|  \| ✔️ \|  \|  \|  \|  \| Medium \| \| A60 \| Seo et al \| 2007 \| Korea \| Cohort \| 40 \| 32.05 ± 3.10 \| ✔️ \|  \|  \|  \| ✔️ \|  \| Medium \| \| A61 \| Ishikawa et al \| 2008 \| Japan \| Cohort \| 60 \| 33.6 ± 5.3 \| ✔️ \| ✔️ \| ✔️ \|  \| ✔️ \|  \| High \| \| A62 \| Takada et al \| 2008 \| Japan \| Cohort \| 9 \| 33.9 ± 0.5 \| ✔️ \|  \| ✔️ \|  \|  \|  \| High \| \| A63 \| van Rijn et al \| 2008 \| Netherlands \| Cohort \| 31 \| 41.3 ± 10.0 \|  \|  \|  \| ✔️ \|  \|  \| Medium \| \| A64 \| van Rijn et al \| 2008 \| Netherlands \| Cohort \| 15 \| 36.9 ± 11.8 \|  \|  \|  \| ✔️ \|  \|  \| High \| \| A65 \| de Ronde et al \| 2009 \| Netherlands \| Cohort \| 40 \| 46 ± 11.8 \|  \|  \|  \|  \|  \| ✔️ \| Medium \| \| A66 \| Ferhi et al \| 2009 \| France \| Cohort \| 27 \| 25-42 \| ✔️ \| ✔️ \| ✔️ \|  \|  \|  \| High \| \| A67 \| Ramasamy et al \| 2009 \| USA \| Cohort \| 68 \| 33 ± 6 \| ✔️ \| ✔️ \| ✔️ \|  \|  \|  \| Medium \| \| A68 \| van Rijn et al \| 2009 \| Netherlands \| Cohort \| 24 \| 41.05 \|  \|  \|  \| ✔️ \|  \|  \| High \| \| A69 \| Yarali et al \| *2009* \| Turkey \| Cohort \| 33 \| 32.0 ± 6.4 \|  \|  \| ✔️ \|  \|  \|  \| Medium \| \| A70 \| Bruining et al \| 2010 \| Netherlands \| Cohort \| 43 \| 31.1 ± 14.4 \|  \|  \|  \| ✔️ \|  \|  \| Medium \| \| A71 \| Corona et al \| 2010 \| Italy \| Cohort \| 23 \| 40.6 ± 12.3 \| ✔️ \| ✔️ \| ✔️ \| ✔️ \|  \|  \| High \| \| A72 \| Aksglæde et al \| 2011 \| Denmark \| Cohort \| 166 \| 0.3–80.3 \| ✔️ \| ✔️ \| ✔️ \| ✔️ \| ✔️ \|  \| High \| \| A73 \| Bakircioglu et al \| 2011 \| Turkey \| Cohort \| 106 \| 34.3±5.9 \|  \|  \| ✔️ \|  \|  \|  \| High \| \| A74 \| Bojesen et al \| 2011 \| Denmark \| Cohort \| 70 \| 35.5* \| ✔️ \| ✔️ \|  \|  \| ✔️ \|  \| High \| \| A75 \| Herlihy et al \| 2011 \| Australia \| Cohort \| 87 \| 43 \|  \| ✔️ \| ✔️ \| ✔️ \|  \|  \| High \| \| A76 \| Kompus et al \| 2011 \| Norway \| Cohort \| 27 \| 37.7 ± 9.1 \|  \|  \|  \| ✔️ \|  \|  \| Medium \| \| A77 \| Melhem et al \| 2011 \| USA \| Cohort \| 3 \| N/A \|  \|  \|  \| ✔️ \|  \|  \| Medium \| \| A78 \| Trabado et al \| 2011 \| France \| Cohort \| 45 \| 34.5 ± 11.8 \| ✔️ \|  \|  \|  \|  \|  \| Medium \| \| A79 \| Turriff et al \| 2011 \| USA \| Cohort \| 310 \| 14-75 \|  \|  \|  \| ✔️ \|  \|  \| High \| \| A80 \| Van Rijn and Swaab \| 2011 \| Netherlands \| Cohort \| 48 \| 39.0 ± 11.8 \|  \|  \|  \| ✔️ \|  \|  \| High \| \| A81 \| Bak et al \| 2012 \| Korea \| Cohort \| 179 \| 32.85 ± 3.52 \| ✔️ \| ✔️ \| ✔️ \|  \|  \|  \| High \| \| A82 \| Dillon et al \| 2012 \| USA \| Cohort \| 7 \| N/A \|  \|  \|  \|  \| ✔️ \|  \| High \| \| A83 \| Foresta et al \| 2012 \| Italy \| Cohort \| 92 \| 31.5 ± 8.7 \| ✔️ \| ✔️ \|  \|  \| ✔️ \|  \| High \| \| A84 \| Jiang-Feng et al \| 2012 \| China \| Cohort \| 39 \| 1-30 \| ✔️ \| ✔️ \|  \|  \| ✔️ \|  \| High \| \| A85 \| Kota et al \| 2012 \| India \| Cohort \| 5 \| 20.8 ± 9.8 \|  \|  \|  \|  \| ✔️ \|  \| Medium \| \| A86 \| Pacenza et al \| 2012 \| Argentina \| Cohort \| 98 \| 0-60 \| ✔️ \| ✔️ \| ✔️ \| ✔️ \|  \|  \| High \| \| A86 \| van Rijn et al \| 2012 \| Netherlands \| Cohort \| 41 \| 28.9 ± 16.6 \|  \|  \|  \| ✔️ \|  \|  \| High \| \| A87 \| Van Saen et al \| 2012 \| Belgium \| Cohort \| 22 \| 24–43 \|  \|  \| ✔️ \|  \|  \|  \| Medium \| \| A88 \| Ando et al \| 2013 \| Japan \| Cohort \| 35 \| 34.4 ± 4.6 \| ✔️ \| ✔️ \| ✔️ \|  \|  \|  \| Medium \| \| A89 \| Inci et al \| 2013 \| Turkey \| Cohort \| 33 \| 36.24 ± 5.51 \| ✔️ \| ✔️ \|  \|  \|  \|  \| Medium \| \| A90 \| Jo et al \| 2013 \| Korea \| Cohort \| 18 \| 30-44 \| ✔️ \| ✔️ \|  \|  \| ✔️ \|  \| Medium \| \| A91 \| Mehta et al \| 2013 \| USA \| Cohort \| 10 \| 15.5 \| ✔️ \| ✔️ \| ✔️ \|  \|  \|  \| High \| \| A92 \| Pasquali et al \| 2013 \| Italy \| Cohort \| 69 \| 30.5 ± 3 \| ✔️ \| ✔️ \|  \|  \| ✔️ \|  \| High \| \| A93 \| Selice et al \| 2013 \| Italy \| Cohort \| 121 \| 31.5 ± 8.7 \| ✔️ \| ✔️ \|  \|  \| ✔️ \|  \| High \| \| A94 \| Brinton et al \| 2014 \| USA \| Meta-analysis \| 8 \| 67* \|  \|  \|  \|  \| ✔️ \|  \| Medium \| \| A95 \| Bryson et al \| 2014 \| USA \| Cohort \| 127 \| N/A \|  \| ✔️ \| ✔️ \|  \|  \|  \| High \| \| A96 \| Cederlöf et al \| 2014 \| Sweden \| Cohort \| 860 \| N/A \|  \|  \|  \| ✔️ \|  \|  \| High \| \| A97 \| Overvad et al \| 2014 \| Denmark \| Cohort \| 70 \| 38.6 ± 12.4 \| ✔️ \| ✔️ \|  \|  \| ✔️ \|  \| High \| \| A98 \| Sabbaghian et al \| 2014 \| Iran \| Cohort \| 134 \| 32.64 ± 0.64 \| ✔️ \|  \| ✔️ \|  \|  \|  \| High \| \| A99 \| Samplaski et al \| 2014 \| Canada \| Cohort \| 116 \| 37 \| ✔️ \| ✔️ \| ✔️ \|  \|  \|  \| High \| \| A100 \| Shanbhogue et al \| 2014 \| Denmark \| Cohort \| 31 \| 35.8 ± 8.2 \| ✔️ \|  \|  \|  \| ✔️ \|  \| High \| \| A101 \| Skakkebæk et al \| 2014 \| Denmark \| Cohort \| 73 \| 32.7 \| ✔️ \| ✔️ \|  \| ✔️ \|  \|  \| High \| \| A102 \| Skakkebæk et al \| 2014 \| Denmark \| Cohort \| 65 \| 36.8 ± 10.5 \|  \| ✔️ \|  \| ✔️ \|  \|  \| High \| \| A103 \| Chang et al \| 2015 \| Denmark \| Cohort \| 73 \| 18–60 \| ✔️ \| ✔️ \|  \|  \|  \|  \| High \| \| A104 \| Fedder et al \| 2015 \| Denmark \| Cohort \| 14 \| 31.8 ± 4.7 \| ✔️ \| ✔️ \| ✔️ \|  \|  \|  \| High \| \| A105 \| Ferlin et al \| 2015 \| Italy \| Cohort \| 127 \| 31.5 ± 8.5 \| ✔️ \| ✔️ \|  \|  \| ✔️ \|  \| High \| \| A106 \| Fisher et al \| *2015* \| Italy \| Cohort \| 46 \| 43.13 ± 13.78 \| ✔️ \| ✔️ \|  \| ✔️ \|  \|  \| High \| \| A107 \| Giagulli et al \| 2015 \| Italy \| Cohort \| 10 \| 50.6 ± 4.3 \| ✔️ \| ✔️ \|  \| ✔️ \| ✔️ \|  \| High \| \| A108 \| Gudeman et al \| 2015 \| USA \| Cohort \| 9 \| N/A \| ✔️ \|  \| ✔️ \|  \|  \|  \| High \| \| A109 \| Jaeger et al \| 2015 \| Sweden \| Cohort \| 8 \| 17-69 \|  \|  \|  \|  \|  \|  \| Medium \| \| A110 \| Jørgensen et al \| 2015 \| Denmark \| Cohort \| 62 \| 36.4 \| ✔️ \| ✔️ \|  \|  \|  \|  \| Medium \| \| A111 \| Plotton et al \| 2015 \| France \| Cohort \| 41 \| 24.0 ± 7.3 \| ✔️ \| ✔️ \| ✔️ \|  \|  \|  \| Medium \| \| A112 \| Rohayem et al \| 2015 \| Germany \| Cohort \| 85 \| 20-61 \| ✔️ \| ✔️ \| ✔️ \|  \|  \|  \| High \| \| A113 \| Turriff et al \| 2015 \| USA \| Cohort \| 310 \| 14–75 \|  \|  \| ✔️ \| ✔️ \|  \|  \| Medium \| \| A114 \| Belli et al \| 2016 \| Italy \| Cohort \| 13 \| 36 ± 9 \| ✔️ \| ✔️ \|  \|  \|  \|  \| High \| \| A115 \| Harris et al \| 2016 \| USA \| Cohort \| 4 \| N/A \|  \|  \|  \|  \| ✔️ \|  \| High \| \| A116 \| Majzoub et al \| 2016 \| Qatar \| Cohort \| 43 \| 32.9 ± 6.3 \| ✔️ \|  \| ✔️ \|  \|  \|  \| Medium \| \| A117 \| Nahata et al \| 2016 \| USA \| Cohort \| 15 \| 12-25 \| ✔️ \| ✔️ \| ✔️ \|  \| ✔️ \|  \| High \| \| A118 \| Rohayem et al \| 2016 \| Germany \| Cohort \| 281 \| 10-25 \| ✔️ \| ✔️ \| ✔️ \|  \|  \|  \| High \| \| A119 \| Wallentin et al \| 2016 \| Denmark \| Cohort \| 49 \| 35* \|  \|  \|  \| ✔️ \|  \|  \| Medium \| \| A120 \| Belling et al \| 2017 \| Denmark \| Population \| NK \| NK \|  \|  \|  \|  \| ✔️ \|  \|  \| \| A121 \| Binsaleh et al \| 2017 \| Saudi Arabia \| Cohort \| 11 \| 37.8 ± 3.3 \| ✔️ \| ✔️ \| ✔️ \|  \|  \|  \| High \| \| A122 \| Chehrazi et al \| 2017 \| Iran \| Cohort \| 134 \| 32.64 ± 0.64 \| ✔️ \|  \| ✔️ \|  \|  \|  \| Medium \| \| A123 \| Corona et al \| 2017 \| Italy \| Meta-analysis \| 1248 \| 30.9 ± 5.6 \| ✔️ \| ✔️ \| ✔️ \|  \|  \|  \| High \| \| A124 \| El Bardisi et al \| 2017 \| Qatar \| Cohort \| 53 \| 33.4 ± 6.8 \| ✔️ \| ✔️ \|  \| ✔️ \|  \|  \| Medium \| \| A125 \| Iwatsuki et al \| 2017 \| Japan \| Cohort \| 45 \| 34.5 ± 4.3 \| ✔️ \| ✔️ \| ✔️ \|  \| ✔️ \|  \| High \| \| A126 \| Ku et al \| 2017 \| Taiwan \| Cohort \| 24 \| 35.1 ± 4.5 \|  \|  \| ✔️ \|  \|  \|  \| Medium \| \| A127 \| Lee et al \| 2017 \| Korea \| Cohort \| 55 \| 34.5 ± 3.8 \| ✔️ \| ✔️ \|  \|  \| ✔️ \|  \| Medium \| \| A128 \| Liberato et al \| 2017 \| Italy \| Cohort \| 58 \| 34.6 ± 12.3 \| ✔️ \|  \|  \| ✔️ \|  \|  \| High \| \| A129 \| Olesen et al \| 2017 \| Denmark \| Cohort \| 11 \| 33.6* \| ✔️ \| ✔️ \| ✔️ \|  \| ✔️ \|  \| High \| \| A130 \| Rocher et al \| 2017 \| France \| Cohort \| 35 \| 33 ± 9.5 \|  \| ✔️ \|  \|  \|  \|  \| Medium \| \| A131 \| Skakkebæk et al \| 2017 \| Denmark \| Cohort \| 69 \| 36.4 \| ✔️ \|  \|  \| ✔️ \| ✔️ \|  \| High \| \| A132 \| Ali et al \| 2018 \| Egypt \| Cohort \| 8 \| 39.38 ± 9.1 \| ✔️ \| ✔️ \| ✔️ \|  \|  \|  \| Medium \| \| A133 \| Eken and Gulec \| 2018 \| Turkey \| Cohort \| 7 \| 33.40 ± 5.73 \| ✔️ \| ✔️ \| ✔️ \|  \|  \|  \| High \| \| A134 \| Eliveld et al \| 2018 \| Netherlands \| Meta-analysis \| 54 \| 25-61 \| ✔️ \| ✔️ \|  \|  \|  \|  \| High \| \| A135 \| Ferlin et al \| 2018 \| Italy \| Cohort \| 62 \| 31.2 ± 8.0 \| ✔️ \| ✔️ \|  \| ✔️ \| ✔️ \|  \| High \| \| A136 \| Garolla et al \| 2018 \| Italy \| Cohort \| 111 \| 29.9 ± 7.3 \| ✔️ \| ✔️ \| ✔️ \|  \|  \|  \| High \| \| A137 \| Kreukels et al \| 2018 \| Netherlands \| Multicentre \| 219 \| 39.4 \|  \|  \|  \| ✔️ \|  \|  \| Medium \| \| A138 \| Nordenström et al \| 2018 \| Sweden \| Multicentre \| 218 \| N/A \| ✔️ \| ✔️ \|  \|  \|  \|  \| Medium \| \| A139 \| Rapp et al \| 2018 \| Germany \| Multicentre \| 219 \| 32.4 ± 13.6 \|  \|  \|  \|  \|  \| ✔️ \| Medium \| \| A140 \| Skakkebæk et al \| 2018 \| Denmark \| Cohort \| 69 \| 36.4 ± 10 \| ✔️ \|  \|  \|  \|  \|  \| High \| \| A141 \| Skakkebæk et al \| 2018 \| Denmark \| Cohort \| 132 \| 41.7* \|  \| ✔️ \|  \| ✔️ \|  \| ✔️ \| High \| \| A142 \| Thyen et al \| 2018 \| Germany \| Multicentre \| 173 \| 37* \|  \|  \|  \|  \|  \| ✔️ \| High \| \| A143 \| van de Grift et al \| 2018 \| Netherlands \| Multicentre \| 225 \| 39.6 ± 15.1 \|  \| ✔️ \|  \| ✔️ \|  \|  \| High \| \| A144 \| van Rijn et al \| 2018 \| Netherlands \| Cohort \| 70 \| 31.2 ± 16.5 \|  \|  \|  \| ✔️ \|  \|  \| Medium \| \| A145 \| Van Saen et al \| 2018 \| Belgium \| Cohort \| 27 \| 18–41 \| ✔️ \|  \| ✔️ \|  \|  \|  \| High \| \| A146 \| Balercia et al \| 2019 \| Italy \| Multicentre \| 174 \| 40.6 ± 1 \| ✔️ \|  \|  \|  \| ✔️ \|  \| High \| \| A147 \| Chang et al \| 2019 \| Denmark \| Cohort \| 45 \| 41.55 \| ✔️ \| ✔️ \|  \|  \| ✔️ \|  \| High \| \| A148 \| de Vries et al \| 2019 \| Netherlands \| Multicentre \| 219 \| 39.4 ± 15.2 \|  \|  \|  \| ✔️ \|  \|  \| High \| \| A149 \| Granato et al \| 2019 \| Italy \| Cohort \| 221 \| 34.2 ± 12 \| ✔️ \| ✔️ \|  \|  \| ✔️ \|  \| High \| \| A150 \| Høst et al \| 2019 \| Denmark \| RCT \| 13 \| 34.8 ± 20.2 \| ✔️ \| ✔️ \|  \|  \| ✔️ \|  \| Medium \| \| A151 \| Mortensen et al \| 2019 \| Denmark \| Cohort \| 28 \| 34.8 ± 6.6 \| ✔️ \|  \|  \|  \|  \|  \| High \| \| A152 \| Santi et al \| 2019 \| Italy \| Meta-analysis \| 707 \| 28.21 ± 21.14 \| ✔️ \|  \|  \|  \|  \|  \| High \| \| A153 \| Deebel et al \| 2020 \| USA \| Meta-analysis \| 152 \| 18-66 \| ✔️ \|  \| ✔️ \|  \|  \|  \| Medium \| \| A154 \| Guo et al \| 2020 \| China \| Cohort \| 184 \| 30.2 ± 4.8 \| ✔️ \|  \| ✔️ \|  \|  \|  \| High \| \| A155 \| Hussein et al \| 2020 \| Egypt \| Cohort \| 85 \| 30.75 ± 6.93 \| ✔️ \|  \|  \|  \| ✔️ \|  \| High \| \| A156 \| Madian et al \| 2020 \| Egypt \| Cohort \| 14 \| 28.4 ± 4.9 \| ✔️ \| ✔️ \| ✔️ \|  \|  \|  \| Medium \| \| A157 \| Pizzocaro et al \| 2020 \| Italy \| Meta-analysis \| 1144 \| 31.1 \| ✔️ \| ✔️ \|  \|  \| ✔️ \|  \| High \| \| A158 \| Pozzi et al \| 2020 \| Italy \| Multicentre \| 103 \| 32.0* \| ✔️ \| ✔️ \| ✔️ \|  \| ✔️ \|  \| High \| \| A159 \| Slowikowska-Hilczer et al \| 2020 \| Poland \| Multicentre \| 218 \| 29.9 \|  \| ✔️ \|  \|  \| ✔️ \|  \| Medium \| \| A160 \| Van Saen et al \| 2020 \| Belgium \| Cohort \| 27 \| N/A \|  \|  \| ✔️ \|  \|  \|  \| High \| \| A161 \| Vena et al \| 2020 \| Italy \| Cohort \| 87 \| 41* \| ✔️ \|  \|  \|  \| ✔️ \|  \| High \| \| A162 \| Barbonetti et al \| 2021 \| Italy \| Meta-analysis \| 482 \| 35.8 \| ✔️ \|  \|  \| ✔️ \| ✔️ \|  \| Medium \| \| A163 \| Fabrazzo et al \| 2021 \| Italy \| Cohort \| 23 \| 36.2 ± 9.4 \| ✔️ \|  \|  \| ✔️ \|  \| ✔️ \| High \| \| A164 \| Indirli et al \| 2021 \| Italy \| Multicentre \| 58 \| 39.7 * \| ✔️ \| ✔️ \|  \|  \| ✔️ \|  \| Medium \| \| A165 \| Özman et al \| 2021 \| Turkey \| Cohort \| 14 \| 33.8 \| ✔️ \| ✔️ \| ✔️ \|  \|  \|  \| High \| \| A166 \| Rapp et al \| 2021 \| Sweden \| Multicentre \| 218 \| 43 \|  \|  \|  \|  \|  \|  \| Medium \| \| A167 \| Chu et al \| 2022 \| USA \| Multicentre \| 116 \| 16.5 ± 2.3 \| ✔️ \| ✔️ \| ✔️ \| ✔️ \| ✔️ \|  \| High \| \| A168 \| de Brouwer et al \| 2022 \| Netherlands \| Multicentre \| 206 \| 38* \|  \|  \|  \| ✔️ \|  \|  \| Medium \| \| A169 \| Deebel et al \| 2022 \| USA \| Cohort \| 79 \| 32.9 ± 0.7 \| ✔️ \| ✔️ \| ✔️ \|  \|  \|  \| Medium \| \| A170 \| Giovanelli et al \| 2022 \| Italy \| Cohort \| 46 \| 39.2 ± 13.2 \| ✔️ \| ✔️ \|  \|  \| ✔️ \|  \| High \| \| A171 \| Kızılay et al \| 2022 \| Turkey \| Multicentre \| 142 \| 32.2 \| ✔️ \| ✔️ \| ✔️ \|  \| ✔️ \|  \| High \| \| A172 \| Majzoub et al \| 2022 \| Qatar \| Meta-analysis \| 2239 \| N/A \| ✔️ \| ✔️ \| ✔️ \|  \|  \|  \| High \| \| A173 \| Özkan et al \| 2022 \| Turkey \| Cohort \| 67 \| 22-47 \| ✔️ \| ✔️ \| ✔️ \|  \|  \|  \| High \| \| A174 \| Pasquali et al \| 2022 \| Italy \| Cohort \| 609 \| 37.4 ± 13.4 \| ✔️ \| ✔️ \|  \|  \| ✔️ \|  \| Medium \| \| A175 \| Renault et al \| 2022 \| France \| Cohort \| 119 \| 22.4* \| ✔️ \| ✔️ \| ✔️ \|  \|  \|  \| High \| \| A176 \| van de Grift et al \| 2022 \| Netherlands \| Multicentre \| 57 \| 16 - ≥65 \|  \|  \|  \|  \|  \|  \| High \| \| A177 \| Zhao et al \| 2022 \| United Kingdom \| Cohort \| 213 \| 40-70 \| ✔️ \| ✔️ \|  \| ✔️ \| ✔️ \|  \| High \| \| A178 \| Demirci et al \| 2023 \| Turkey \| Cohort \| 30 \| 21.53 ± 1.66 \| ✔️ \| ✔️ \|  \|  \|  \|  \| Medium \| \| A179 \| Eliveld et al \| 2023 \| Netherlands \| Cohort \| 11 \| 33.36 ± 5.7 \| ✔️ \| ✔️ \| ✔️ \|  \|  \|  \| Medium \| \| A180 \| Fjermestad et al \| 2023 \| Norway \| Cohort \| 26 \| 37.0 ± 10.9 \|  \|  \|  \| ✔️ \|  \|  \| High \| \| A181 \| Franik et al \| 2023 \| Europe \| Cohort \| 205 \| 39.9 \|  \|  \|  \|  \| ✔️ \|  \| High \| \| A182 \| Sánchez et al \| 2023 \| Denmark \| Cohort \| 21 \| 0-34.7 \|  \|  \|  \| ✔️ \| ✔️ \|  \| High \| |
| --- | --- | --- | --- | --- | --- | --- | --- | --- | --- | --- | --- | --- | --- | --- | --- | --- | --- | --- | --- | --- | --- | --- | --- | --- | --- | --- | --- | --- | --- | --- | --- | --- | --- | --- | --- | --- | --- | --- | --- | --- | --- | --- | --- | --- | --- | --- | --- | --- | --- | --- | --- | --- | --- | --- | --- | --- | --- | --- | --- | --- | --- | --- | --- | --- | --- | --- | --- | --- | --- | --- | --- | --- | --- | --- | --- | --- | --- | --- | --- | --- | --- | --- | --- | --- | --- | --- | --- | --- | --- | --- | --- | --- | --- | --- | --- | --- | --- | --- | --- | --- | --- | --- | --- | --- | --- | --- | --- | --- | --- | --- | --- | --- | --- | --- | --- | --- | --- | --- | --- | --- | --- | --- | --- | --- | --- | --- | --- | --- | --- | --- | --- | --- | --- | --- | --- | --- | --- | --- | --- | --- | --- | --- | --- | --- | --- | --- | --- | --- | --- | --- | --- | --- | --- | --- | --- | --- | --- | --- | --- | --- | --- | --- | --- | --- | --- | --- | --- | --- | --- | --- | --- | --- | --- | --- | --- | --- | --- | --- | --- | --- | --- | --- | --- | --- | --- | --- | --- | --- | --- | --- | --- | --- | --- | --- | --- | --- | --- | --- | --- | --- | --- | --- | --- | --- | --- | --- | --- | --- | --- | --- | --- | --- | --- | --- | --- | --- | --- | --- | --- | --- | --- | --- | --- | --- | --- | --- | --- | --- | --- | --- | --- | --- | --- | --- | --- | --- | --- | --- | --- | --- | --- | --- | --- | --- | --- | --- | --- | --- | --- | --- | --- | --- | --- | --- | --- | --- | --- | --- | --- | --- | --- | --- | --- | --- | --- | --- | --- | --- | --- | --- | --- | --- | --- | --- | --- | --- | --- | --- | --- | --- | --- | --- | --- | --- | --- | --- | --- | --- | --- | --- | --- | --- | --- | --- | --- | --- | --- | --- | --- | --- | --- | --- | --- | --- | --- | --- | --- | --- | --- | --- | --- | --- | --- | --- | --- | --- | --- | --- | --- | --- | --- | --- | --- | --- | --- | --- | --- | --- | --- | --- | --- | --- | --- | --- | --- | --- | --- | --- | --- | --- | --- | --- | --- | --- | --- | --- | --- | --- | --- | --- | --- | --- | --- | --- | --- | --- | --- | --- | --- | --- | --- | --- | --- | --- | --- | --- | --- | --- | --- | --- | --- | --- | --- | --- | --- | --- | --- | --- | --- | --- | --- | --- | --- | --- | --- | --- | --- | --- | --- | --- | --- | --- | --- | --- | --- | --- | --- | --- | --- | --- | --- | --- | --- | --- | --- | --- | --- | --- | --- | --- | --- | --- | --- | --- | --- | --- | --- | --- | --- | --- | --- | --- | --- | --- | --- | --- | --- | --- | --- | --- | --- | --- | --- | --- | --- | --- | --- | --- | --- | --- | --- | --- | --- | --- | --- | --- | --- | --- | --- | --- | --- | --- | --- | --- | --- | --- | --- | --- | --- | --- | --- | --- | --- | --- | --- | --- | --- | --- | --- | --- | --- | --- | --- | --- | --- | --- | --- | --- | --- | --- | --- | --- | --- | --- | --- | --- | --- | --- | --- | --- | --- | --- | --- | --- | --- | --- | --- | --- | --- | --- | --- | --- | --- | --- | --- | --- | --- | --- | --- | --- | --- | --- | --- | --- | --- | --- | --- | --- | --- | --- | --- | --- | --- | --- | --- | --- | --- | --- | --- | --- | --- | --- | --- | --- | --- | --- | --- | --- | --- | --- | --- | --- | --- | --- | --- | --- | --- | --- | --- | --- | --- | --- | --- | --- | --- | --- | --- | --- | --- | --- | --- | --- | --- | --- | --- | --- | --- | --- | --- | --- | --- | --- | --- | --- | --- | --- | --- | --- | --- | --- | --- | --- | --- | --- | --- | --- | --- | --- | --- | --- | --- | --- | --- | --- | --- | --- | --- | --- | --- | --- | --- | --- | --- | --- | --- | --- | --- | --- | --- | --- | --- | --- | --- | --- | --- | --- | --- | --- | --- | --- | --- | --- | --- | --- | --- | --- | --- | --- | --- | --- | --- | --- | --- | --- | --- | --- | --- | --- | --- | --- | --- | --- | --- | --- | --- | --- | --- | --- | --- | --- | --- | --- | --- | --- | --- | --- | --- | --- | --- | --- | --- | --- | --- | --- | --- | --- | --- | --- | --- | --- | --- | --- | --- | --- | --- | --- | --- | --- | --- | --- | --- | --- | --- | --- | --- | --- | --- | --- | --- | --- | --- | --- | --- | --- | --- | --- | --- | --- | --- | --- | --- | --- | --- | --- | --- | --- | --- | --- | --- | --- | --- | --- | --- | --- | --- | --- | --- | --- | --- | --- | --- | --- | --- | --- | --- | --- | --- | --- | --- | --- | --- | --- | --- | --- | --- | --- | --- | --- | --- | --- | --- | --- | --- | --- | --- | --- | --- | --- | --- | --- | --- | --- | --- | --- | --- | --- | --- | --- | --- | --- | --- | --- | --- | --- | --- | --- | --- | --- | --- | --- | --- | --- | --- | --- | --- | --- | --- | --- | --- | --- | --- | --- | --- | --- | --- | --- | --- | --- | --- | --- | --- | --- | --- | --- | --- | --- | --- | --- | --- | --- | --- | --- | --- | --- | --- | --- | --- | --- | --- | --- | --- | --- | --- | --- | --- | --- | --- | --- | --- | --- | --- | --- | --- | --- | --- | --- | --- | --- | --- | --- | --- | --- | --- | --- | --- | --- | --- | --- | --- | --- | --- | --- | --- | --- | --- | --- | --- | --- | --- | --- | --- | --- | --- | --- | --- | --- | --- | --- | --- | --- | --- | --- | --- | --- | --- | --- | --- | --- | --- | --- | --- | --- | --- | --- | --- | --- | --- | --- | --- | --- | --- | --- | --- | --- | --- | --- | --- | --- | --- | --- | --- | --- | --- | --- | --- | --- | --- | --- | --- | --- | --- | --- | --- | --- | --- | --- | --- | --- | --- | --- | --- | --- | --- | --- | --- | --- | --- | --- | --- | --- | --- | --- | --- | --- | --- | --- | --- | --- | --- | --- | --- | --- | --- | --- | --- | --- | --- | --- | --- | --- | --- | --- | --- | --- | --- | --- | --- | --- | --- | --- | --- | --- | --- | --- | --- | --- | --- | --- | --- | --- | --- | --- | --- | --- | --- | --- | --- | --- | --- | --- | --- | --- | --- | --- | --- | --- | --- | --- | --- | --- | --- | --- | --- | --- | --- | --- | --- | --- | --- | --- | --- | --- | --- | --- | --- | --- | --- | --- | --- | --- | --- | --- | --- | --- | --- | --- | --- | --- | --- | --- | --- | --- | --- | --- | --- | --- | --- | --- | --- | --- | --- | --- | --- | --- | --- | --- | --- | --- | --- | --- | --- | --- | --- | --- | --- | --- | --- | --- | --- | --- | --- | --- | --- | --- | --- | --- | --- | --- | --- | --- | --- | --- | --- | --- | --- | --- | --- | --- | --- | --- | --- | --- | --- | --- | --- | --- | --- | --- | --- | --- | --- | --- | --- | --- | --- | --- | --- | --- | --- | --- | --- | --- | --- | --- | --- | --- | --- | --- | --- | --- | --- | --- | --- | --- | --- | --- | --- | --- | --- | --- | --- | --- | --- | --- | --- | --- | --- | --- | --- | --- | --- | --- | --- | --- | --- | --- | --- | --- | --- | --- | --- | --- | --- | --- | --- | --- | --- | --- | --- | --- | --- | --- | --- | --- | --- | --- | --- | --- | --- | --- | --- | --- | --- | --- | --- | --- | --- | --- | --- | --- | --- | --- | --- | --- | --- | --- | --- | --- | --- | --- | --- | --- | --- | --- | --- | --- | --- | --- | --- | --- | --- | --- | --- | --- | --- | --- | --- | --- | --- | --- | --- | --- | --- | --- | --- | --- | --- | --- | --- | --- | --- | --- | --- | --- | --- | --- | --- | --- | --- | --- | --- | --- | --- | --- | --- | --- | --- | --- | --- | --- | --- | --- | --- | --- | --- | --- | --- | --- | --- | --- | --- | --- | --- | --- | --- | --- | --- | --- | --- | --- | --- | --- | --- | --- | --- | --- | --- | --- | --- | --- | --- | --- | --- | --- | --- | --- | --- | --- | --- | --- | --- | --- | --- | --- | --- | --- | --- | --- | --- | --- | --- | --- | --- | --- | --- | --- | --- | --- | --- | --- | --- | --- | --- | --- | --- | --- | --- | --- | --- | --- | --- | --- | --- | --- | --- | --- | --- | --- | --- | --- | --- | --- | --- | --- | --- | --- | --- | --- | --- | --- | --- | --- | --- | --- | --- | --- | --- | --- | --- | --- | --- | --- | --- | --- | --- | --- | --- | --- | --- | --- | --- | --- | --- | --- | --- | --- | --- | --- | --- | --- | --- | --- | --- | --- | --- | --- | --- | --- | --- | --- | --- | --- | --- | --- | --- | --- | --- | --- | --- | --- | --- | --- | --- | --- | --- | --- | --- | --- | --- | --- | --- | --- | --- | --- | --- | --- | --- | --- | --- | --- | --- | --- | --- | --- | --- | --- | --- | --- | --- | --- | --- | --- | --- | --- | --- | --- | --- | --- | --- | --- | --- | --- | --- | --- | --- | --- | --- | --- | --- | --- | --- | --- | --- | --- | --- | --- | --- | --- | --- | --- | --- | --- | --- | --- | --- | --- | --- | --- | --- | --- | --- | --- | --- | --- | --- | --- | --- | --- | --- | --- | --- | --- | --- | --- | --- | --- | --- | --- | --- | --- | --- | --- | --- | --- | --- | --- | --- | --- | --- | --- | --- | --- | --- | --- | --- | --- | --- | --- | --- | --- | --- | --- | --- | --- | --- | --- | --- | --- | --- | --- | --- | --- | --- | --- | --- | --- | --- | --- | --- | --- | --- | --- | --- | --- | --- | --- | --- | --- | --- | --- | --- | --- | --- | --- | --- | --- | --- | --- | --- | --- | --- | --- | --- | --- | --- | --- | --- | --- | --- | --- | --- | --- | --- | --- | --- | --- | --- | --- | --- | --- | --- | --- | --- | --- | --- | --- | --- | --- | --- | --- | --- | --- | --- | --- | --- | --- | --- | --- | --- | --- | --- | --- | --- | --- | --- | --- | --- | --- | --- | --- | --- | --- | --- | --- | --- | --- | --- | --- | --- | --- | --- | --- | --- | --- | --- | --- | --- | --- | --- | --- | --- | --- | --- | --- | --- | --- | --- | --- | --- | --- | --- | --- | --- | --- | --- | --- | --- | --- | --- | --- | --- | --- | --- | --- | --- | --- | --- | --- | --- | --- | --- | --- | --- | --- | --- | --- | --- | --- | --- | --- | --- | --- | --- | --- | --- | --- | --- | --- | --- | --- | --- | --- | --- | --- | --- | --- | --- | --- | --- | --- | --- | --- | --- | --- | --- | --- | --- | --- | --- | --- | --- | --- | --- | --- | --- | --- | --- | --- | --- | --- | --- | --- | --- | --- | --- | --- | --- | --- | --- | --- | --- | --- | --- | --- | --- | --- | --- | --- | --- | --- | --- | --- | --- | --- | --- | --- | --- | --- | --- | --- | --- | --- | --- | --- | --- | --- | --- | --- | --- | --- | --- | --- | --- | --- | --- | --- | --- | --- | --- | --- | --- | --- | --- | --- | --- | --- | --- | --- | --- | --- | --- | --- | --- | --- | --- | --- | --- | --- | --- | --- | --- | --- | --- | --- | --- | --- | --- | --- | --- | --- | --- | --- | --- | --- | --- | --- | --- | --- | --- | --- | --- | --- | --- | --- | --- | --- | --- | --- | --- | --- | --- | --- | --- | --- | --- | --- | --- | --- | --- | --- | --- | --- | --- | --- | --- | --- | --- | --- | --- | --- | --- | --- | --- | --- | --- | --- | --- | --- | --- | --- | --- | --- | --- | --- | --- | --- | --- | --- | --- | --- | --- | --- | --- | --- | --- | --- | --- | --- | --- | --- | --- | --- | --- | --- | --- | --- | --- | --- | --- | --- | --- | --- | --- | --- | --- | --- | --- | --- | --- | --- | --- | --- | --- | --- | --- | --- | --- | --- | --- | --- | --- | --- | --- | --- | --- | --- | --- | --- | --- | --- | --- | --- | --- | --- | --- | --- | --- | --- | --- | --- | --- | --- | --- | --- | --- | --- | --- | --- | --- | --- | --- | --- | --- | --- | --- | --- | --- | --- | --- | --- | --- | --- | --- | --- | --- | --- | --- | --- | --- | --- | --- | --- | --- | --- | --- | --- | --- | --- | --- | --- | --- | --- | --- | --- | --- | --- | --- | --- | --- | --- | --- | --- | --- | --- | --- | --- | --- | --- | --- | --- | --- | --- | --- | --- | --- | --- | --- | --- | --- | --- | --- | --- | --- | --- | --- | --- | --- | --- | --- | --- | --- | --- | --- | --- | --- | --- | --- | --- | --- | --- | --- | --- | --- | --- | --- | --- | --- | --- | --- | --- | --- | --- | --- | --- | --- | --- | --- | --- | --- | --- | --- | --- | --- | --- | --- | --- | --- | --- | --- | --- | --- | --- | --- | --- | --- | --- | --- | --- | --- | --- | --- | --- | --- | --- | --- | --- | --- | --- | --- | --- | --- | --- | --- | --- | --- | --- | --- | --- | --- | --- | --- | --- | --- | --- | --- | --- | --- | --- | --- | --- | --- | --- | --- | --- | --- | --- | --- | --- | --- | --- | --- | --- | --- | --- | --- | --- | --- | --- | --- | --- | --- | --- | --- | --- | --- | --- | --- | --- | --- | --- | --- | --- | --- | --- | --- | --- | --- | --- | --- | --- | --- | --- | --- | --- | --- | --- | --- | --- | --- | --- | --- | --- | --- | --- | --- | --- | --- | --- | --- | --- | --- | --- | --- | --- | --- | --- | --- | --- | --- | --- | --- | --- | --- | --- | --- | --- | --- | --- | --- | --- | --- | --- | --- | --- | --- | --- | --- | --- | --- | --- | --- | --- | --- | --- | --- | --- | --- | --- | --- | --- | --- | --- | --- | --- | --- | --- | --- | --- | --- | --- | --- | --- | --- | --- | --- | --- | --- | --- | --- | --- | --- | --- | --- | --- | --- | --- | --- | --- | --- | --- | --- | --- | --- | --- | --- | --- | --- | --- | --- | --- | --- | --- | --- | --- | --- | --- | --- | --- | --- | --- | --- | --- | --- | --- | --- | --- | --- | --- | --- | --- | --- | --- | --- | --- | --- | --- | --- | --- | --- | --- | --- | --- | --- | --- | --- | --- | --- | --- | --- | --- | --- | --- | --- | --- | --- | --- | --- | --- | --- | --- | --- | --- | --- | --- | --- | --- | --- | --- | --- | --- | --- | --- | --- | --- | --- | --- | --- | --- | --- | --- | --- | --- | --- | --- | --- | --- | --- | --- | --- | --- | --- | --- | --- | --- | --- | --- | --- | --- | --- | --- | --- | --- | --- | --- | --- | --- | --- | --- | --- | --- | --- | --- | --- | --- | --- | --- | --- | --- | --- | --- | --- | --- | --- | --- | --- | --- | --- | --- | --- | --- | --- | --- | --- | --- | --- | --- | --- | --- | --- | --- | --- | --- | --- | --- | --- | --- | --- | --- | --- | --- | --- | --- | --- | --- | --- | --- | --- | --- | --- | --- | --- | --- | --- | --- | --- | --- | --- | --- | --- | --- | --- | --- | --- | --- | --- | --- | --- | --- | --- | --- | --- | --- | --- | --- | --- | --- | --- | --- | --- | --- | --- | --- | --- | --- | --- | --- | --- | --- | --- | --- | --- | --- | --- | --- | --- | --- | --- | --- | --- | --- | --- | --- | --- | --- | --- | --- | --- | --- | --- | --- | --- | --- | --- | --- | --- | --- | --- | --- | --- | --- | --- | --- | --- | --- | --- | --- | --- | --- | --- | --- | --- | --- | --- | --- | --- | --- | --- | --- | --- | --- | --- | --- | --- | --- | --- | --- | --- | --- | --- | --- | --- | --- | --- | --- | --- | --- | --- | --- | --- | --- | --- | --- | --- | --- | --- | --- | --- | --- | --- | --- | --- | --- | --- | --- | --- | --- | --- | --- | --- | --- | --- | --- | --- | --- | --- | --- | --- | --- | --- | --- | --- | --- | --- | --- | --- | --- | --- | --- | --- | --- | --- | --- | --- | --- | --- | --- | --- | --- | --- | --- | --- | --- | --- | --- | --- | --- | --- | --- | --- | --- | --- | --- | --- | --- | --- | --- | --- | --- | --- | --- | --- | --- | --- | --- | --- | --- | --- | --- | --- | --- | --- | --- | --- | --- | --- | --- | --- | --- | --- | --- | --- | --- | --- | --- | --- | --- | --- | --- | --- | --- | --- | --- | --- | --- | --- | --- | --- | --- | --- | --- | --- | --- | --- | --- | --- | --- | --- | --- | --- | --- | --- | --- | --- | --- | --- | --- | --- | --- | --- | --- | --- | --- | --- | --- | --- | --- | --- | --- | --- | --- | --- | --- | --- | --- | --- | --- | --- | --- | --- | --- | --- | --- | --- | --- | --- | --- | --- | --- | --- | --- | --- | --- | --- | --- | --- | --- |

Supplementary Table 3. Table summarising the 182 included studies in men ≥16 years with KS. Data shown includes the author, year, country of origin, type of study, participant number, mean age ± standard deviation (SD) (years) or median* or range if mean/median were unavailable. Outcome data included biochemistry (Bio), phenotype/anthropometry (PA), fertility (Fe), cognitive, psychological, developmental and behavioural (CP), co-morbidities (CM) and quality of life (QoL). The quality of each study was assessed as high, medium, or low based on the relevant Critical Appraisal Skills Programme checklists.
